# Thoracic epidural analgesia in intensive care unit patients with acute pancreatitis: the EPIPAN multicenter randomized controlled trial

**DOI:** 10.1186/s13054-023-04502-w

**Published:** 2023-05-31

**Authors:** Matthieu Jabaudon, Alexandra Genevrier, Samir Jaber, Olivier Windisch, Stéphanie Bulyez, Pierre-François Laterre, Etienne Escudier, Achille Sossou, Philippe Guerci, Pierre-Marie Bertrand, Pierre-Eric Danin, Martin Bonnassieux, Leo Bühler, Claudia Paula Heidegger, Russell Chabanne, Thomas Godet, Laurence Roszyk, Vincent Sapin, Emmanuel Futier, Bruno Pereira, Jean-Michel Constantin, for the EPIPAN study group, Elodie Caumon, Julien Amat, Dominique Morand, Renaud Guérin, Sébastien Perbet, Benjamin Rieu, Sophie Cayot, Christian Chartier, Camille Verlhac, Christine Rolhion, Justine Bourdier, Bernard Cosserant, Raiko Blondonnet, Jean-Baptiste Joffredo, Thomas Costilles, Damien Bouvier, Lise Bernard, Jean-Etienne Bazin, Laurence Roszyk, Lydie Marie-Anne, Raphaël Giraud, Annick Puchois, Cyril Boronad, Marine Agullo, Boris Jung, Gérald Chanques, Cécile Spirito, Marion Monnin, Albert Prades, Moussa Cisse, Anne Verchere, Claudine Gniadek, Fouad Belafia, Daniel Verzilli, Julie Carr, Audrey De Jong, Yannael Coisel, Jean-Marc Delay, Matthieu Conseil, Marie Gonzalez, Delphine Rosant, Michel Prevot, Bernard Claud, François Brenas, Lassane Zanre, Philippe Bray, Hélène Riera, Emilie Gadea-Deschamps, Pablo Massanet, Caroline Boutin, Saber Barbar, David-Paul De Brauwere, Serge Lumbroso, Amélie Maurin, Sophie Lloret, Laurent Muller, Claire Roger, Jean-Yves Lefrant, Loubna Elotmani, Audrey Ayral, Suzanne Renard, Nadège Bouskila, Gaspard Beaune, Magali Farines-Raffoul, Marie Lebouc, Auguste Dargent, Thomas Crozon, Julien Clauzel, Marinne Le Core, Thomas Rimmelé

**Affiliations:** 1 Department of Perioperative Medicine, CHU Clermont-Ferrand, 58 Rue Montalembert, 63000 Clermont-Ferrand, France; 2 iGReD, CNRS, INSERM, Université Clermont Auvergne, Clermont-Ferrand, France; 3 Saint Eloi Intensive Care Unit, CHU Montpellier, Montpellier, France; 4 PhyMedExp, Université de Montpellier, INSERM, CNRS, Montpellier, France; 5 Department of Surgery, Geneva University Hospitals, Geneva, Switzerland; 6 Faculty of Medicine, University of Geneva, Geneva, Switzerland; 7 Division of Intensive Care, Department of Acute Medicine, Geneva University Hospitals, Geneva, Switzerland; 8 Service de Recherche Clinique en Soins Critiques, Pôle Anesthésie Douleur Urgences Réanimation, CHU Nîmes, Université de Montpellier, Nîmes, France; 9 Department of Critical Care Medicine, Saint Luc University Hospital, Université Catholique de Louvain, Brussels, Belgium; 10 Department of Emergency Medicine and Intensive Care, Annecy Genevois General Hospital, Annecy, France; 11 Department of Intensive Care Medicine, Emile-Roux General Hospital, Le Puy-en-Velay, France; 12 Department of Anesthesiology and Critical Care Medicine, CHU Nancy-Brabois, Nancy, France; 13 Institut Lorrain du Coeur Et Des Vaisseaux and INSERM U1116, Institut Lorrain du Coeur et des Vaisseaux, University of Lorraine, Nancy, France; 14 Department of Intensive Care Medicine, Cannes General Hospital, Cannes, France; 15 Department of Intensive Care Medicine, CHU Nice, Nice, France; 16 INSERM U1065, Team 8, C3M, CHU de Nice, Nice, France; 17 Department of Intensive Care Medicine, Hôpital Edouard Herriot, Hospices Civils de Lyon, Lyon, France; 18 Department of Biochemistry and Molecular Genetics, CHU Clermont-Ferrand, Clermont-Ferrand, France; 19 Biostatistics and Data Management Unit, Department of Clinical Research and Innovation, CHU Clermont-Ferrand, Clermont-Ferrand, France; 20 Department of Anesthesiology and Critical Care, GRC 29, DMU DREAM, Pitié-Salpêtrière Hospital, Sorbonne University, Assistance Publique-Hôpitaux de Paris, Paris, France; 21 CHU Clermont-Ferrand, Clermont-Ferrand, France; 22 Geneva University Hospitals, Geneva, Switzerland; 23 Cannes General Hospital, Cannes, France; 24 CHU Montpellier, Montpellier, France; 25 CHU Nancy-Brabois, Nancy, France; 26 Emile-Roux General Hospital, Le Puy-en-Velay, France; 27 CHU Nîmes, Nîmes, France; 28 Saint Luc University Hospital, Université Catholique de Louvain, Brussels, Belgium; 29 Nice Archet 2 University Hospital, Nice, France; 30 Annecy Genevois General Hospital, Annecy, France; 31 Hospices Civils de Lyon, Lyon, France

**Keywords:** Acute pancreatitis, Intensive care unit, Epidural analgesia, Ventilator-free days

## Abstract

**Background:**

Findings from preclinical studies and one pilot clinical trial suggest potential benefits of epidural analgesia in acute pancreatitis. We aimed to assess the efficacy of thoracic epidural analgesia, in addition to usual care, in improving clinical outcomes of intensive care unit patients with acute pancreatitis.

**Methods:**

A multicenter, open-label, randomized, controlled trial including adult patients with a clinical diagnosis of acute pancreatitis upon admission to the intensive care unit. Participants were randomly assigned (1:1) to a strategy combining thoracic epidural analgesia and usual care (intervention group) or a strategy of usual care alone (control group). The primary outcome was the number of ventilator-free days from randomization until day 30.

**Results:**

Between June 2014 and January 2019, 148 patients were enrolled, and 135 patients were included in the intention-to-treat analysis, with 65 patients randomly assigned to the intervention group and 70 to the control group. The number of ventilator-free days did not differ significantly between the intervention and control groups (median [interquartile range], 30 days [15–30] and 30 days [18–30], respectively; median absolute difference of − 0.0 days, 95% CI − 3.3 to 3.3; *p* = 0.59). Epidural analgesia was significantly associated with longer duration of invasive ventilation (median [interquartile range], 14 days [5–28] versus 6 days [2–13], *p* = 0.02).

**Conclusions:**

In a population of intensive care unit adults with acute pancreatitis and low requirement for intubation, this first multicenter randomized trial did not show the hypothesized benefit of epidural analgesia in addition to usual care. Safety of epidural analgesia in this setting requires further investigation.

*Trial registration*: ClinicalTrials.gov registration number NCT02126332, April 30, 2014.

**Supplementary Information:**

The online version contains supplementary material available at 10.1186/s13054-023-04502-w.

## Background

Acute pancreatitis may develop under a severe form comprising persistent organ failure and requiring admission to the intensive care unit [1, 2]. In a French observational study, tracheal intubation and invasive mechanical ventilation were needed in 58% of intensive care unit patients with severe acute pancreatitis, with a higher mortality rate than in those who did not require intubation (34.0% vs. 1.4%, respectively) or who had fewer ventilator-free days [3, 4]. The multidisciplinary management of acute pancreatitis has substantially improved in recent years and pain management is a pivotal element of current recommendations for usual care [2, 5–7]. However, no analgesic strategy has been proven superior in terms of efficacy and safety [8–10].

Epidural analgesia is widely used for analgesia during labor or major surgery, and after surgery or trauma in some intensive care unit patients [11]. In animal studies, epidural analgesia has organ-protective effects which could be clinically relevant [12]. Observational studies did not find obvious adverse events attributable to epidural analgesia in intensive care unit patients with acute pancreatitis and its use, although infrequent, was associated with decreased mortality in a multicenter, retrospective propensity analysis [13–15]. In a single-center randomized trial, thoracic epidural analgesia improved pancreas perfusion on computed tomography, with a nonsignificant decrease in the need for intubation, compared to a control strategy without epidural analgesia [16]. Although epidural analgesia is used in some intensive care unit patients with acute pancreatitis to treat pain while potentially decreasing opioid consumption, its impact on clinical outcomes remains unknown.

Based on the hypothesis that epidural analgesia could influence clinical outcome, we conducted the multicenter EPIPAN (epidural analgesia for acute pancreatitis) trial to determine whether thoracic epidural analgesia combined with usual care would result in more ventilator-free days than usual care alone in intensive care unit adults with acute pancreatitis, considering ventilator-free days as an endpoint that reflects the need for intubation and the duration of invasive mechanical ventilation when needed, while accounting for death as a competing risk [17, 18].

## Methods

### Study design and participants

This pragmatic, multicenter, randomized, controlled, open-label, and parallel group superiority trial enrolled adult patients with a clinical diagnosis of acute pancreatitis upon admission to one the 11 participating intensive care units from France, Switzerland, and Belgium. The diagnosis of acute pancreatitis required two of the following three features, as per the revised Atlanta definition [1]: abdominal pain consistent with acute pancreatitis, serum lipase activity at least three times greater than the upper limit of normal, and characteristic findings of acute pancreatitis on contrast-enhanced computed tomography.

The trial design has been published previously [19]. All authors had access to the study data and reviewed and approved the final manuscript. All patients or their legal representatives provided written informed consent. The study protocol was approved by the French Ethics Committee (*Comité de Protection des Personnes Sud-Est VI*; approval AU1090) and Medicine Agency (*Agence Nationale de Sécurité du Médicament*; approval 131557A-32), as well as all participating centers. The study was performed in accordance with the 2008 Declaration of Helsinki and its later amendments.

Patients fulfilling one or more of the following criteria were not included: prothrombin time < 60%, platelet count < 75 G/L, curative anticoagulant therapy with heparin interrupted for less than 8 h, local infection, active central nervous system infection, history of back surgery associated with a dural space procedure, suspected or confirmed intracranial hypertension, refractory circulatory shock despite appropriate resuscitation, known allergy to clonidine, ropivacaine or sufentanil, treatment with a monoamine oxidase inhibitor in the previous 15 days, age under 18 or under tutelage measures, and absence of coverage by the French health insurance system.

### Randomization

Patients were randomized to receive usual care plus epidural analgesia for at least 72 h (intervention group) or usual care alone (control group). Randomization was stratified by site, duration of symptoms (< 48 vs. ≥ 48 h), and severity as assessed by the modified Marshall scoring system for organ dysfunction (three strata of increasing severity were defined according to the maximum score obtained for at least one of the respiratory, renal, or hemodynamic functions) [1].

### Procedures

Patients assigned to epidural analgesia and usual care received thoracic epidural analgesia as soon as possible after randomization. An epidural catheter was placed in an intervertebral space between the sixth and ninth thoracic vertebra by a certified anesthesiologist-intensivist or a resident in anesthesiology and intensive care, under the supervision of a certified anesthesiologist-intensivist. A mixed solution of ropivacaine (2 mg/mL) and sufentanil (0.5 μg/mL) was administered for at least 72 h using a patient-controlled epidural analgesia system with continuous infusion rates set between 5 and 15 mL/h and boli of 3 to 10 mL every 10 min at maximum. Nurses were encouraged to administer boli to achieve analgesia goals when the patient was not able to self-administer. Supplemental iterative epidural administrations of clonidine (1 μg/kg) were allowed to achieve analgesia goals. The duration and weaning of epidural analgesia, as well as removal of the epidural catheter, were conducted according to routine protocols from each participating center.

Patients assigned to usual care alone did not receive epidural analgesia. Usual care was based on current consensual guidelines [6]. Goals for pain management were similar in both groups: visual analogue score < 40/100 in communicating patients or behavioral pain scale of 3–4 in non-communicating patients [20]. Additional details are available in Additional file 1. The research protocols and analysis plans are available in Additional file 2; the CONSORT checklist is provided in Additional file 3.

### Outcomes

The primary endpoint was the number of ventilator-free days from randomization to day 30, as defined as the number of days from randomization to day 30 after randomization during which a patient was able to breathe without invasive assistance. Patients who had died by day 30 were considered to have zero ventilator-free days. Although unusual in acute pancreatitis research, ventilator-free days are frequently used in critical care trials as it can reflect the need for intubation and the duration of invasive mechanical ventilation when needed, while accounting for death as a competing event [17, 18].

Predefined secondary endpoints included: the incidence of various complications at day 30 (including death, sepsis, organ failure, and abdominal complications); the duration of mechanical ventilation (invasive and noninvasive); symptoms of intolerance to enteral feeding; effectiveness of pain management, biological markers of inflammatory response, lung injury, and renal failure; and duration of epidural analgesia therapy. Cost analysis was unfortunately unavailable for this report.

### Data collection and endpoint assessment

Anonymized study data were collected prospectively using REDCap electronic data capture tools hosted at Clermont-Ferrand University Hospital [21]. For each patient, past medical history and baseline demographics, pre-randomization status, clinico-biological variables (daily from randomization through day 7, on day 15, and on day 30), and intercurrent events or complications were recorded. Patients were followed-up until day 30 after randomization. Assessors of clinico-biological outcomes and statistical analyses were masked as to the randomization group. In some centers, blood and urine samples were collected on the day of randomization, on day 2, and on day 7 after randomization for blinded measurements of biomarkers of inflammation, lung injury, and acute kidney injury.

### Sample size

Assuming a mean (± SD) number of 13 ± 15 ventilator-free days in the usual care group [3, 16], a sample size of 148 patients was determined to provide the trial with a power of 80% to detect an absolute between-group difference of 7 ± 15 ventilator-free days at day 30 after randomization with a two-sided type-I error rate of 0.05 [3].

### Statistical analysis

Analyses were performed in the intention-to-treat population, i.e., all patients randomized except those who had withdrawn consent or did not retrospectively meet inclusion criteria. For the primary analysis, we used a Mann–Whitney U test and computed effect-sizes or absolute median differences with 95% confidence intervals (CIs). Given that a high proportion of patients never required intubation (i.e., had 30 ventilator-free days), we performed post-hoc zero-inflated negative binomial regression to estimate the odds ratio for having 30 ventilator-free days and the incident rate ratio for the number of ventilator-free days (when not equal to 30), before and after adjustment for the randomization-stratification variables, including site as random effect.

Analyses of the primary outcome were also performed in the per-protocol population, as defined by all randomized patients, except for patients who withdrew their consent or did not meet the inclusion criteria, including those assigned to the intervention group who received epidural analgesia for less than 72 h. Post-hoc subgroup analyses of the primary outcome based on potential risk factors of worse outcome of acute pancreatitis were performed (including randomization-stratification variables, the need for vasopressor support or intubation at baseline, the presence of sepsis or peripancreatic necrosis at baseline, age, and serum C-reactive protein at baseline) using unadjusted zero-inflated negative binomial regression and testing for heterogeneity between subgroups in the number of ventilator-free days by fitting an interaction between treatment and subgroup.

Secondary outcomes were analyzed as described in the statistical analysis plan (see Additional file 2). No correction for multiple testing was applied for analysis of secondary outcomes or subgroup analysis. Two-sided p-values of less than 0.05 were considered statistically significant. Analyses were performed with Stata software version 15 (StataCorp, College Station, TX, USA) and R version 4.0.5 (R Foundation for Statistical Computing, Vienna, Austria).

## Results

### Enrollment and randomization

**Fig. 1:**
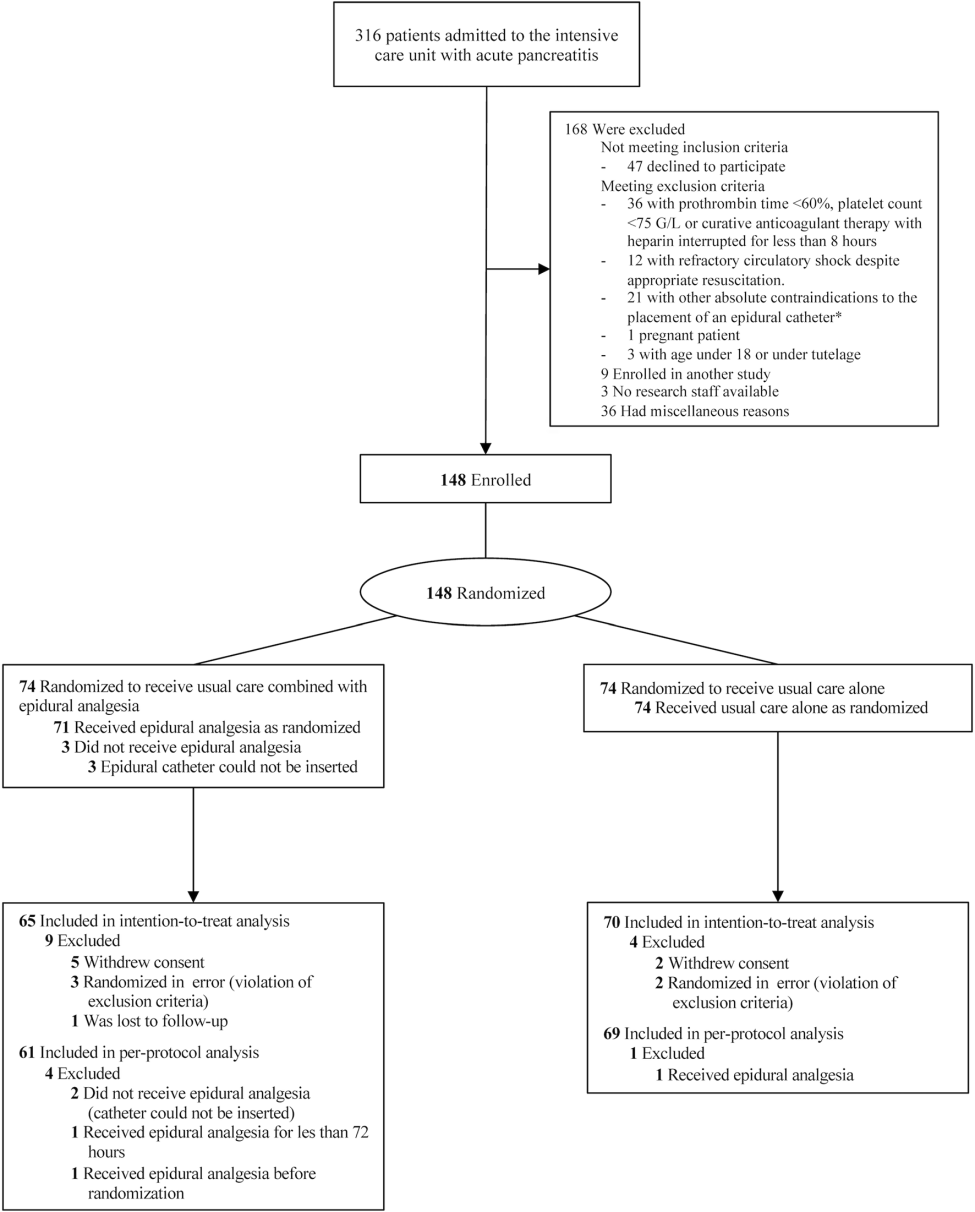
CONSORT patient flowchart. * Reasons for exclusion: local infection, active central nervous system infection, history of back surgery associated with a dural space procedure, suspected, or confirmed intracranial hypertension

From June 2014 through January 2019, a total of 316 patients were assessed for eligibility (Fig. 1 and Additional file 1: Fig. S1). A total of 148 patients (47%) were enrolled and randomly assigned to epidural analgesia and usual care (74 patients) or usual care alone (74 patients). A total of 65 patients in the epidural analgesia and usual care group and 70 in the usual care group were included in the intention-to-treat analysis. Baseline characteristics are presented in Table 1 and in Table S1 (see Additional file 1). 13 patients (20%) assigned to the intervention group and 14 patients (20%) assigned to the control group were under invasive mechanical ventilation at the time of randomization and there was no significant between-group difference in ventilation settings (Additional file 1: Table S2).

**Table 1:** Characteristics of the patients at baseline.*

Characteristic	Epidural analgesia and usual care	Usual care alone	Absolute standardized difference (95% CI)
	(n = 65)	(n = 70)	
Male sex—no. (%)	42 (65)	53 (76)	0.24
Age—yr	58 ± 16	57 ± 18	0.06
Body-mass index—kg/m^2^	28 ± 5	27 ± 5	0.12
Past or current medical history—no. (%)			
Arterial hypertension	30 (46)	32 (46)	0.01
Type 1 diabetes	0 (0)	2 (3)	0.24
Type 2 diabetes	9 (14)	10 (14)	0.02
Chronic heart failure	4 (6)	2 (3)	0.15
Coronary artery disease	8 (12)	6 (9)	0.12
Chronic respiratory disease	4 (6)	2 (3)	0.16
Chronic renal disease	1 (2)	1 (1)	0.01
Solid tumor or hematological cancer			
Evolutive	4 (6)	1 (1)	0.25
In remission	2 (3)	4 (6)	0.13
Stroke	1 (2)	2 (3)	0.09
Other	27 (42)	27 (39)	0.07
Current habits			
Current alcohol abuse—no. (%)	24 (37)	25 (36)	0.01
Current smoker—no. (%)	18 (28)	18 (26)	0.04
McCabe—no. (%)†			0.43
Category 1	41 (84)	56 (92)	
Category 2	7 (14)	3 (5)	
Category 3	1 (2)	2 (3)	
Cause of pancreatitis—no. (%)			
Gallstones	26 (40)	25 (36)	0.09
Alcohol abuse	20 (31)	23 (33)	0.04
Post-ERCP	3 (5)	4 (6)	0.05
Drugs	2 (3)	3 (4)	0.06
Hypertriglyceridemia	5 (8)	8 (11)	0.13
Unknown	12 (18)	8 (11)	0.2
Other	3 (5)	3 (4)	0.02
Duration from first symptoms to randomization			0.05
Median—day	2	2	
Interquartile range — day	1–4	1–5	
≥ 48 h — no. (%)	37 (57)	38 (54)	
Disease severity			
Ranson score	2.8 ± 0.2	2.7 ± 0.2	0.08
APACHE II score	18.7 ± 6.2	17.7 ± 7.4	0.14
SOFA score	4.8 ± 3.6	4.4 ± 4.2	0.11
Respiratory	1.4 ± 1.3	1.3 ± 1.2	0.08
Cardiovascular	1.5 ± 1.8	0.9 ± 1.5	0.38
Liver	0.7 ± 0.9	0.6 ± 0.8	0.07
Coagulation	0.4 ± 0.8	0.4 ± 0.9	0.04
Central Nervous System	0.2 ± 0.5	0.4 ± 0.9	0.3
Renal	0.7 ± 1.0	0.8 ± 1.2	0.11
Serum C-reactive protein—mg/L	235 ± 139	224 ± 153	0.08
Septicemia—no. (%)	1 (2)	4 (6)	0.09
SIRS—no. (%)‡	57 (88)	62 (89)	0.03
Respiratory status—no. (%)			0.02
Spontaneous breathing	49 (75)	53 (76)	
High-flow oxygen therapy	6 (12)	2 (4)	
Non-invasive ventilation	3 (5)	3 (4)	
Invasive ventilation	13 (20)	14 (20)	
Cardiovascular status			
Need for vasopressor or inotropic support — no. (%)	25 (38)	13 (19)	0.45
Norepinephrine	25 (38)	13 (19)	
Dobutamine	1 (2)	1 (1)	
Dopamine	1 (2)	0 (0)	
Milrinone	1 (2)	0 (0)	
Organ failure—no. (%)**			0.12
Absent	15 (23)	16 (23)	
Moderate	31 (48)	37 (53)	
Severe	19 (23)	17 (24)	
Isolated organ failure	35 (54)	31 (44)	0.19
Multiple organ failure	19 (29)	24 (34)	0.11
CT-scan performed—no. (%)	34 (52)	41 (59)	0.11
Extent of pancreas necrosis			0.26
None—no. (%)	11 (41)	13 (34)	
< 30%—no. (%)	7 (26)	8 (21)	
30–50%—no. (%)	5 (19)	8 (21)	
> 50%—no. (%)	4 (15)	9 (24)	
Peripancreatic necrosis	24 (73)	24 (60)	0.27
Infected necrosis	2 (8)	2 (8)	0
Vascular thrombosis—no. (%)	2 (3)	5 (7)	0.19
Mesenteric venous thrombosis—no. (% of patients with thrombosis)	2 (100)	2 (40)	

^*^Plus–minus values are means ± standard deviations. There were no significant between-group differences at baseline

^†^The McCabe score is a marker of co-morbidity and severity of underlying diseases, as divided into three categories (category 1: nonfatal disease such as diabetes, genito-urinary, gastro-intestinal or obstetrical conditions; category 2: ultimately fatal disease, *i.e.* diseases estimated to become fatal within 4 years, such as aplastic anemia, metastatic carcinomas, cirrhosis or chronic renal disease; category 3: rapidly fatal disease, such as acute leukemia, plastic relapse of chronic leukemia) *(JAMA Internal Medicine 1962;110:847–855)*

^‡^The systemic inflammatory response syndrome (SIRS) was diagnosed with the use of the Consensus Conference criteria of the American College of Chest Physicians–Society of Critical Care Medicine *(Crit Care Med. 1992;20:864–74)*

^**^Organ failure was defined based on the modified Marshall score as absent (score of 0), moderate (score of 1–2 for at least one organ function) or severe (score of 3–4 for at least one organ function). The modified Marshall score evaluates the respiratory, renal, and cardiovascular functions, with higher scores indicating more severe disease, as proposed in the revised Atlanta classification of acute pancreatitis *(Gut. 2013;62:102–11)*. Multiple organ failure was defined as failure of two or more organs on the same day

CI: confidence interval; ERCP: endoscopic retrograde cholangiopancreatography; APACHE II: acute physiology and chronic health evaluation II; SOFA: sequential organ failure assessment; SIRS: systemic inflammatory response syndrome; CT: computed tomography

Details on epidural analgesia and co-interventions over the first 7 days after randomization are available in Additional file 1: Fig. 2 and Tables 3–4. The median duration of epidural analgesia was 6 days (interquartile range, 4 to 8) and no severe complication potentially attributable to epidural analgesia (epidural hematoma or infection) was reported (Additional file 1: Table S5). All clinico-biological variables recorded through day 7 after randomization are provided in  Additional file 1: Tables S6–S17.

### Outcomes

#### Primary outcome

**Fig. 2:**
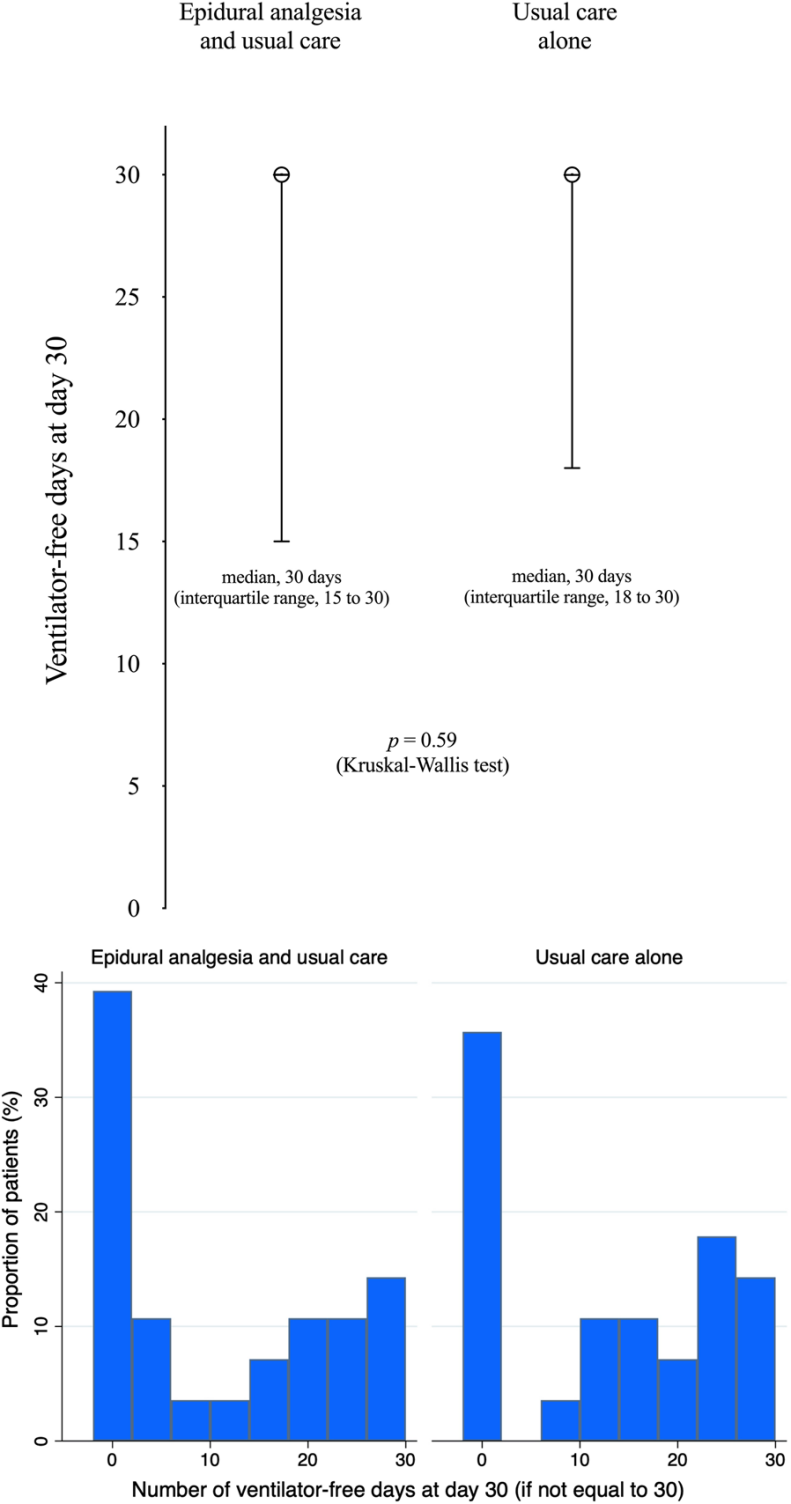
Ventilator-free days from randomization to day 30. *Top:* median values and interquartile ranges. *Bottom*: distribution of ventilator-free days when not equal to 30

The number of ventilator-free days did not differ significantly between the epidural analgesia and usual care group and the usual care group, with a median duration of 30 days (interquartile range 15–30) and 30 days (interquartile range 18–30), respectively, for a median absolute difference of − 0.0 days (95% CI − 3.3 to 3.3; *p* = 0.59) (Fig. 2 and Table 2). Forty-two patients (60%) assigned to the control group and 37 patients (57%) assigned to the intervention group never required intubation, and zero-inflated negative binomial regression found no between-group differences in both the odds ratio for having 30 ventilator-free days (i.e., never requiring intubation) and the duration of invasive mechanical ventilation in patients with ventilator-free days not equal to 30 (i.e., those requiring invasive ventilation) (Additional file 1: Table S18). Similar results were found after multivariable adjustments and in the per-protocol population (Additional file 1: Tables S18–S19).

**Table 2:** Primary and secondary endpoints, according to the intention-to-treat unadjusted analysis

Outcome	Epidural analgesia and usual care	Usual care alone	Absolute median difference	*p* value*	Relative risk or
	(n = 65)	(n = 70)	(95% CI)		regression coefficient (95% CI)
Primary endpoint					
Ventilator-free days at day 30 — median [IQR]	30 [15–30]	30 [18–30]	0.00 (− 3.33; 3.33)	0.59	− 0.15 (− 0.58; 0.27)‡
Secondary endpoints					
Death at day 30 — no. (%)	6 (9)	10 (14)	0.05 (− 0.06; 0.16)	0.36	0.65 (0.25; 1.68)§
Organ failure at day 30 — no. (%)					
Development of ARDS	15 (23)	10 (14)	− 0.09 (− 0.22; 0.05)	0.2	1.59 (0.77; 3.29)§
Need for renal replacement therapy	12 (18)	12 (17)	− 0.01 (− 0.14; 0.12)	0.84	1.08 (0.52; 2.22)§
Need for vasopressor support	29 (45)	28 (40)	− 0.05 (− 0.21; 0.12)	0.59	1.12 (0.75; 1.65)§
New-onset organ failure	15 (23)	17 (24)	0.01 (− 0.14; 0.15)	0.91	0.97 (0.53; 1.77)§
Duration of invasive mechanical ventilation (days)—median [IQR]	14 [5–28]	6 [2–13]	8.00 (− 0.86; 16.86)	0.02	0.56 (0.11; 1.02)‡
Duration of non-invasive mechanical ventilation (days)—median [IQR]	0 [0–2]	0 [0–2]	0.00 (− 0.45; 0.45)	0.84	− 0.06 (− 0.37; 0.26)‡
Sepsis at day 30 — no. (%)	36 (55)	32 (46)	− 0.10 (− 0.26; 0.07)	0.26	1.21 (0.87; 1.70)§
Septic shock at day 30 — no. (%)	23 (35)	23 (33)	− 0.03 (− 0.19; 0.13)	0.76	1.08 (0.67; 1.72)§
Abdominal complications at day 30 — no. (%)					
Abdominal compartment syndrome	5 (8)	6 (9)	0.01 (− 0.08; 0.10)	0.81	0.87 (0.28; 2.72)§
Infected peripancreatic necrosis	10 (15)	9 (13)	− 0.03 (− 0.14; 0.09)	0.67	1.20 (0.52; 1.76)§
Peripancreatic fluid collections	29 (45)	25 (36)	− 0.09 (− 0.25; 0.08)	0.29	1.25 (0.82; 1.89)§
Infected peripancreatic fluid collections	40 (74)	35 (83)	0.09 (− 0.07; 0.25)	0.28	0.89 (0.72; 1.09)§
Walled-off pancreatic necrosis (persistent)	17 (30)	15 (24)	− 0.06 (− 0.22; 0.10)	0.49	1.23 (0.68; 2.23)§
Need for necrosectomy	7 (11)	7 (10)	− 0.01 (− 0.11; 0.10)	0.88	1.08 (0.40; 2.90)§
Requiring surgical drainage	2 (3)	1 (1)	− 0.02 (− 0.07; 0.03)	0.52	2.15 (0.20; 23.19)§
Need for multiple necrosectomies	5 (8)	3 (4)	− 0.29 (− 0.78; 1.05)	0.28	1.67 (0.63; 4.42)§
Intolerance to enteral feeding during the first week after randomization—no. (%)					
Nausea	13 (20)	9 (13)	− 0.07 (− 0.20; 0.30)	0.26	1.56 (0.71; 3.39)§
Vomiting	13 (20)	13 (19)	− 0.01 (0.10; 0.30)	0.83	1.08 (0.54; 2.15)§
Ileus (requiring pro-kinetic therapy)	34 (52)	33 (47)	− 0.05 (− 0.22; 0.12)	0.55	1.11 (0.79; 1.56)§
Diarrhea	16 (25)	17 (24)	− 0.00 (− 0.15; 0.14)	0.96	1.01 (0.16; 1.83)§
Visual analogue score for pain ≥ 40/100 during the first week after randomization (in communicating patients) — no. (%)					
At rest	31 (55)	34 (55)	− 0.01 (− 0.18; 0.17)	0.96	1.01 (0.73; 1.40)§
During nursing procedures	32 (59)	42 (69)	0.10 (− 0.08; 0.27)	0.28	0.86 (0.65; 1.14)§
Behavioral pain scale > 4 during the first week after randomization (in non-communicating patients) — no. (%)					
At rest	3 (14)	6 (33)	0.19 (− 0.07; 0.45)	0.16	0.43 (0.11; 1.60)§
During nursing procedures	5 (25)	7 (39)	0.14 (− 0.16; 0.43)	0.36	0.64 (0.24; 1.74)§
Biomarker levels during the first week after randomization — median [IQR]					
Plasma interleukin-6 (pg/mL)					
Day 0	232 [94–524]	191 [67–439]	53 (− 98; 204)	0.49	0.31 (− 0.29; 0.91)‡
	(n = 52)	(n = 53)			
Day 2	131 [75–266]	113 [61–351]	15 (− 76; 106)	0.68	0.06 (− 0.48; 0.60)‡
	(n = 52)	(n = 48)			
Day 7	57 [26–126]	41 [17–161]	15 (− 34; 64)	0.69	− 0.01 (− 0.73; 0.71)‡
	(n = 42)	(n = 32)			
Plasma sRAGE (pg/mL)					
Day 0	451 [282–651]	465 [342–707]	− 12 (− 144; 120)	0.61	− 0.07 (− 0.37; 0.23)‡
	(n = 52)	(n = 53)			
Day 2	538 [335–740]	614 [394–933]	− 84 (− 278; 110)	0.26	− 0.15 (− 0.41; 0.12)‡
	(n = 52)	(n = 48)			
Day 7	642 [408–827]	594 [352–914]	40 (171; 251)	0.96	− 0.04 (− 0.36; 0.27)‡
	(n = 42)	(n = 32)			
Plasma NGAL (ng/mL)					
Day 0	218 [107–478]	184 [86–451]	27 (− 105; 159)	0.46	0.12 (− 0.39; 0.62)‡
	(n = 51)	(n = 48)			
Day 2	246 [127–389]	204 [120–353]	39 (− 53; 131)	0.58	0.10 (− 0.30; 0.49)‡
	(n = 49)	(n = 46)			
Day 7	187 [113–496]	184 [108–532]	1 (− 193; 195)	0.87	0.06 (− 0.41; 0.53)‡
	(n = 40)	(n = 30)			
Urine TIMP-2*IGFBP-7 (Nephrocheck score)					
Day 0	0.44 [0.21–1.44]	0.27 [0.07–0.85] (n = 20)	0.09 (− 0.62; 0.80)	0.22	0.25 (− 0.21; 0.71)‡
	(n = 29)	0.15 [0.11–0.58] (n = 20)			
Day 2	0.11 [0.04–0.61]	0.15 [0.08–0.32] (n = 20)	− 0.04 (− 0.35; 0.27)	0.55	0.02 (− 0.20; 0.23)‡
	(n = 29)				
Day 7	0.12 [0.04–0.34]		− 0.03 (− 0.20; 0.14)	0.68	− 0.01 (− 0.22; 0.19)‡
	(n = 29)				

^*^P values were calculated using the χ^2^ or Fisher exact test, as appropriate, for categorical data and the unpaired t test or Mann–Whitney U test for continuous data

^‡^Regression coefficient, as expressed for each one-log increase in the continuous dependent variable

^§^Relative risk for binary dependent variables

CI: confidence interval; IQR: interquartile range; ARDS: acute respiratory distress syndrome; sRAGE: soluble receptor for advanced glycation end-products. NGAL: neutrophil gelatinase-associated lipocalin. TIMP-2: tissue inhibitor of metalloproteinases-2; IGFBP7: insulin-like growth factor-binding protein

**Fig. 3:**
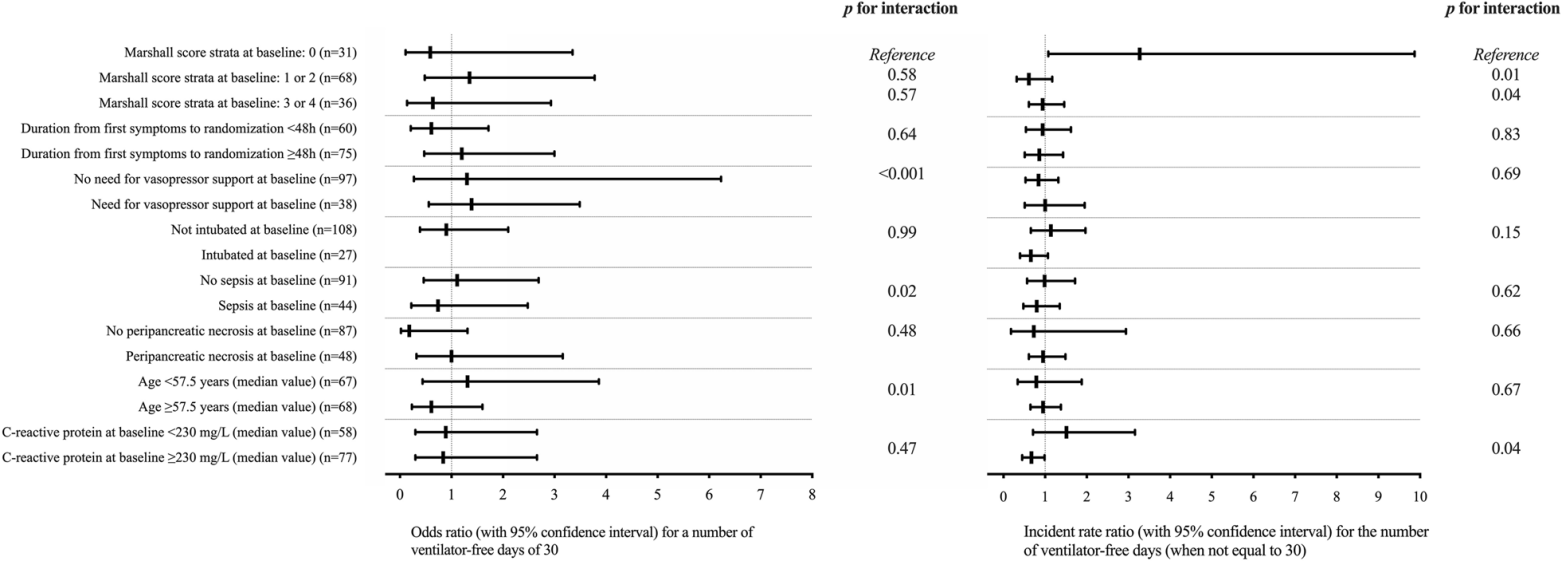
Post-hoc subgroup analysis of the primary endpoint of ventilator-free days from randomization to day 30. The odds ratio for having 30 ventilator-free days and the incident rate ratio for the number of ventilator-free days (when not equal to 30) were computed using zero-inflated negative binomial regression

The number of ventilator-free days did not differ significantly between the two groups in most subgroup analyses (Fig. 3). However, the incident rate ratios for the number of ventilator-free days when not equal to 30 were 3.27 (95% CI 1.08–9.87) in patients without organ dysfunction at baseline (i.e., with a Marshall score of zero) and 0.67 (95% CI 0.45–0.99) in patients with higher baseline levels of serum C-reactive protein. A post-hoc sensitivity analysis found fewer ventilator-free days in patients from the intervention group with a higher SOFA score at baseline (Additional file 1: Table S20).

#### Secondary outcomes

By day 30, deaths were reported for 6 of 65 patients (9%) and 10 of 70 patients (14%) in the intervention and control groups, respectively (unadjusted relative risk, 0.65; 95% CI 0.25–1.68; *p* = 0.36) (Table 2). Invasive ventilation was needed within 30 days from randomization in a total of 27 (42%) and 29 patients (41%) from the intervention and control groups, respectively; in these patients, the median duration of invasive ventilation was increased in the intervention group (14 days, interquartile range 5–28), compared to the control group (6 days, interquartile range 2–13) (unadjusted regression coefficient per one-log increment in duration of ventilation, 0.56; 95% CI 0.11–0.55; *p* = 0.02) (Table 2). Despite similar analgesia scores in both groups, patients assigned to epidural analgesia had decreased opioid requirements from randomization through day 7 (Additional file 1: Fig. S3 and Table S8). The frequency of abdominal and extra-abdominal complications was high but did not differ between the groups (Table 2 and Additional file 1: Tables S6–S13). There was no obvious between-group difference in surrogates of intolerance to enteral feeding (nausea, vomiting, ileus requiring pro-kinetic therapy, and/or diarrhea) or in caloric intakes through the enteral route during the first week after randomization (Additional file 1: Table S14).

Attenuations of acute systemic inflammation, lung injury, and kidney injury were hypothesized to be part of the beneficial effect of epidural analgesia in acute pancreatitis. However, such effects did not occur (Table 2 and Additional file 1: Fig. S4).

## Discussion

In this trial, there was no difference in ventilator-free days at day 30 between intensive care unit patients with acute pancreatitis receiving thoracic epidural analgesia combined with usual care and those receiving usual care alone. There was no between-group difference in the incidence of acute pancreatitis-related complications, and patients who received epidural analgesia were less likely to receive opioids despite similar between-group analgesia scores. However, the duration of invasive ventilation was higher in the intervention group than in the control group in patients intubated within 30 days from randomization.

Our trial did not confirm the hypothesized benefits of epidural analgesia. In animal models of acute pancreatitis, epidural analgesia improved arterial oxygenation, decreased systemic inflammation and liver injury, increased gut barrier function, and improved splanchnic, pancreatic, and renal perfusion [22–27]. There was an important mortality reduction with epidural analgesia in two studies in rats and pigs [23, 26], as well as in one retrospective clinical study [15]. However, two small, randomized trials including patients with severe acute pancreatitis found no significant differences in mortality or other clinical outcomes between patients treated with epidural analgesia and those who were not [16, 28].

Several hypotheses could explain the differences between the current results and our initial assumptions. First, a major limitation is that our population does not correspond to the population in which the trial hypothesis was generated, since the observed number of ventilator-free days at day 30 was higher than initially assumed. Therefore, no definitive conclusion can be drawn from the current study on the efficacy of epidural analgesia in improving clinical outcomes in a population of patients who would have higher rates of intubation requirements. The less-frequent need for invasive ventilation at day 30 in our trial (41–42%), as compared to that in the study from Jung et al. (58%) [3], could be explained by a more frequent use of non-invasive ventilation at day 30 (37–39% versus 15%, respectively). In addition, ventilator-free days are more frequently used as secondary endpoints than as primary endpoints in acute pancreatitis trials [4, 29] and their skewed distribution makes their analysis difficult with usual statistical tests, which are major limitations [17, 18]. However, post-hoc zero-inflated negative binomial regression confirmed no between-group differences in both the risk of never requiring intubation and the duration of invasive mechanical ventilation if intubated. Second, the pragmatic design of the trial included wide eligibility criteria, possibly allowing the inclusion of patients with mild acute pancreatitis and potential selection bias. We did not use scoring systems for severity prediction, which are only moderately accurate [30], but rather stratified randomization on the severity of organ dysfunction using the modified Marshall scoring system. However, the mortality rate was higher in our cohort (overall mortality rate, 11.9%) than in the trial from Sadowski et al. (0%) [16], but lower than in a multicenter retrospective study including critically ill patients (21%) [15]. The rates of complications and organ-supportive measures were high, in line with those in patients with predicted severe acute pancreatitis [31], thus suggesting that the need for intubation is not the sole marker of severity in acute pancreatitis. Indeed, baseline severity scores, such as the Acute Physiology and Chronic Health Evaluation II [32], were higher in our trial than in some recent studies [3, 15, 31, 33] and plasma interleukin-6 levels were higher than previously reported in acute pancreatitis [34]. Although epidural bupivacaine decreased plasma interleukin-6 in a rat model of acute pancreatitis [23], there was no between-group difference in plasma interleukin-6 over time in our trial. Whether the severity of systemic inflammation may affect the effect of epidural analgesia on interleukin-6 levels, or whether such an attenuation of the systemic inflammatory response may not be observed with ropivacaine, is unknown [35]. Also, the association of at least one organ dysfunction or high serum C-reactive protein levels at baseline with fewer ventilator-free days in subgroup analysis suggests that selecting which patients might benefit from epidural analgesia should be further investigated.

Secondary analyses found that the duration of mechanical ventilation was higher in patients who received epidural analgesia than in those who did not. It is possible that the intervention directly affected weaning from mechanical ventilation and may be harmful in our trial population, although potential mechanisms remain unreported to date. In addition, epidural analgesia had no effect on biomarkers of lung or kidney injury over time and it reduced opioid requirements while providing efficient analgesia. Whether a reduction in opioid requirements could benefit a population of patients with higher intubation rates warrants further investigation. Although our sample size is limited and our study underpowered to bring definitive conclusions, this is concordant with previous findings supporting the potential safety of epidural analgesia when administered for multiple days in intensive care unit patients, including those with sepsis or under sedation [13, 14, 16].

This trial has limitations. It did not have detailed protocols addressing each single aspect of the management of patients with acute pancreatitis, such as fluid therapy or the initiation, route of administration, and dose of enteral feeding [6]. Although the median durations from first symptoms to randomization were 2 days in both groups, 57% and 54% of patients from the intervention and control groups, respectively, had a duration from pain onset to randomization longer than 48 h, which may have influenced clinical management and outcomes [36]. Despite a long study period, our sample size was rather small and imbalanced between groups, with 9% of the enrolled population unavailable for analysis due to violations of exclusion criteria or consent withdrawals, as per French law. Our sample size estimation, based on an expected absolute increase in ventilator-free days of more than 50%, was over-optimistic, further decreasing statistical power and questioning the extrapolability of our findings to other selected populations. The high number of secondary endpoints and multiple testing without adjustment for multiplicity are also limitations; such exploratory results should be cautiously interpreted as hypothesis-generating. In addition, epidural analgesia is restricted to anesthesiologists or anesthesiologists-intensivists and requires specific training and close monitoring to avoid complications such as epidural hematoma or infection.

This study also has strengths. It is the first multicenter randomized trial of epidural analgesia in acute pancreatitis. Despite its open-label design, final assessors of clinico-biological data, statistical analyses, and outcome assessment remained masked to the treatment group. However, many questions remain on the timing and duration of epidural analgesia [13, 37], the level of epidural catheter placement [38], and the choice of local anesthetic or its combination with opioids [39].

## Conclusion

In a population of intensive care unit adults with acute pancreatitis and low requirement for intubation, this first multicenter randomized trial did not show the hypothesized benefit of epidural analgesia in addition to usual care. Therefore, no definitive conclusion can be drawn from the current results on the efficacy of epidural analgesia in improving clinical outcomes in a population of patients who would have higher intubation rates. Although epidural analgesia was efficient in reducing opioid requirements, it was significantly associated with longer duration of invasive ventilation in our cohort. The potential harm of this intervention in critically ill patients requires further investigation.

## Supplementary Information

**Additional file 1**. List of investigators and additional details.

**Additional file 2**. Research protocols and analysis plans.

**Additional file 3**. CONSORT checklist.

## Data Availability

The research protocols and analysis plans are available in Additional file 2. Deidentified data will be available at time of publication to researchers who provide a methodologically sound and ethically approved proposal, for any purpose of analysis. A data use agreement will be required before the release of participant data and institutional review board approval as appropriate.

## Abbreviations

CI: Confidence interval
CONSORT: Consolidated standards of reporting trials
EPIPAN: Epidural analgesia for acute pancreatitis
REDCap: Research electronic data capture
