# Supplementary material for: Thoracic epidural analgesia in intensive care unit patients with acute pancreatitis: the EPIPAN multicenter randomized controlled trial
Source: Crit Care. 2023 May 31;27:213. doi: 10.1186/s13054-023-04502-w (PMC10230742; doi:10.1186/s13054-023-04502-w)
Supplement: Supplementary file 1 — Additional file 1. List of investigators and additional details. [file 13054_2023_4502_MOESM1_ESM.pdf]

Additional File 1

Thoracic epidural analgesia in intensive care unit patients with acute pancreatitis: the EPIPAN multicenter randomized controlled trial

The protocol and statistical analysis plan are published in *BMJ Open 2017 29;7(5):e015280*.

TABLE OF CONTENT

Additional information

List of investigators in the EPIPAN study group *Page 3*

Study design and oversight *Page 6*

Study procedures *Page 6*

Study outcomes *Page 8*

Patient safety *Page 9*

Statistical analysis *Page 10*

References *Page 12*

Supplementary figures

Supplementary Figure 1. Number of patients enrolled and analyzed in each participating center. *Page 13*

Supplementary Figure 2. Durations of epidural analgesia use in the trial. *Page 14*

Supplementary Figure 3. Opioid requirements during the first week after randomization. *Page 15*

Supplementary Figure 4. Median biomarker levels during the first week after randomization. *Page 16*

Supplementary Tables

Supplementary Table 1. Other characteristics of the patients at baseline. *Page 17*

Supplementary Table 2. Respiratory parameters of the patients at baseline. *Page 19*

Supplementary Table 3. Details on the initiation of epidural analgesia in patients randomized to the intervention group. *Page 20*

Supplementary Table 4. Courses of epidural analgesia during the first seven study days in patients randomized to the intervention group *Page 22*

Supplementary Table 5. Complications potentially attributable to epidural analgesia in patients randomized to the intervention group from randomization to day 30. *Page 23*

Supplementary Table 6. Hemodynamic parameters during the first seven days after randomization. *Page 24*

Supplementary Table 7. Respiratory parameters during the first seven days after randomization. *Page 27*

Supplementary Table 8. Neurological parameters and use of sedatives and analgesics during the first seven days after randomization. *Page 29*

Supplementary Table 9. Renal parameters during the first seven days after randomization. *Page 32*

Supplementary Table 10. Abdominal complications during the first seven days after randomization. *Page 33*

Supplementary Table 11. Septic complications during the first seven days after randomization. *Page 34*

Supplementary Table 12. Criteria for systemic inflammatory response syndrome during the first seven days after randomization. *Page 35*

Supplementary Table 13. Sequential organ failure assessment (SOFA) score during the first seven days after randomization. *Page 36*

Supplementary Table 14. Digestive and nutritional parameters during the first seven days after randomization. *Page 37*

Supplementary Table 15. Use of anticoagulant therapy and antiplatelet drugs during the first seven days after randomization. *Page 39*

Supplementary Table 16. Routine laboratory results during the first seven days after randomization. *Page 40*

Supplementary Table 17. Deaths and their causes of deaths during the first seven days after randomization. *Page 42*

Supplementary Table 18. Analysis of the primary outcome and post-hoc zero-inflated negative binomial regression in the intention-to-treat population. *Page 43*

Supplementary Table 19. Analysis of the primary outcome restricted to the per-protocol population. *Page 44*

Supplementary Table 20. Post-hoc unadjusted sensitivity analysis of the primary outcome and zero-inflated negative binomial regression in the intention-to-treat population, as reported for each quartile of the SOFA score at baseline. *Page 45*

Additional information: list of investigators in the EPIPAN study group

First Name	Last Name	Institution
Elodie	Caumon	CHU Clermont-Ferrand, Clermont-Ferrand, France
Julien	Amat	
Dominique	Morand	
Renaud	Guérin	
Sébastien	Perbet	
Benjamin	Rieu	
Sophie	Cayot	
Christian	Chartier	
Camille	Verlhac	
Christine	Rolhion	
Justine	Bourdier	
Bernard	Cosserant	
Raiko	Blondonnet	
Jean-Baptiste	Joffredo	
Thomas	Costilles	
Damien	Bouvier	
Lise	Bernard	
Jean-Etienne	Bazin	
Alexandra	Genevrier	
Russell	Chabanne	
Thomas	Godet	
Laurence	Roszyk	
Vincent	Sapin	
Emmanuel	Futier	
Bruno	Pereira	
Jean-Michel	Constantin	
Matthieu	Jabaudon	
Lydie	Marie-Anne	Geneva university hospitals, Geneva, Switzerland

Olivier	Windisch	
Raphaël	Giraud	
Claudia Paula	Heidegger	
Leo	Bühler	
Annick	Puchois	Cannes general hospital, Cannes, France
Cyril	Boronad	
Marine	Agullo	
Pierre-Marie	Bertrand	
Boris	Jung	CHU Montpellier, Montpellier, France
Gérald	Chanques	
Cécile	Spirito	
Marion	Monnin	
Albert	Prades	
Moussa	Cisse	
Anne	Verchere	
Claudine	Gniadek	
Fouad	Belafia	
Daniel	Verzilli	
Julie	Carr	
Audrey	De Jong	
Yannael	Coisel	
Jean-Marc	Delay	
Matthieu	Conseil	
Marie	Gonzalez	
Delphine	Rosant	
Samir	Jaber	
Michel	Prevot	CHU Nancy-Brabois, Nancy, France
Philippe	Guerci	
Bernard	Claud	Emile-Roux general hospital, Le Puy-en-Velay, France
François	Brenas	
Lassane	Zanre	

Philippe	Bray	
Hélène	Riera	
Emilie	Gadea-Deschamps	
Achille	Sossou	
Pablo	Massanet	CHU Nîmes, Nîmes, France
Caroline	Boutin	
Saber	Barbar	
David-Paul	De Brauwere	
Serge	Lumbroso	
Amélie	Maurin	
Sophie	Lloret	
Laurent	Muller	
Claire	Roger	
Jean-Yves	Lefrant	
Loubna	Elotmani	
Audrey	Ayral	
Stéphanie	Bulyez	
Suzanne	Renard	Saint Luc university hospital, Université Catholique de Louvain, Brussels, Belgium
Pierre-François	Laterre	
Nadège	Bouskila	Nice Archet 2 university hospital, Nice, France
Pierre-Eric	Danin	
Gaspard	Beaune	Annecy Genevois general hospital, Annecy, France
Magali	Farines-Raffoul	
Marie	Lebouc	
Etienne	Escudier	
Auguste	Dargent	Hospices Civils de Lyon, Lyon, France
Thomas	Crozon	
Julien	Clauzel	
Marinne	Le Core	
Thomas	Rimmelé	
Martin	Bonnassieux	

Additional information: study design and oversight

The EPIPAN trial was a pragmatic, multicenter, randomized, controlled, open-label, and parallel group superiority trial performed in 11 intensive care units at university and non-university medical centers in France, Switzerland, and Belgium. The trial was conducted according to the previously published protocol [1].

Patients were randomly assigned at a 1:1 ratio either to receive epidural analgesia for at least 72 hours and usual care (the intervention group) or usual care alone (the control group). Randomization was performed centrally by local investigators with the use of an electronic web-based system that used permuted-block randomization with a concealed, varying block size (TENALEA, FormsVision BV, the Netherlands). Randomization was stratified according to the center, duration of symptoms (<48 *vs* ≥48 hours from first symptoms to enrollment), and severity of acute pancreatitis as assessed by the modified Marshall scoring system for organ dysfunction (three strata of increasing severity were defined according to the maximum score obtained for at least one of the respiratory, renal, or hemodynamic functions).

All patients or their legal representatives provided written informed consent. The study protocol was approved by the French Ethics Committee (*Comité de Protection des Personnes Sud-Est VI*; approval AU1090) and Medicine Agency (*Agence Nationale de Sécurité du Médicament*; approval 131557A-32), as well as all participating centers. The sponsors were not involved in the design or conduct of the study, the preparation of the manuscript, or the decision to submit it for publication.

Additional information: study procedures

Patients assigned to epidural analgesia and usual care received thoracic epidural analgesia as soon as possible after randomization. An epidural catheter was placed in an intervertebral space between the sixth and ninth thoracic vertebra by a certified anesthesiologist-intensivist or a resident in anesthesiology and intensive care, under the supervision of a certified anesthesiologist-intensivist. A

mixed solution of ropivacaine (2 mg/mL) and sufentanil (0.5 μg/mL) was administered for at least 72 h using a patient-controlled epidural analgesia system with continuous infusion rates set between 5 and 15 mL/h and boli of 3 to 10 mL every 10 minutes at maximum. Nurses were encouraged to administer boli to achieve analgesia goals when the patient was not able to self-administer. Supplemental iterative epidural administrations of clonidine (1 μg/kg) were allowed to achieve analgesia goals. The duration and weaning of epidural analgesia, as well as removal of the epidural catheter, were conducted according to routine protocols from each participating center.

Patients assigned to ~~standard~~ usual care alone did not receive epidural analgesia. In the two study groups, ~~standard~~ usual care was based on recommendations from consensual guidelines on the management of acute pancreatitis: resuscitation measures to correct hypovolemia, maintenance of electrolyte balance, correction of acidosis, early enteral nutrition when possible, and early diagnosis and supportive treatment of complications [2–4]. In particular, criteria for intubation were based on current recommendations and included any of the following major clinical events: respiratory or cardiac arrest, respiratory pauses with loss of consciousness or gasping for air, massive aspiration, persistent inability to clear respiratory secretions, heart rate <50/min with loss of alertness, and severe haemodynamic instability without response to fluid and vasoactive drugs. When invasive mechanical ventilation is needed, the use of a low-tidal-volume protective ventilatory strategy and recommendations on weaning from mechanical ventilation are strongly encouraged at each participating center [1,5,6]. The goals for pain management were the same in both groups: regular evaluation of pain every 4 h at least; targeted visual analogue score <40/100 (in communicating patients) and behavioral pain scale of 3–4 (in non-communicating patients); and a stepped, multimodal approach combining opioid and non-opioid drugs administered through the oral, enteral, and/or intravenous routes as per treating clinicians.

Additional information: study outcomes

Primary outcome measure

The primary outcome variable was the number of ventilator-free days from randomization to day 30, as defined as the number of days from randomization to day 30 after randomization during which a patient was able to breathe without invasive assistance. Patients who died by day 30 were considered to have zero ventilator-free days. A period of invasive mechanical ventilation lasting less than 24 hours and for the purpose of a surgical procedure did not count against the VFD calculation.

Secondary outcome measures

Predefined secondary endpoints included:

- The incidence of general and regional complications from randomization through day 30: death, sepsis, septic shock, organ failure (development of acute respiratory distress syndrome, need for renal replacement therapy, need for vasopressor support, new-onset organ failure), and abdominal complications (abdominal compartment syndrome, infected peripancreatic necrosis, peripancreatic fluid collections, infected peripancreatic fluid collections, persistent walled-off pancreatic necrosis, need for necrosectomy);
- The duration of invasive and non-invasive mechanical ventilation from randomization through day 30;
- Symptoms of intolerance to enteral feeding (defined as nausea, vomiting, or ileus requiring pro-kinetic therapy) from randomization through day 7;
- Effectiveness of pain management from randomization through day 7: visual analogue score in communicating patients or behavioral pain scale in non-communicating patients, opioid requirements;
- Duration of epidural analgesia therapy from randomization through day 30;

- Biological inflammatory and renal response (biomarker analysis) before randomization and on days 2 and 7 after randomization:
    - plasma interleukin-6, a proinflammatory cytokine (duplicate measurement at each timepoint using ELISA; R&D Systems, Minneapolis, Minnesota, USA),
    - plasma soluble receptor for advanced glycation end-products (sRAGE), a marker of lung alveolar epithelial injury (duplicate measurement at each timepoint using ELISA; R&D Systems, Minneapolis, Minnesota, USA),
    - plasma neutrophil gelatinase-associated lipocalin (NGAL), a biomarker of acute kidney injury (duplicate measurement at each timepoint using the *Triage Metre*; Alere, San Diego, California, USA),
    - urine TIMP-2*IGFBP-7, a biomarker of high risk of acute kidney injury (duplicate measurement at each timepoint using the NEPHROCHECK Test; System, Biomérieux, Marcy-l'Étoile, France);
- Health care costs related to the management of ICU patients with acute pancreatitis from randomization through day 30; however, cost analysis is currently unavailable and is not reported in this manuscript.

Additional information: patient safety

An independent data and safety monitoring committee oversaw the trial and reviewed the planned interim analysis after 74 patients had been enrolled. Adverse events were listed and presented to the committee in an unblinded fashion.

Additional information: statistical analysis

Assuming a mean (±standard deviation) number of 13±15 ventilator-free days in the standard care group [7,8], a sample size of 148 patients was determined to provide the trial with a power of 80% to detect an absolute between-group difference of 7±15 ventilator-free days at day 30 after randomization with a two-sided type-I error rate of 0.05 [7].

Analyses were performed in the intention-to-treat population, which included all the patients who had undergone randomization except for those who had withdrawn consent for the use of their data or did not meet the inclusion criteria retrospectively.

For the primary analysis, we used a Mann-Whitney U test and computed effect-sizes or absolute median differences with 95% confidence intervals (CIs).

Given the distribution of the primary outcome measure due to a higher proportion of patients who never required intubation than hypothesized (i.e., with 30 ventilator-free days), we performed zero-inflated negative binomial regression to estimate the odds ratio for having 30 ventilator-free days and the incident rate ratio for the number of ventilator-free days (when not equal to 30) before and after adjustment for the randomization-stratification variables (including site as random effect). Analyses of the primary endpoint were also performed in the per-protocol population, as defined by all randomized patients, except for patients who withdrew their consent or did not meet the inclusion criteria, including those assigned to the intervention group who received epidural analgesia for less than 72 h.

Post-hoc subgroup analyses of the primary outcome based on potential risk factors of worse outcome of acute pancreatitis were performed (including randomization-stratification variables, the need for vasopressor support or intubation at baseline, the presence of sepsis or peripancreatic necrosis at baseline, age, and serum C-reactive protein at baseline) using unadjusted zero-inflated negative binomial regression and testing for heterogeneity between subgroups in the number of ventilator-free days by fitting an interaction between treatment and subgroup.

Secondary endpoints were analyzed as described in the statistical analysis plan (Additional File 2). Statistical significance was indicated by a *p*-value of less than 0.05 and was determined using a two-sided hypothesis test. No correction for multiple testing was applied for analysis of secondary outcomes or subgroup analysis. Analyses were performed with Stata software version 15 (StataCorp, College Station, TX) and R version 4.0.5 (R Foundation for Statistical Computing, Vienna, Austria).

Additional information: references

1. Bulyez S, Pereira B, Caumon E, Imhoff E, Roszyk L, Bernard L, et al. Epidural analgesia in critically ill patients with acute pancreatitis: the multicentre randomised controlled EPIPAN study protocol. BMJ Open. British Medical Journal Publishing Group; 2017;7:e015280.

2. Banks PA, Bollen TL, Dervenis C, Gooszen HG, Johnson CD, Sarr MG, et al. Classification of acute pancreatitis--2012: revision of the Atlanta classification and definitions by international consensus. Gut. 2013;62:102–11.

3. Tenner S, Baillie J, DeWitt J, Vege SS, American College of Gastroenterology. American College of Gastroenterology guideline: management of acute pancreatitis. Am J Gastroenterol. 2013;108:1400–15; 1416.

4. Working Group IAP/APA Acute Pancreatitis Guidelines. IAP/APA evidence-based guidelines for the management of acute pancreatitis. Pancreatology. 2013;13:e1–15.

5. Evans L, Rhodes A, Alhazzani W, Antonelli M, Coopersmith CM, French C, et al. Surviving sepsis campaign: international guidelines for management of sepsis and septic shock 2021. Intensive Care Med [Internet]. 2021; Available from: http://dx.doi.org/10.1007/s00134-021-06506-y

6. Boles J-M, Bion J, Connors A, Herridge M, Marsh B, Melot C, et al. Weaning from mechanical ventilation. Eur Respir J. 2007;29:1033–56.

7. Jung B, Carr J, Chanques G, Cisse M, Perrigault P-F, Savey A, et al. [Severe and acute pancreatitis admitted in intensive care: a prospective epidemiological multiple centre study using CClin network database]. Ann Fr Anesth Reanim. 2011;30:105–12.

8. Sadowski SM, Andres A, Morel P, Schiffer E, Frossard J-L, Platon A, et al. Epidural anesthesia improves pancreatic perfusion and decreases the severity of acute pancreatitis. World J Gastroenterol. 2015;21:12448–56.

Supplementary figures

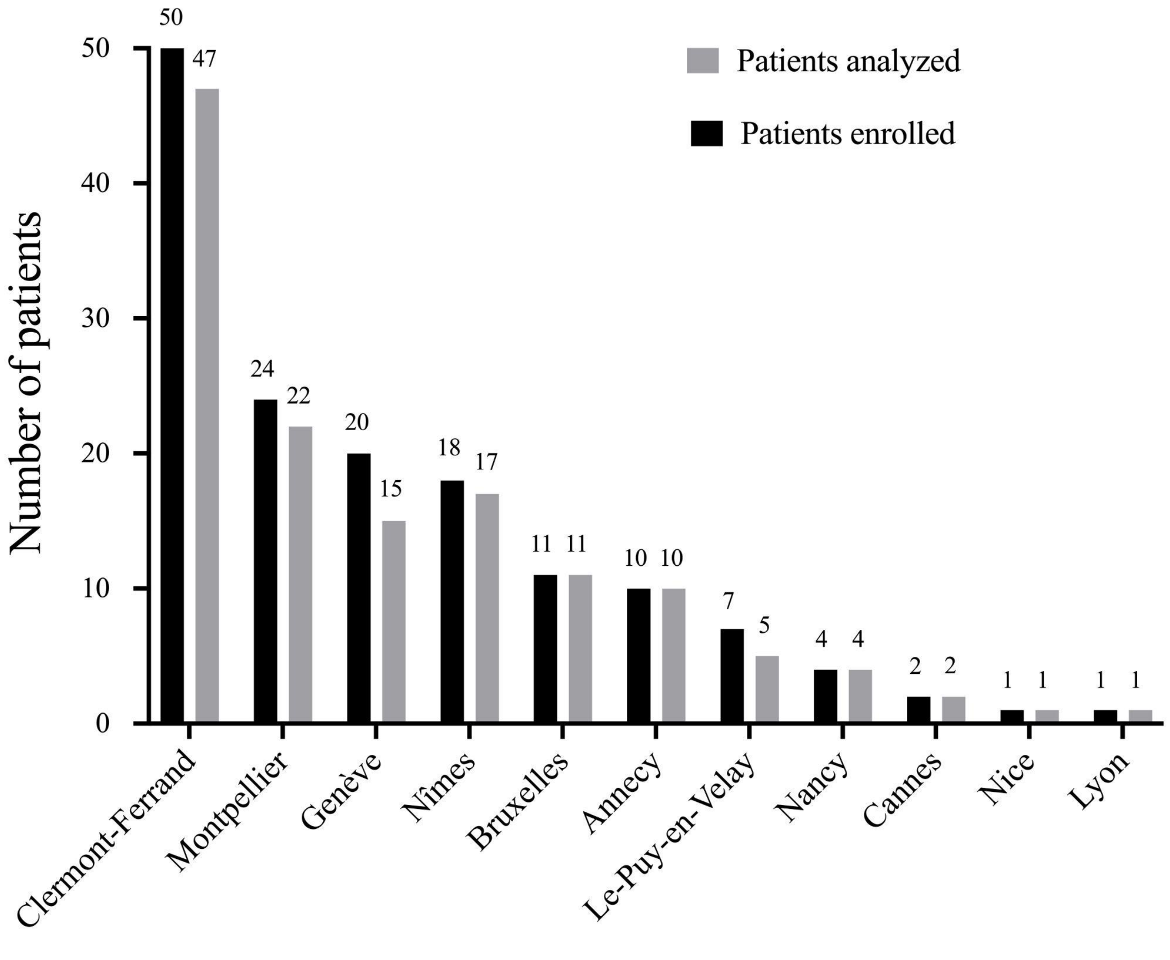

Supplementary Figure 1. Number of patients enrolled and analyzed in each participating center.

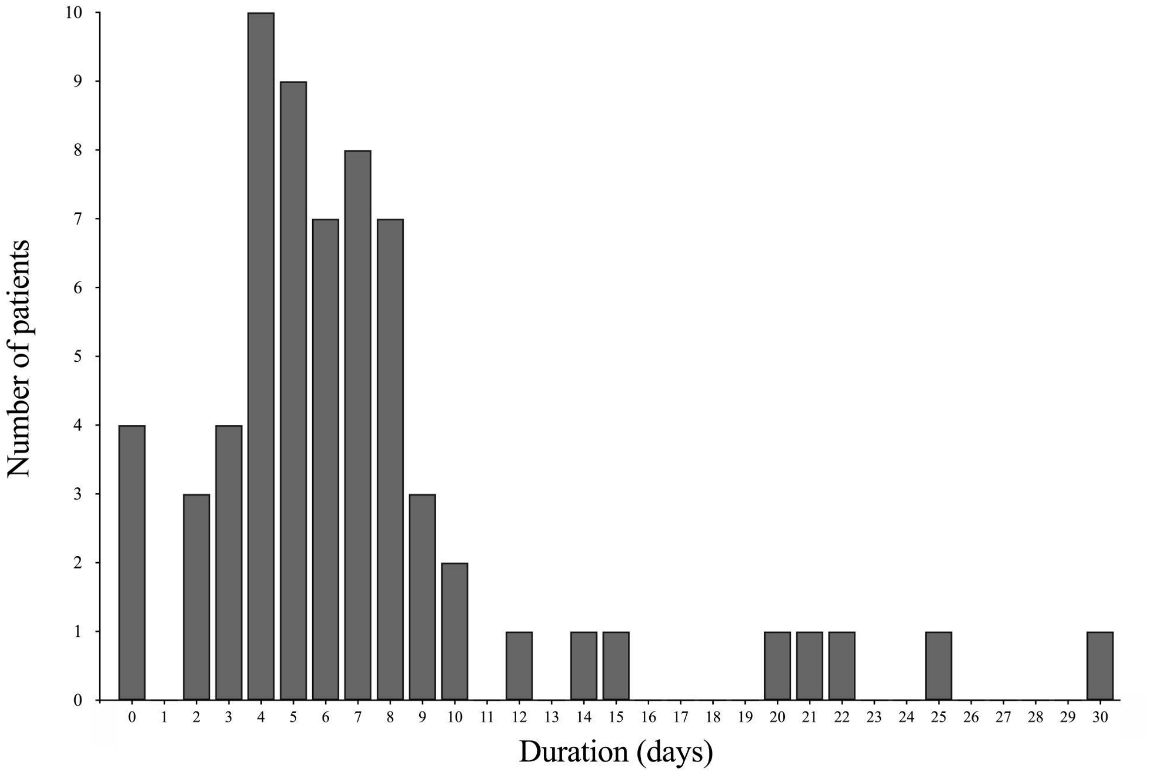

Supplementary Figure 2. Durations of epidural analgesia use in the trial.

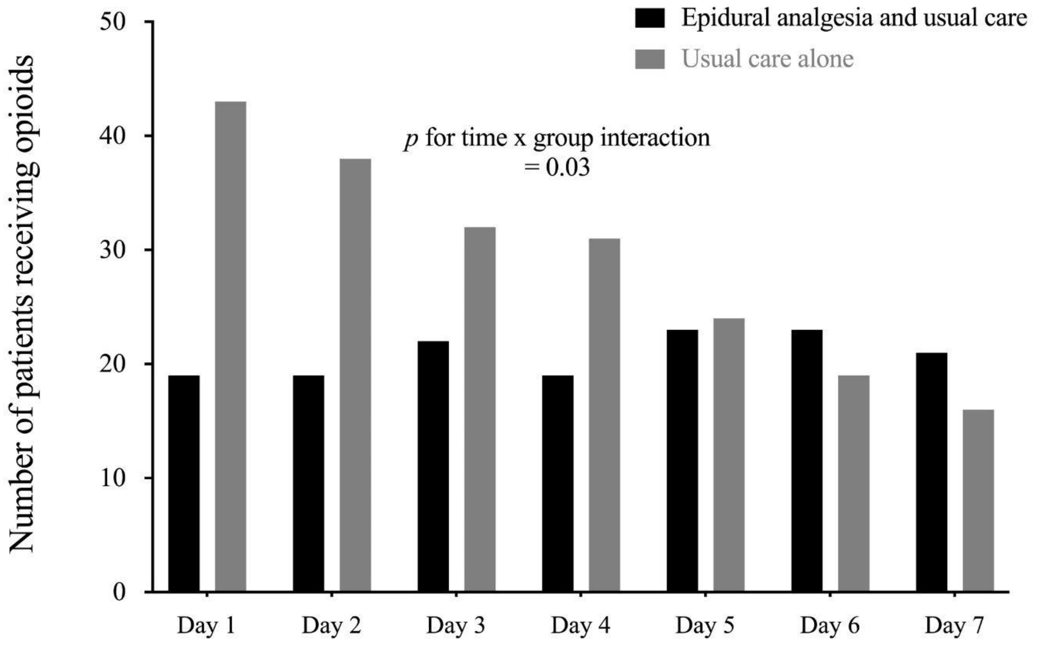

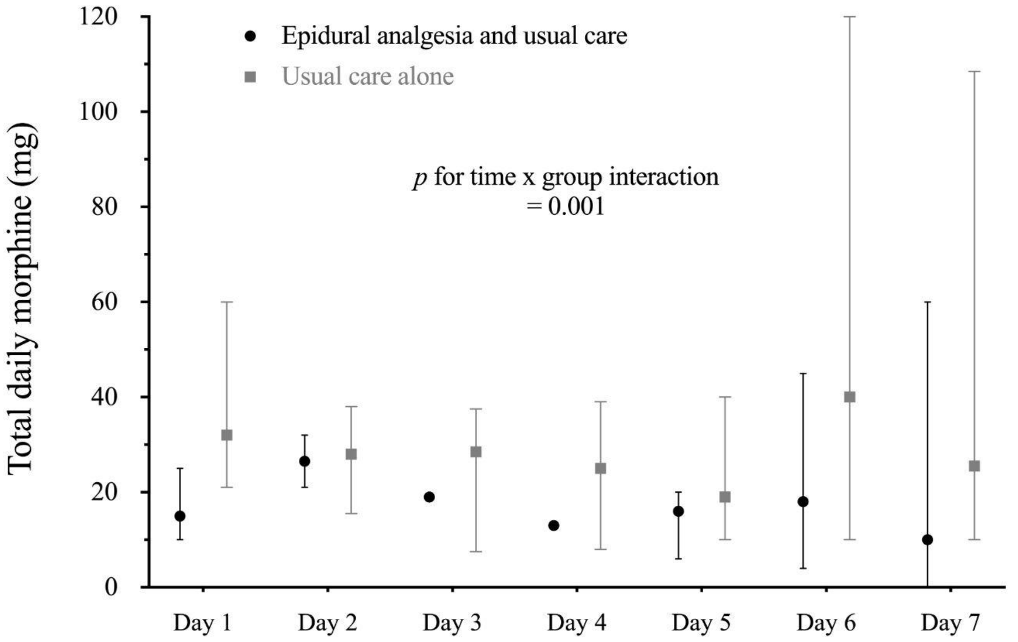

Supplementary Figure 3. Opioid requirements during the first week after randomization.

Top: Number of patients receiving opioids during the first week after randomization. *Bottom:* Total daily morphine (mg) during the first week after randomization.

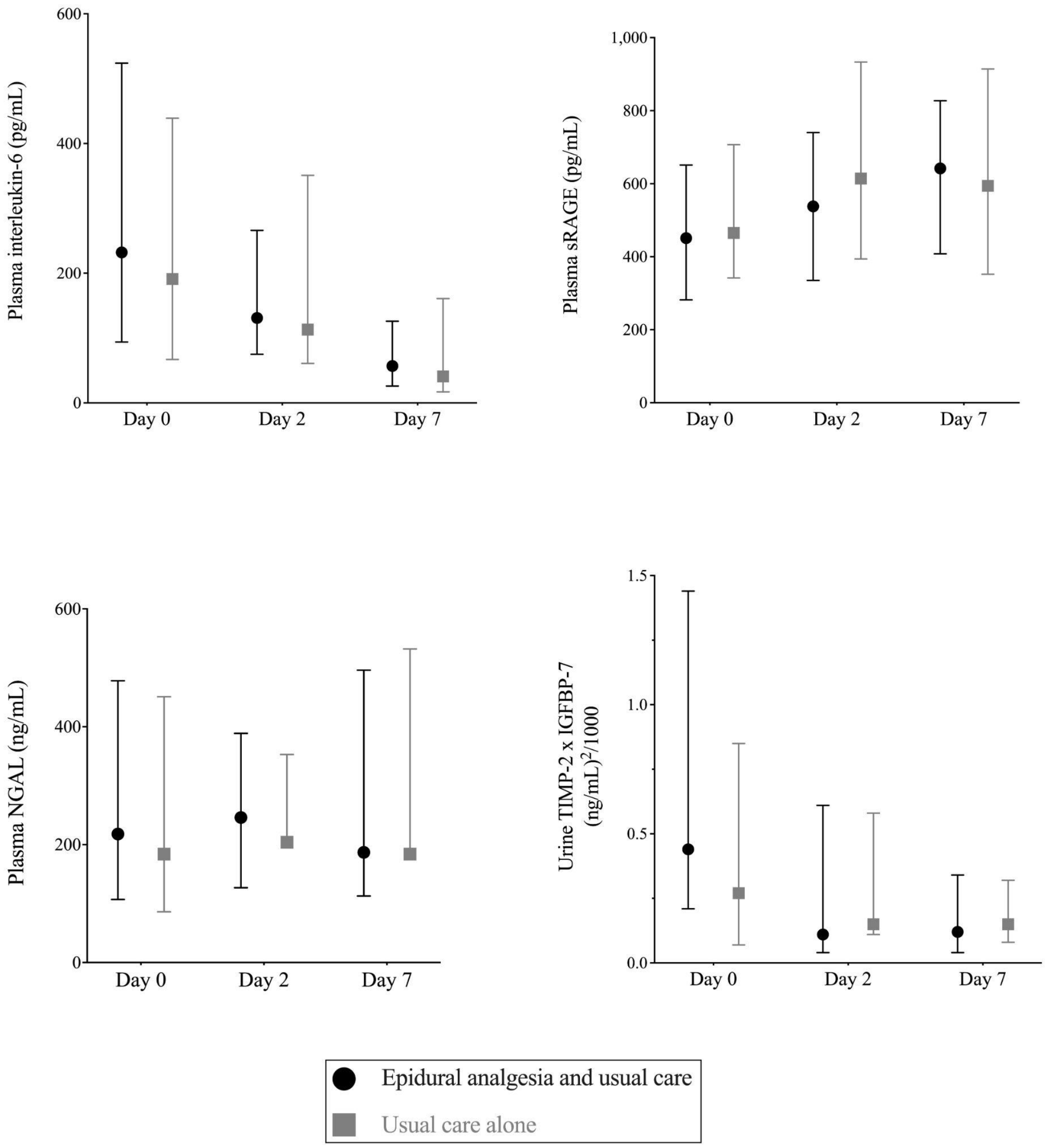

Supplementary Figure 4. Median biomarker levels during the first week after randomization.

Error bars indicate interquartile ranges. sRAGE: soluble receptor for advanced glycation end-products. NGAL: neutrophil gelatinase-associated lipocalin. TIMP-2: tissue inhibitor of metalloproteinases-2; IGFBP7: insulin-like growth factor-binding protein 7.

Supplementary Tables

Supplementary Table 1. Other characteristics of the patients at baseline.*			
**Characteristic**	**No. of observations available**	**Epidural analgesia and usual care (n=65)**	**Usual care alone (n=70)**
Ranson criteria for acute pancreatitis			
Age >55 years — no. (%)	134	34 (53)	37 (53)
Blood glucose >11 mmol/L — no. (%)	133	14 (22)	15 (21)
Blood leukocytes >16 G/L — no. (%)	132	21 (33)	23 (33)
Serum lactate dehydrogenase >350 IU/L — no. (%)	131	16 (25)	13 (19)
Serum aspartate aminotransferase >250 IU/L — no. (%)	130	7 (11)	6 (9)
Serum bicarbonate drop >4 mmol/L within 48 hours — no. (%)	129	9 (15)	6 (9)
Partial pressure of arterial oxygen <60 mmHg within 48 hours — no. (%)	129	5 (8)	13 (19)
Blood urea nitrogen increase >1.8 mmol/L within 48 hours — no. (%)	134	25 (39)	24 (34)
Serum calcium <2 mmol/L within 48 hours — no. (%)	131	26 (42)	25 (36)
Hematocrit drop >10% within 48 hours — no. (%)	131	25 (40)	22 (32)
Fluid needs > 6 L within 48 hours — no. (%)	130	1 (2)	3 (4)
Computed tomography grading of pancreatitis (Balthazar score)	134		
A: normal pancreas — no. (%)		1 (2)	4 (6)
B: enlargement of pancreas — no. (%)		2 (3)	1 (1)
C: inflammatory changes in pancreas and peripancreatic fat — no. (%)		10 (15)	7 (10)
D: single peripancreatic fluid collection — no. (%)		5 (8)	11 (16)
E: two or more poorly defined peripancreatic fluid collections — no. (%)		40 (62)	43 (62)
Not assessed — no. (%)		7 (11)	3 (4)
Sepsis — no. (%)†	134		
Absent		38 (59)	46 (66)
Suspected		21 (33)	16 (23)
Confirmed		3 (5)	4 (6)
Unknown		2 (3)	4 (6)
Severe sepsis — no. (%)†	43	15 (63)	6 (32)
Positive blood culture — no. (%)	135	1 (2)	2 (3)
Intra-abdominal infection — no. (%)	135	0 (0)	1 (1)
Pneumonia — no. (%)	135	1 (2)	1 (1)
Community-acquired pneumonia — no. (%)		1 (2)	1 (1)
Urinary tract infection — no. (%)	135	1 (2)	1 (1)
Antibiotic therapy — no. (%)	135	24 (37)	19 (27)
Empiric — no. (%)		24 (100)	17 (89)
Culture-documented — no. (%)		0 (0)	2 (11)
Antifungal therapy — no. (%)	134	0 (0)	2 (3)
Empiric — no. (%)		0 (0)	1 (50)
Culture-documented — no. (%)		0 (0)	1 (50)
Intra-abdominal hemorrhage — no. (%)	135	1 (2)	0 (0)
Ongoing treatment	135		
Anticoagulant therapy — no. (%)		3 (5)	3 (4)
Antiplatelet therapy — no. (%)		12 (18)	6 (9)
Nutrition during the previous month — no. (%)	133		
Normal oral feeding		62 (95)	64 (94)
Enteral nutrition		2 (3)	1 (1)
Parenteral nutrition		0 (0)	2 (3)
Parenteral and enteral nutrition		1 (2)	1 (1)

Laboratory tests			
Hematocrit — %	133	39.9±7.4	41.3±12.7
Blood leukocytes — G/L	134	15.3±7.9	15.6±7.6
Prothrombin time — %	123	76±20	74±22
Activated cephalin time — second	104	35±17	36±19
Lactate — mmol/L	118	2.1±2.0	2.2±2.2
Serum creatinine — µmol/L	135	114±91	146±149
Serum bicarbonate — mmol/L	122	22±5	23±5
Serum sodium — mmol/L	135	138±5	138±5
Serum potassium — mmol/L	133	3.8±0.7	4.1±0.8
Arterial pH	117	7.39±0.1	7.37±0.1
Partial pressure of arterial oxygen — mmHg	109	80±22	78±21
Partial pressure of arterial carbon dioxide — mmHg	108	35±5	41±13
Blood glucose >11 mmol/L — no. (%)	129	16 (26)	19 (28)
Serum lipase — IU/L	91	5,398±14536	1,696±3461
Serum aspartate aminotransferase — IU/L	122	293±1,293	140±238
Serum alanine aminotransferase — IU/L	127	194±627	103±154
Serum alkaline phosphatase — IU/L	121	121±133	117±91
Serum gamma-glutamyl transferase — IU/L	120	284±313	246±250
Serum total bilirubin — µmol/L	125	24±20	26±22
Serum lactate dehydrogenase >350 IU/L — no. (%)	59	15 (63)	21 (60)

* Plus–minus values are means ± standard deviations. There were no significant between-group differences at baseline.

† Sepsis and severe sepsis were defined based on the criteria from the American College of Chest Physicians/Society of Critical Care Medicine Consensus Conference *(Crit Care Med. 1992;20:864-74)*.

IU: international unit.

Supplementary Table 2. Respiratory parameters of the patients at baseline.			
		**Epidural analgesia and usual care (n=65)**	**Usual care alone (n=70)**
Unassisted ventilation	Total number of patients — no.	49	53
	Ambient air — no.	17	23
	Mask or nasal cannula — no.	28	29
	High concentration mask — no.	1	0
	High-flow oxygen — no.	6	2
Non-invasive ventilation*	Total number of patients — no.	3	3
Invasive ventilation	Total number of patients — no.	13	14
	Pressure-support ventilation — no.	3	7
	Volume-controlled ventilation — no.	10	7
	Pressure-controlled ventilation — no.	0	0
Tidal volume — mL/kg of predicted body weight	Median [interquartile range]	7.0 [6.2−7.7]	7.2 [6.1−8.4]
Inspiratory plateau pressure — cmH_2O	Median [interquartile range]	22 [20-24]	18 [11−26]
Positive end-expiratory pressure — cmH_2O	Median [interquartile range]	10 [8−12]	10 [9−11]
Inspired fraction of oxygen — %	Median [interquartile range]	60 [46−73]	56 [40−7]
Arterial pH	Median [interquartile range]	7.39 [7.37−7.42]	7.37 [7.34−7.40]
Partial pressure in arterial oxygen — mmHg	Median [interquartile range]	80 [74−86]	78 [73−83]

* Non-invasive ventilation included pressure-support ventilation and the use of continuous positive airway pressure.

**Supplementary Table 3. Details on the initiation of epidural analgesia in patients randomized to the intervention group.**		
**Characteristic**	**No. of observations available**	**Value**
Time from randomization to epidural catheter insertion — min		
Median	61	119
Interquartile range		39−219
Epidural catheter insertion operator — no. (%)	61	
Anesthesiology resident (under supervision)		20 (33)
Senior anesthesiologist		41 (67)
Position of the patient during insertion — no. (%)	53	
Sitting		37 (70)
Lateral recumbent		16 (30)
Level of epidural catheter insertion — no. (%)	54	
Intervertebral space T6-T7		15 (28)
Intervertebral space T7-T8		21 (39)
Intervertebral space T8-T9		18 (33)
Number of punctures needed — no. (%)	50	
1		28 (56)
2		9 (18)
3		8 (16)
4		1 (2)
5		2 (1)
9		1 (1)
10		1 (1)
Depth of the epidural space — mm	52	
Median		60
Interquartile range		40−70
Subcutaneous tunneling of the catheter — no. (%)	55	25 (45)
Time from anticoagulant therapy interruption — hour	14	
Median		17
Interquartile range		12−20
Time from antiplatelet therapy interruption — day	6	
Median		2.5
Interquartile range		1−12
Use of a test-dose of local anesthetics after catheter insertion — no. (%)	53	33 (62)
Use of procedural sedation for catheter insertion — no. (%)	58	4 (7)
Sedation drug used — no. (%)	4	
Midazolam		1 (25)
Ketamine		1 (25)
Propofol		2 (50)
Complication during epidural catheter placement or epidural analgesia initiation — no. (%)	65	4 (6)
Blood vessel puncture	65	1 (2)
Dural puncture	65	1 (2)
Vasovagal syncope	65	1 (2)
Arterial hypotension*	65	1 (2)
Aspect of the puncture site upon epidural analgesia initiation — no. (%)		
Healthy	51	51 (100)
Settings for patient-controlled epidural analgesia		
Continuous infusion rate — mL/h	58	5.8±2.0
Bolus volume — mL	56	4.8±1.7
No minimal time set between boli — no. (%)	59	5 (8)
Minimal time between boli — min	54	16.4±10.7
No maximal dose set — no. (%)	59	31 (53)
Maximal dose — mL/h	28	22.9±0.6
Epidural administration of clonidine — no. (%)	60	2 (3)

*One episode of mild arterial hypotension potentially attributable to a bolus injection, with spontaneous resolution.

Supplementary Table 4. Courses of epidural analgesia during the first seven study days in patients randomized to the intervention group.

		Day 1	Day 2	Day 3	Day 4	Day 5	Day 6	Day 7
Aspect of the puncture site	No. of observations available	49	50	50	43	43	34	27
	Healthy — no.	49	49	49	40	41	30	24
	Inflammatory — no.	0	1	1	3	2	4	1
	Purulent — no.	0	0	0	0	0	0	2
Catheter removal	Number of patients — no.	1	2	10	9	13	9	7
	Medical decision (≥72 h after randomization and epidural analgesia not needed anymore) — no.	0	1	2	7	9	5	5
	Accidental withdrawal	0	0	6	1	4	3	0
	Withdrawal for suspected complication — no.	1	1	1	1	0	1	2
Catheter repositioning	Number of patients — no.	0	0	1	0	0	0	0
Catheter disconnection	Number of patients — no.	0	3	2	2	4	1	2
Time from anticoagulant therapy interruption and catheter removal — hours	No. of observations available	0	1	4	2	6	3	3
	Median [interquartile range]	--	24	14 [8−20]	12 [12−12]	11 [9−15]	24 [23−24]	14 [8−17]
Motor block	No. of observations available	61	60	59	52	48	37	38
	None	25	25	19	17	15	9	9
	Partial	0	0	1	0	0	0	0
	Complete	0	0	0	1	0	0	0
	Not evaluable	36	35	39	34	33	28	29
Sensory block	No. of observations available	58	59	57	49	48	36	37
	Present — no.	13	15	10	5	7	3	3
	Absent — no.	7	6	3	3	1	3	1
	Not evaluable — no.	38	38	44	41	40	30	33
**Settings for patient-controlled epidural analgesia**								
Continuous flow — mL/h	Median [interquartile range]	6 [5−8]	6 [5−8]	6 [5−8]	6 [5−8]	6 [5−8]	6 [4−8]	6 [5−8]
Bolus — mL	Median [interquartile range]	4 [4−5]	4 [4−5]	4 [4−5]	5 [4−6]	5 [4−6]	5 [5−6]	5 [5−6]
Setting of a refractory period	Number of patients — no.	53	53	49	41	33	22	19
Maximum dose — mL/4h	Number of patients — no.	33	33	29	25	20	15	12
	Median [interquartile range]	27 [15−30]	29 [18−32]	28 [18−32]	29 [22−32]	32 [23−34]	28 [24−32]	26 [21−31]
Epidural administration of clonidine	Number of patients — no.	2	1	1	0	0	0	0
	Median dose [interquartile range] — µg	113 [75−150]	300 [300−300]	150 [150−150]	0 [0−0]	0	0	0

Supplementary Table 5. Complications potentially attributable to epidural analgesia in patients randomized to the intervention group from randomization to day 30.		
**Characteristic**	**No. of observations available**	**Value**
Complication of epidural analgesia — no. (%)	65	1 (1.5)
Local anesthetic toxicity (cardiac or neurologic) — no. (%)		0 (0)
Bradycardia or hypotension — no. (%)		1 (1.5)*
Pruritus — no. (%)		0 (0)
Epidural, subdural, or subarachnoid hematoma — no. (%)		0 (0)
Meningitis — no. (%)		0 (0)
Epidural abscess — no. (%)		0 (0)
Other — no. (%)		0 (0)

* One episode of mild arterial hypotension potentially attributable to a bolus injection, with spontaneous resolution.

Supplementary Table 6. Hemodynamic parameters during the first seven days after randomization.									
			**Day 1**	**Day 2**	**Day 3**	**Day 4**	**Day 5**	**Day 6**	**Day 7**
Acute coronary syndrome	Epidural analgesia and usual care	No. of observations available	65	64	61	58	56	51	50
		Number of patients — no.	0	1	1	0	0	1	0
	Usual care alone	No. of observations available	70	67	57	54	48	46	44
		Number of patients — no.	0	0	0	0	0	0	0
Serum lactate — mmol/L	Epidural analgesia and usual care	No. of observations available	53	49	45	41	35	32	30
		Median [interquartile range]	2.0 [0.9−2.2]	1.6 [1.0−1.7]	1.4 [0.8−1.6]	1.2 [0.9−1.3]	1.3 [0.8−1.5]	1.4 [0.9−1.8]	1.5 [1.0−1.7]
	Usual care alone	No. of observations available	53	51	40	35	32	25	24
		Median [interquartile range]	1.2 [0.9−1.7]	1.3 [0.8−1.5]	1.4 [0.8−1.5]	1.2 [0.8−1.6]	1.2 [0.9−1.5]	1.2 [0.1−1.6]	1.2 [0.8−1.5]
Fluid therapy	Epidural analgesia and usual care	No. of observations available	65	64	62	57	55	50	50
		Number of patients — no.	41	22	14	9	16	22	11
		Median volume [interquartile range] of crystalloids — mL	1,000 [500−2,500]	500 [0−1,000]	500 [0−1,000]	500 [250−500]	100 [0−500]	500 [0−1,000]	250 [0−1,000]
		Median volume [interquartile range] of colloids — mL	0 [0−500]	0 [0−200]	200 [0−300]	200 [0−200]	200 [0−200]	100 [0−300]	200 [0−200]
	Usual care alone	No. of observations available	68	66	56	54	48	45	41
		Number of patients — no.	31	23	18	13	15	12	11
		Median volume [interquartile range] of crystalloids — mL	1,000 [980−3,000]	1,000 [500−2,500]	500 [0−1,000]	300 [0−1,000]	880 [250−2,500]	125 [0−1,000]	250 [0−524]
		Median volume [interquartile range] of colloids — mL	0 [0−0]	0 [0−100]	100 [0−500]	0 [0−312]	0 [0−200]	200 [0−330]	100 [0−200]
Total fluid intake	Epidural analgesia and usual care	Median volume [interquartile range] — mL	1,831 [1,089−2,557]	1,898 [1,500−2,454]	1833 [1,108−2,300]	1,641 [1,025−2,276]	1,551 [986−2,015]	1,521 [500−2,382]	1,250 [520−2,123]
	Usual care alone	Median volume [interquartile range] — mL	1,743 [1,176−2,672]	1,741 [1,000−2,500]	1,500 [765−2,160]	1,446 [636−1,957]	1,048 [596−1,919]	1,212 [336−2,242]	984 [120−2,272]
Blood transfusion	Epidural analgesia and usual care	No. of observations available	65	64	62	58	56	51	50
		Number of patients — no.	2	1	2	1	2	1	1

		Number of patients receiving red blood cells — no.	2	1	2	1	2	1	1
		Number of patients receiving fresh frozen plasma — no.	2	1	2	1	2	1	1
		Number of patients receiving platelets — no.	2	1	2	1	2	1	1
	Usual care alone	No. of observations available<br><br>Number of patients — no.	70<br><br>1	68<br><br>3	60<br><br>0	55<br><br>2	49<br><br>1	47<br><br>1	44<br><br>0
		Number of patients receiving red blood cells — no.	1	3	0	2	1	1	0
		Number of patients receiving fresh frozen plasma — no.	1	3	0	2	1	1	0
		Number of patients receiving platelets — no.	1	3	0	2	1	1	0
Vasopressors and inotropes	Epidural analgesia and usual care	No. of observations available	65	65	62	58	56	53	58
		Number of patients receiving norepinephrine — no.<br><br>Median dose [interquartile range] of norepinephrine — µg/kg/min	21<br><br>0.23 [0.10−0.32]	14<br><br>0.20 [0.13−0.80]	13<br><br>0.30 [0.10−0.59]	9<br><br>0.30 [0.10−0.28]	11<br><br>0.15 [0.06−0.44]	8<br><br>0.20 [0.10−0.44]	6<br><br>0.63 [0.20−0.70]
		Number of patients receiving dobutamine — no.<br><br>Median dose [interquartile range] of dobutamine — µg/kg/min	1<br><br>10.3 [10.3−10.3]	1<br><br>5.1 [5.1−5.1]	1<br><br>5.1 [5.1−5.1]	1<br><br>5.1 [5.1−5.1]	0<br><br>--	0<br><br>--	0<br><br>--
		Number of patients receiving epinephrine — no.<br><br>Median dose [interquartile range] of epinephrine — µg/kg/min	0<br><br>--	0<br><br>--	1<br><br>*Not available*	1<br><br>*Not available*	0<br><br>--	0<br><br>--	0<br><br>--
	Usual care alone	No. of observations available	70	69	61	56	52	50	54
		Number of patients receiving norepinephrine — no.	18	13	9	6	6	4	3

		Median dose [interquartile range] of norepinephrine — µg/kg/min	0.23 [0.10−0.67]	0.39 [0.20−0.60]	0.14 [0.13−0.30]	0.46 [0.40−0.55]	0.30 [0.20−0.43]	0.30 [0.12−0.42]	0.47 [0.40−0.53]
		Number of patients receiving dobutamine — no.<br><br>Median dose [interquartile range] of dobutamine — µg/kg/min	2<br><br>3.5 [2.5−7.5]	0<br><br>--	0<br><br>--	0<br><br>--	0<br><br>--	0<br><br>--	0<br><br>--
		Number of patients receiving epinephrine — no.<br><br>Median dose [interquartile range] of epinephrine — µg/kg/min	2<br><br>0.90 [0.20−1.60]	1<br><br>*Not available*	0<br><br>--	1<br><br>*Not available*	0<br><br>--	0<br><br>--	0<br><br>--

Supplementary Table 7. Respiratory parameters during the first seven days after randomization.

			Day 1	Day 2	Day 3	Day 4	Day 5	Day 6	Day 7
Unassisted ventilation	Epidural analgesia and usual care	Number of patients — no.	46	41	39	39	35	28	31
		Ambient air — no.	17	18	19	18	20	13	14
		Mask or nasal cannula — no.	26	20	17	19	14	14	13
		High concentration mask — no.	0	0	0	0	0	0	1
		High-flow oxygen — no.	5	6	6	2	1	1	2
	Usual care alone	Number of patients — no.	47	41	36	33	30	30	26
		Ambient air — no. (%)	19	17	14	11	12	14	15
		Mask or nasal cannula — no. (%)	23	23	21	20	15	13	15
		High concentration mask — no. (%)	2	2	0	0	1	2	0
		High-flow oxygen — no. (%)	5	3	4	4	3	3	3
Oxygen flow — L/min	Epidural analgesia and usual care	No. of observations available	38	33	34	35	30	25	27
		Median value [interquartile range]	3 [0−3]	3 [0−3]	2 [0−3]	1 [0−2]	1 [0−2]	1 [0−2]	2 [0−3]
	Usual care alone	No. of observations available	44	41	36	32	28	28	24
		Median value [interquartile range]	4 [0−4]	4 [0−4]	5 [0−4]	7 [4−5]	4 [0−4]	2 [0−3]	3 [0−3]
Non-invasive ventilation*	Epidural analgesia and usual care	Number of patients — no.	2	3	3	0	1	0	0
	Usual care alone	Number of patients — no.	2	4	4	2	1	2	2
Invasive ventilation	Epidural analgesia and usual care	Number of patients — no.	15	18	18	17	18	19	16
		Pressure-support ventilation	4	9	10	9	9	13	9
		Volume-controlled ventilation	11	9	7	7	8	4	6
		Pressure-controlled ventilation	0	0	1	1	1	2	1
	Usual care alone	Number of patients — no.	18	18	15	18	16	13	14
		Pressure-support ventilation	10	13	12	10	10	11	11
		Volume-controlled ventilation	8	5	3	7	6	2	3
		Pressure-controlled ventilation	0	0	0	1	0	0	0
Tidal volume, mL/kg of predicted body weight	Epidural analgesia and usual care	No. of observations available	15	16	17	16	17	19	16
		Median value [interquartile range]	6.7 [6.0−8.0]	7.2 [6.3−8.7]	7.3 [6.4−7.8]	6.9 [6.4−7.8]	6.6 [6.4−7.5]	7.5 [6.7−8.9]	7.3 [6.3−8.6]
	Usual care alone	No. of observations available	17	16	14	17	15	12	13
		Median value [interquartile range]	6.8 [6.3−7.9]	6.8 [6.0−8.1]	7.1 [6.4−9.0]	7.0 [6.4−7.5]	7.2 [5.6−7.7]	7.4 [6.7−10.0]	6.4 [5.7−7.5]
Inspiratory plateau pressure — cmH_2O	Epidural analgesia and usual care	No. of observations available	11	8	9	9	9	9	8
		Median value [interquartile range]	24 [20−28]	25 [20−28]	26 [20−29]	20 [15−23]	21 [19−23]	20 [18−22]	24 [22−27]
	Usual care alone	No. of observations available	11	9	7	11	10	6	5
		Median value [interquartile range]	22 [16−28]	19 [12−26]	21 [10−26]	22 [16−30]	23 [16−29]	21 [18−25]	22 [18−30]
Positive end-expiratory pressure — cmH_2O	Epidural analgesia and usual care	No. of observations available	16	20	18	15	18	18	15
		Median value [interquartile range]	9 [7−12]	9 [6−10]	9 [7−10]	8 [6−10]	8 [6−8]	8 [6−10]	8 [6−10]
	Usual care alone	No. of observations available	17	19	16	16	15	13	14
		Median value [interquartile range]	10 [8−12]	10 [7−12]	10 [7−15]	11 [8−13]	11 [8−12]	10 [8−12]	10 [8−12]
Inspired fraction of oxygen — %	Epidural analgesia and usual care	No. of observations available	21	24	24	19	20	19	18
		Median value [interquartile range]	55 [40−60]	55 [38−78]	56 [35−75]	45 [35−50]	45 [35−50]	47 [35−60]	42 [30−50]
	Usual care alone	No. of observations available	24	24	22	24	19	16	17

		Median value [interquartile range]	58 [38−100]	52 [33−60]	51 [40−50]	56 [40−73]	52 [30−73]	51 [33−55]	47 [35−50]
Partial pressure in arterial oxygen — mmHg	Epidural analgesia and usual care	No. of observations available	43	46	44	37	36	31	31
		Median value [interquartile range]	76 [64−88]	80 [66−88]	83 [68−88]	79 [66−89]	77 [65−84]	78 [66−89]	76 [65−80]
	Usual care alone	No. of observations available	52	46	38	35	31	22	21
		Median value [interquartile range]	73 [60−82]	72 [62−81]	70 [63−75]	76 [61−88]	82 [64−88]	76 [61−82]	74 [67−83]
Arterial pH	Epidural analgesia and standard care	No. of observations available	48	48	45	39	38	32	33
		Median value [interquartile range]	7.38 [7.34−7.43]	7.39 [7.32−7.46]	7.42 [7.40−7.46]	7.45 [7.41−7.49]	7.44 [7.40−7.48]	7.46 [7.41−7.51]	7.46 [7.44−7.49]
	Standard care alone	No. of observations available	54	47	40	35	31	22	21
		Median value [interquartile range]	7.38 [7.32−7.44]	7.40 [7.36−7.47]	7.41 [7.37−7.47]	7.41 [7.38−7.46]	7.42 [7.41−7.50]	7.45 [7.4−7.50]	7.45 [7.43−7.50]

* Non-invasive ventilation included pressure-support ventilation and the use of continuous positive airway pressure.

Supplementary Table 8. Neurological parameters and use of sedatives and analgesics during the first seven days after randomization.

			Day 1	Day 2	Day 3	Day 4	Day 5	Day 6	Day 7
Communicating patient	Epidural analgesia and usual care	No. of observations available	64	64	62	58	56	50	49
		Number of patients — no.	51	48	44	44	42	35	36
	Usual care alone	No. of observations available	70	68	59	55	49	44	43
		Number of patients — no.	57	52	45	39	35	35	33
Sedation	Epidural analgesia and usual care	No. of observations available	64	64	62	58	55	50	50
		Number of patients under sedation — no.	18	17	17	17	14	14	14
		Midazolam	1	1	1	1	0	1	1
		Propofol	12	10	8	10	9	8	9
		Dexmedetomidine	3	4	6	4	3	3	2
		Sevoflurane	1	1	1	1	1	1	1
		Other	1	1	1	1	1	1	1
	Usual care alone	No. of observations available	70	66	60	55	49	47	44
		Number of patients under sedation — no.	17	15	13	17	15	12	14
		Midazolam	2	1	0	0	0	0	1
		Propofol	11	9	7	11	10	8	9
		Dexmedetomidine	4	4	5	5	5	4	3
		Sevoflurane	0	0	0	0	0	0	0
		Other	0	1	1	1	0	0	1
Richmond agitation sedation scale	Epidural analgesia and usual care	No. of observations available	15	15	15	15	13	13	13
		Number of patients — no.							
		+4	0	0	0	1	0	0	0
		+3	0	0	0	0	0	0	0
		+2	0	1	0	0	1	0	0
		+1	0	0	0	1	0	0	0
		0	3	2	2	3	0	1	1
		-1	1	1	3	1	3	3	4
		-2	0	2	1	0	1	0	1
		-3	3	1	1	3	2	3	2
		-4	3	2	14	3	3	3	2
		-5	1	4	2	1	2	1	2
		Not evaluable (under neuromuscular blockade)	4	2	2	2	1	2	1
	Usual care alone	No. of observations available	18	16	13	16	15	12	12
		Number of patients — no.							
		+4	0	0	0	0	0	0	0
		+3	0	0	0	0	0	0	0
		+2	1	0	0	0	0	0	0
		+1	0	0	0	1	0	1	0
		0	2	6	7	5	3	3	3
		-1	3	1	2	1	2	1	2
		-2	2	5	0	0	1	1	2
		-3	3	0	0	3	2	2	3
		-4	3	1	1	1	4	3	1
		-5	1	2	2	2	0	0	0
		Not evaluable (under neuromuscular blockade)	3	1	1	3	3	1	1
Visual analogue score for pain at rest (in communicating patients)	Epidural analgesia and usual care	No. of observations available	49	46	44	43	41	34	35
		Median value [interquartile range]	2 [0−3]	2 [0−3]	2 [0−3]	2 [0−3]	2 [0−4]	2 [0−4]	2 [0−4]
		No. of patients with a visual analogue score below 40/100	26	30	26	27	28	28	32
	Usual care alone	No. of observations available	55	49	45	39	34	34	31
		Median value [interquartile range]	2 [0−5]	2 [0−5]	1 [0−4]	2 [0−4]	1 [0−3]	1 [0−2]	0 [0−3]
		No. of patients with a visual analogue score below 40/100	34	36	28	28	26	20	27
Visual analogue score for pain during nursing procedures (in communicating patients)	Epidural analgesia and usual care	No. of observations available	45	43	42	39	35	32	34
		Median value [interquartile range]	2 [0−4]	2 [0−5]	1 [0−3]	2 [0−3]	0 [0−4]	2 [0−3]	2 [0−5]
		No. of patients with a visual analogue score below 40/100	36	40	29	28	30	25	34
	Usual care alone	No. of observations available	50	46	43	38	34	34	31
		Median value [interquartile range]	4 [2−6]	2 [0−5]	3 [0−5]	2 [0−4]	3 [0−4]	1 [0−3]	0 [0−1]

		No. of patients with a visual analogue score below 40/100	49	43	36	33	33	23	29
Behavioral pain scale at rest (in non-communicating patients)	Epidural analgesia and usual care	No. of observations available	9	14	15	14	14	14	13
		Median value [interquartile range]	3 [3−3]	3 [3−3]	3 [3−3]	3 [3−3]	3 [3−3]	3 [3−3]	3 [3−3]
		No. of patients with a behavioral pain scale of 3-4	8	14	14	13	13	13	12
	Usual care alone	No. of observations available	11	14	13	14	11	8	9
		Median value [interquartile range]	3 [3−3]	3 [3−4]	3 [3−3]	3 [3−3]	3 [3−4]	3 [3−3]	3 [3−3]
		No. of patients with a behavioral pain scale of 3-4	10	11	12	12	9	7	9
Behavioral pain scale during nursing procedures (in non-communicating patients)	Epidural analgesia and usual care	No. of observations available	9	12	15	13	13	13	12
		Median value [interquartile range]	3 [3−3]	3 [3−3]	3 [3−4]	3 [3−3]	3 [3−4]	3 [3−4]	3 [3−3]
		No. of patients with a behavioral pain scale of 3-4	8	11	14	12	12	12	10
	Usual care alone	No. of observations available	12	14	13	14	11	8	9
		Median value [interquartile range]	3 [3−3]	4 [3−5]	3 [3−4]	3 [3−4]	3 [3−4]	3 [3−5]	3 [3−4]
		No. of patients with a behavioral pain scale of 3-4	11	8	11	11	9	6	7
Analgesics	Epidural analgesia and usual care	No. of observations available	65	65	62	58	56	53	58
		None — no.	13	16	15	16	15	8	5
		WHO's pain relief ladder step 1 drug — no.	38	35	33	26	32	29	29
		WHO's pain relief ladder step 2 drug — no.	6	7	6	8	9	7	8
		WHO's pain relief ladder step 3 drug — no.	19	19	21	19	23	21	22
	Usual care alone	No. of observations available	70	69	61	56	52	50	54
		None — no.	6	3	5	6	9	11	11
		WHO's pain relief ladder step 1 drug — no.	41	42	38	36	28	22	18
		WHO's pain relief ladder step 2 drug — no.	21	19	17	16	11	9	7
		WHO's pain relief ladder step 3 drug — no.	42	39	33	30	23	19	16
Route of administration of analgesics	Epidural analgesia and usual care	Oral — no.	6	4	5	7	5	8	8
		Enteral — no.	41	37	38	3	33	28	28
		Intravenous — no.	5	5	4	2	3	6	5
	Usual care alone	Oral — no.	7	4	6	7	5	8	8
		Enteral — no.	49	52	41	32	27	25	22
		Intravenous — no.	6	6	5	7	5	1	0
Opioids	Epidural analgesia and usual care	No. of observations available	65	65	62	58	56	53	58
		Number of patients — no.	5	4	4	1	3	4	3
		Daily dose of morphine, median [interquartile range] — mg	15 [10−25]	27 [21−32]	19 [19-19]	13 [13-13]	14 [6-20]	2 [4-45]	23 [0-60]
		Number of patients — no.	0	0	0	1	1	2	1
		Daily dose of fentanil, median [interquartile range] — µg	--	--	--	1,325 [1,325−1,325]	1,318 [1,318−1,318]	1,251 [898−1,603]	480 [480−480]
		Number of patients — no.	2	1	3	4	4	4	5
		Daily dose of sufentanil, median [interquartile range] — µg	70 [50−80]	190 [20−260]	145 [10−280]	360 [240−480]	480 [140−530]	480 [260−720]	840 [320−953]
		Number of patients — no.	9	11	12	10	12	12	10
		Daily dose of remifentanil, median [interquartile range] — µg	12,248 [6,798−17,620]	10,164 [4,384−14,414]	10,163 [2,953−16,951]	13,438 [6,432−18,520]	11,278 [2,552−17,009]	9,699 [953−14,812]	10,727 [7,506−14,969]
		Other — no.	3	3	3	3	3	1	2
	Usual care alone	No. of observations available	70	69	61	56	52	50	54
		Number of patients — no.	26	20	15	14	11	9	7
		Daily dose of morphine, median [interquartile range] — mg	52 [21−60]	29 [16−38]	25 [8−38]	30 [8−39]	34 [10−40]	65 [10−120]	50 [10−109]
		Number of patients — no.	2	1	1	1	1	1	1

		Daily dose of fentanil, median [interquartile range] — µg	1,347 [225−2,469]	2,469 [2,469−2,469]	1,233 [1,233−1,233]	25 [25−25]	25 [25−25]	25 [25−25]	58 [58−58]
		Number of patients — no.	5	7	8	5	2	0	0
		Daily dose of sufentanil, median [interquartile range] — µg	*Not available*	112 [53−171]	182 [85−360]	*Not available*	*Not available*	--	--
		Number of patients — no.	10	9	8	10	10	9	8
		Daily dose of remifentanil, median [interquartile range] — µg	30,226 [12,811−24,648]	13,444 [7,162−20,281]	14,857 [8,767−20,400]	11,443 [4,800−16,848]	15,493 [4,838−33,242]	13,534 [4,838−15,648]	11,700 [4,007−16,591]
		Other — no.	0	1	0	1	0	0	0

WHO: World Health Organization.

Supplementary Table 9. Renal parameters during the first seven days after randomization.

			Day 1	Day 2	Day 3	Day 4	Day 5	Day 6	Day 7
Urine output	Epidural analgesia and usual care	No. of observations available	63	62	60	56	49	46	42
		Median value [interquartile range] — mL	1,846 [1,000−2,250]	1,999 [1,200−2,400]	2,342 [1,503−3,198]	2,245 [1,500−2,965]	2,291 [1,350−3,000]	2,342 [1,420−2,950]	2,416 [1,485−2,850]
	Usual care alone	No. of observations available	67	64	54	48	45	41	36
		Median value [interquartile range] — mL	1,737 [900−2,275]	2,242 [1,180−2,505]	2,255 [1,250−2,870]	2,096 [925−3,115]	2,191 [850−3,450]	2,124 [1,070−2,850]	1,802 [1,230−2,460]
Need for renal replacement therapy	Epidural analgesia and usual care	No. of observations available	61	58	57	53	52	49	47
		Number of patients — no.	4	6	5	5	4	2	3
		Hemofiltration	2	4	4	2	2	1	1
		Hemodialysis	1	0	0	0	0	0	0
		Hemodiafiltration	1	2	1	2	2	1	2
	Usual care alone	No. of observations available	63	61	53	48	43	43	39
		Number of patients — no.	7	6	7	7	6	4	5
		Hemofiltration	5	5	6	5	5	3	4
		Hemodialysis	1	1	0	1	1	1	1
		Hemodiafiltration	1	0	1	1	0	0	0
Use of diuretics	Epidural analgesia and usual care	No. of observations available	59	54	54	45	44	38	37
		Number of patients — no.	6	10	8	13	12	13	13
	Usual care alone	No. of observations available	64	53	43	41	37	34	34
		Number of patients — no.	6	12	16	14	12	11	9
Fluid loss >500 mL/24h (except urine output)	Epidural analgesia and usual care	No. of observations available	52	52	54	49	46	44	38
		Number of patients — no.	11	10	7	8	6	6	9
	Usual care alone	No. of observations available	59	59	49	45	37	37	35
		Number of patients — no.	9	9	11	7	11	9	6
Volume of fluid loss	Epidural analgesia and usual care	No. of observations available	10	9	7	8	6	6	9
		Median value [interquartile range] — mL/24h	1,500 [1,150−2,045]	950 [800−1,425]	1,550 [700−1,750]	1,575 [730−2,045]	1,305 [650−1,695]	1,093 [800−2,200]	1,100 [700−1,803]
	Usual care alone	No. of observations available	9	9	11	7	11	9	6
		Median value [interquartile range] — mL/24h	1,240 [850−1,600]	900 [800−1,020]	1,200 [950−1,500]	1,500 [800−4,389]	900 [600−1,750]	800 [600−1,100]	813 [650−900]
Urine catheter	Epidural analgesia and usual care	No. of observations available	65	65	62	58	55	51	50
		Number of patients — no.	48	49	49	41	37	33	34
	Usual care alone	No. of observations available	70	68	60	54	49	46	43
		Number of patients — no.	56	54	45	41	37	35	30
Urine retention	Epidural analgesia and usual care	No. of observations available	17	14	13	16	17	18	16
		Number of patients — no.	1	0	0	0	0	0	0
	Usual care alone	No. of observations available	13	13	14	13	12	11	13
		Number of patients — no.	0	0	0	0	0	0	0

Supplementary Table 10. Abdominal complications during the first seven days after randomization.									
			Day 1	Day 2	Day 3	Day 4	Day 5	Day 6	Day 7
Computed tomography-scan performed	Epidural analgesia and usual care	Number of patients — no.	8	7	7	5	8	1	2
	Usual care alone	Number of patients — no.	9	7	5	4	3	6	2
Extension of pancreatic necrosis	Epidural analgesia and usual care	None — no.	5	3	2	1	2	0	0
		<30% — no.	1	2	3	3	2	0	0
		30-50% — no.	1	0	0	0	1	1	1
		>50% — no.	1	0	2	0	1	0	1
	Usual care alone	None — no.	4	2	1	3	1	0	1
		<30% — no.	2	2	0	1	0	0	1
		30-50% — no.	0	2	1	0	1	2	0
		>50% — no.	3	1	1	0	1	2	0
Intra-abdominal collection	Epidural analgesia and usual care	Number of patients — no.	2	2	5	2	3	0	2
	Usual care alone	Number of patients — no.	4	2	2	2	2	3	0
Peripancreatic necrosis	Epidural analgesia and usual care	Number of patients — no.	4	4	2	2	3	1	1
	Usual care alone	Number of patients — no.	4	5	1	3	1	2	1
Infected necrosis	Epidural analgesia and usual care	Number of patients — no.	0	0	1	0	0	0	0
	Usual care alone	Number of patients — no.	0	0	0	0	1	0	0
Necrosectomy	Epidural analgesia and usual care	Number of patients — no.	1	0	0	0	0	1	0
		Surgical management — no.	1	0	0	0	0	1	0
	Usual care alone	Number of patients — no.	0	0	0	0	0	1	0
		Endoscopic treatment — no.	0	0	0	0	0	1	0
Intra-abdominal hemorrhage	Epidural analgesia and usual care	Number of patients — no.	0	0	0	0	0	0	0
	Usual care alone	Number of patients — no.	1	1	0	0	0	1	0
		No treatment — no.	0	0	0	0	0	1	0
		Blood transfusion — no.	0	0	0	0	0	0	0
		Radiological embolisation — no.	1	1	0	0	0	0	0
		Surgical treatment — no.	1	1	0	0	0	0	0
Intra-abdominal thrombosis	Epidural analgesia and usual care	Number of patients — no.	2	2	1	1	1	1	1
	Usual care alone	Number of patients — no.	4	4	4	4	5	4	4
Mesenteric thrombosis	Epidural analgesia and usual care	Number of patients — no.	2	2	1	1	1	1	1
	usual care alone	Number of patients — no.	3	3	2	1	2	0	1
Bowel perforation	Epidural analgesia and usual care	Number of patients — no.	1	1	1	0	1	1	1
	Usual care alone	Number of patients — no.	0	0	0	1	0	0	0

Supplementary Table 11. Septic complications during the first seven days after randomization.									
			**Day 1**	**Day 2**	**Day 3**	**Day 4**	**Day 5**	**Day 6**	**Day 7**
Sepsis*	Epidural analgesia and usual care	No. of observations available	65	64	62	58	56	51	50
		None — no.	40	32	31	28	29	23	23
		Suspected — no.	18	23	24	19	16	16	14
		Confirmed — no.	6	8	7	10	10	11	12
		Unknown — no.	1	1	0	1	1	1	1
	Usual care alone	No. of observations available	68	65	57	53	48	46	43
		None — no.	42	43	38	32	27	26	21
		Suspected — no.	16	13	12	11	12	8	7
		Confirmed — no.	8	7	6	8	8	10	13
		Unknown — no.	2	2	1	2	1	2	2
Severe sepsis*	Epidural analgesia and usual care	No. of observations available	24	30	30	29	26	27	26
		Number of patients — no.	12	14	11	8	8	9	7
	Usual care alone	No. of observations available	22	19	18	18	19	18	20
		Number of patients — no.	8	4	3	2	3	4	4
Antibiotic therapy	Epidural analgesia and usual care	No. of observations available	65	63	62	58	56	51	49
		Empiric — no.	22	24	21	17	15	16	15
		Documented infection — no.	3	4	8	11	13	13	13
	Usual care alone	No. of observations available	70	68	60	55	49	47	44
		Empiric — no.	17	18	14	16	16	12	10
		Documented infection — no.	5	5	7	7	8	14	15
Antifungal therapy	Epidural analgesia and usual care	No. of observations available	64	63	61	58	56	51	50
		Empiric — no.	0	0	0	0	0	0	1
		Documented — no.	0	1	2	1	1	2	2
	Usual care alone	No. of observations available	70	68	60	55	49	47	44
		Empiric — no.	2	2	2	2	2	2	2
		Documented — no.	0	1	1	1	1	1	1
Sepsis localisation (confirmed sepsis)	Epidural analgesia and usual care	Bacteremia — no.	2	4	4	4	3	4	5
		Intra-abdominal — no.	2	2	2	1	2	2	3
		Pneumonia — no.	2	2	1	2	3	4	4
		Urinary infection — no.	1	1	1	3	2	2	2
		Skin infection — no.	0	0	0	0	0	0	0
		Neurological infection — no.	0	0	0	0	0	0	0
		Catheter infection — no.	0	0	0	1	2	3	2
	Usual care alone	Bacteremia — no.	2	3	2	3	3	2	4
		Intra-abdominal — no.	4	2	1	1	1	2	2
		Pneumonia — no.	3	3	3	4	4	5	6
		Urinary infection — no.	1	0	0	0	0	1	1
		Skin infection — no.	0	0	0	0	0	0	0
		Neurological infection — no.	0	0	0	0	0	0	0
		Catheter infection — no.	0	0	0	0	0	0	1

* Sepsis and severe sepsis were defined based on the criteria from the American College of Chest Physicians/Society of Critical Care Medicine Consensus Conference *(Crit Care Med. 1992;20:864-74)*.

Supplementary Table 12. Criteria for systemic inflammatory response syndrome during the first seven days after randomization.									
			**Day 1**	**Day 2**	**Day 3**	**Day 4**	**Day 5**	**Day 6**	**Day 7**
Body temperature <36°C or >38°C	Epidural analgesia and usual care	No. of observations available	65	64	62	58	56	51	50
		Number of patients — no.	28	24	23	22	23	18	18
	Usual care alone	No. of observations available	70	67	60	55	49	46	43
		Number of patients — no.	20	25	20	16	16	15	13
Heart rate >90 beats per minute	Epidural analgesia and usual care	No. of observations available	65	62	62	58	56	51	50
		Number of patients — no.	57	48	44	45	41	36	35
	Usual care alone	No. of observations available	70	68	60	55	48	43	43
		Number of patients — no.	61	56	49	40	34	33	31
Respiratory rate >20 per minute	Epidural analgesia and usual care	No of available data (n)	63	61	61	67	53	50	50
		Number of patients — no.	50	50	49	43	40	38	36
	Usual care alone	No of available data (n)	69	67	58	54	46	44	41
		Present (n)	53	47	42	37	31	29	28
White blood cell count >12G/L or <4G/L	Epidural analgesia and usual care	No of available data (n)	62	63	59	58	50	49	44
		Number of patients — no.	35	29	32	31	30	36	31
	Usual care alone	No of available data (n)	68	67	68	64	47	43	41
		Number of patients — no.	42	44	37	35	28	31	28

Supplementary Table 13. Sequential organ failure assessment (SOFA) score during the first seven days after randomization.									
			Day 1	Day 2	Day 3	Day 4	Day 5	Day 6	Day 7
Total SOFA score	Epidural analgesia and usual care	No. of observations available	65	65	62	58	56	53	58
		Median [interquartile range]	4 [1–6]	4 [1–5]	4 [1–5]	4 [0–4]	3 [0–5]	3 [0–5]	2 [0–4]
	Usual care alone	No. of observations available	70	69	61	56	52	50	54
		Median [interquartile range]	5 [1–7]	4 [1–6]	4 [0–4]	4 [0–5]	3 [0–5]	3 [0–4]	2 [0–3]
Respiratory SOFA score	Epidural analgesia and usual care	No. of observations available	64	65	62	58	56	53	58
		Median [interquartile range]	1 [0–2]	1 [0–2]	1 [0–2]	1 [0–2]	1 [0–2]	1 [0–2]	1 [0–2]
	Usual care alone	No. of observations available	70	69	61	56	52	50	54
		Median [interquartile range]	1 [0–2]	1 [0–2]	1 [0–2]	2 [0–3]	1 [0–2]	1 [0–2]	1 [0–2]
Cardiovascular SOFA score	Epidural analgesia and usual care	No. of observations available	65	65	62	58	56	53	58
		Median [interquartile range]	1 [0–3]	1 [1–1]	1 [0–1]	1 [0–1]	1 [0–1]	1 [0–1]	1 [0–0]
	Usual care alone	No. of observations available	70	69	61	56	52	50	54
		Median [interquartile range]	0 [0–1]	1 [0–0]	1 [0–0]	1 [0–0]	1 [0–0]	0 [0–0]	0 [0–0]
Hepatic SOFA score	Epidural analgesia and usual care	No. of observations available	62	59	58	54	51	44	47
		Median [interquartile range]	1 [0–1]	0 [0–1]	0 [0–1]	0 [0–0]	0 [0–0]	0 [0–0]	0 [0–0]
	Usual care alone	No. of observations available	66	60	53	46	41	36	36
		Median [interquartile range]	1 [0–1]	1 [0–1]	1 [0–1]	1 [0–1]	1 [0–1]	0 [0–1]	0 [0–1]
Coagulation SOFA score	Epidural analgesia and usual care	No. of observations available	61	61	60	57	51	48	45
		Median [interquartile range]	1 [0–1]	1 [0–1]	1 [0–1]	1 [0–1]	0 [0–0]	0 [0–0]	0 [0–0]
	Usual care alone	No. of observations available	69	68	60	54	47	43	41
		Median [interquartile range]	1 [0–1]	1 [0–1]	1 [0–1]	0 [0–0]	0 [0–0]	0 [0–0]	0 [0–0]
Neurological SOFA score	Epidural analgesia and usual care	No. of observations available	61	57	57	52	50	45	44
		Median [interquartile range]	0 [0–0]	0 [0–0]	0 [0–0]	0 [0–0]	0 [0–0]	0 [0–0]	0 [0–0]
	Usual care alone	No. of observations available	68	66	58	51	45	43	40
		Median [interquartile range]	0 [0-0]	0 [0–0]	0 [0–0]	0 [0–0]	0 [0–0]	0 [0–0]	0 [0–0]
Renal SOFA score	Epidural analgesia and usual care	No. of observations available	65	63	61	57	55	49	49
		Median [interquartile range]	1 [0–1]	1 [0–0]	0 [0–0]	0 [0–0]	0 [0–0]	0 [0–0]	0 [0–0]
	Usual care alone	No. of observations available	69	67	59	54	46	43	41
		Median [interquartile range]	1 [0–1]	1 [0–0]	0 [0–0]	0 [0–0]	0 [0–0]	0 [0–0]	0 [0–0]

Supplementary Table 14. Digestive and nutritional parameters during the first seven days after randomization.									
			Day 1	Day 2	Day 3	Day 4	Day 5	Day 6	Day 7
Nausea	Epidural analgesia and usual care	No. of observations available	64	64	60	55	54	50	49
		Number of patients — no.	5	7	4	2	4	4	2
	Usual care alone	No. of observations available	69	66	58	52	47	46	43
		Number of patients — no.	2	3	3	3	2	2	3
Vomiting	Epidural analgesia and usual care	No. of observations available	65	63	62	57	56	51	50
		Number of patients — no.	5	5	2	1	2	2	3
	Usual care alone	No. of observations available	70	66	58	54	48	45	42
		Number of patients — no.	4	2	2	4	4	0	2
Diarrhea	Epidural analgesia and usual care	No. of observations available	65	63	62	57	56	51	50
		Number of patients — no.	6	8	5	5	6	3	5
	Usual care alone	No. of observations available	67	68	59	54	48	46	43
		Number of patients — no.	6	6	6	10	6	7	6
Ileus	Epidural analgesia and usual care	No. of observations available	65	64	61	57	55	49	50
		Number of patients — no.	20	22	23	15	14	13	8
	Usual care alone	No. of observations available	69	68	60	54	49	46	42
		Number of patients — no.	16	18	18	13	9	8	9
Prokinetics use	Epidural analgesia and usual care	No. of observations available	65	65	52	58	56	53	58
		Erythromycin — no.	15	16	18	17	15	15	18
		Domperidone — no.	7	8	10	9	9	8	9
		Trimebutine — no.	15	21	20	22	22	18	16
		Neostigmine — no.	0	3	6	6	7	3	6
		Other — no.	1	1	2	3	2	1	2
	Usual care alone	No. of observations available	70	69	61	56	52	50	54
		Erythromycin — no.	17	18	18	18	15	13	11
		Domperidone — no.	2	3	4	6	3	4	4
		Trimebutine — no.	14	20	18	18	13	14	16
		Neostigmine — no.	2	2	5	4	4	5	6
		Other — no.	2	2	5	4	2	3	3
Type of caloric intake	Epidural analgesia and usual care	No. of observations available	65	65	52	58	56	53	58
		Intravenous glucose solution — no.	39	33	25	21	16	15	13
		Oral nutrition — no.	20	21	23	23	23	18	21
		Parenteral nutrition — no.	12	15	23	21	22	20	17
		Enteral nutrition — no.	7	13	13	13	14	14	15
		Gastric feeding	7	12	12	12	12	13	11
		Jejunal feeding	0	1	1	1	2	1	4
	Usual care alone	No. of observations available	70	69	61	56	52	50	54
		Intravenous glucose solution — no.	45	37	28	24	19	14	13
		Oral nutrition — no.	20	21	24	20	18	21	20
		Parenteral nutrition — no.	15	15	15	14	12	15	11
		Enteral nutrition — no.	15	17	20	18	18	17	15
		Gastric feeding	14	16	20	18	18	16	14
		Jejunal feeding	0	1	0	0	0	1	1
Total caloric intake	Epidural analgesia and usual care	No. of observations available	40	40	40	36	35	34	30
		Median [interquartile range] — kcal/24h	893 [242–1506]	1101 [410–1830]	1247 [580–1780]	1340 [1000–1945]	1486 [1000–1906]	1438 [1000–1742]	1425 [1000–1742]
	Usual care alone	No. of observations available	38	34	35	30	31	30	29
		Median [interquartile range] — kcal/24h	901 [300–1,500]	1,016 [357–1,510]	1,126 [411–1,600]	1,148 [450–1,650]	1,603 [760–1,800]	1,373 [1,155–1,650]	1,775 [1,350–1,800]
Caloric intake through the enteral route	Epidural analgesia and usual care	No. of observations available	11	18	21	21	21	18	19
		Median [interquartile range] — kcal/24h	666 [80–1,200]	710 [111–1,000]	756 [220–1,007]	698 [126–1,000]	831 [300–1,280]	926 [400–1,330]	794 [321–1,007]
	Usual care alone	No. of observations available	15	16	22	19	20	22	21
		Median [interquartile range] — kcal/24h	662 [200–996]	884 [238–1,470]	734 [90–1,176]	775 [50–1,260]	1028 [211–1,806]	896 [480–1,500]	921 [440–1,500]
Intra-abdominal pressure	Epidural analgesia and usual care	No. of observations available	9	8	6	6	6	8	3
		Median [interquartile range] — cmH_2O	15 [12–20]	16 [14–18]	15 [14–16]	16 [15–17]	16 [16–20]	24 [15–33]	17 [15–19]

	Usual care alone	No. of observations available	12	11	9	7	6	5	3
		Median [interquartile range] — cmH_2O	18 [16–23]	18 [11–26]	17 [14–22]	15 [11–18]	18 [18–19]	15 [11–16]	13 [11–15]

Supplementary Table 15. Use of anticoagulant therapy and antiplatelet drugs during the first seven days after randomization.									
			**Day 1**	**Day 2**	**Day 3**	**Day 4**	**Day 5**	**Day 6**	**Day 7**
Anticoagulant therapy	Epidural analgesia and usual care	None — no.	16	12	11	9	8	8	6
		Preventive — no.	44	46	43	41	42	35	36
		Curative — no.	3	5	5	5	5	7	6
	Usual care alone	None — no.	15	12	5	6	8	7	7
		Preventive — no.	47	48	48	43	35	33	30
		Curative — no.	7	6	7	6	6	6	5
Antiplatelet drugs	Epidural analgesia and usual care	None — no.	54	56	52	48	48	44	43
		Salicylic acid — no.	9	8	8	8	8	7	7
		Clopidogrel — no.	0	0	0	0	0	0	0
		Salicylic acid and clopidogrel — no.	0	0	0	0	0	0	0
		Other — no.	1	0	0	0	0	0	0
	Usual care alone	None — no.	64	61	56	51	47	43	40
		Salicylic acid — no.	6	5	4	3	2	2	2
		Clopidogrel — no.	0	0	0	0	0	0	0
		Salicylic acid and clopidogrel — no.	0	0	0	0	0	0	0
		Other — no.	0	0	0	0	0	0	0

Supplementary Table 16. Routine laboratory results during the first seven days after randomization.									
			Day 1	Day 2	Day 3	Day 4	Day 5	Day 6	Day 7
Blood glucose — mmol/L	Epidural analgesia and usual care	Median value [interquartile range]	1.1 [1.0−1.3]	1.2 [1.0−1.4]	1.1 [1.0−1.3]	1.1 [1.0−1.5]	1.1 [1.0−1.3]	1.2 [1.0−1.6]	1.1 [1.0−1.5]
	Usual care alone	Median value [interquartile range]	1.3 [1.0−1.6]	1.1 [1.0−1.3]	1.2 [1.0−1.5]	1.2 [1.0−1.6]	1.3 [1.0−1.7]	1.1 [1.0−1.5]	1.2 [1.0−1.6]
Serum lipase — IU/L	Epidural analgesia and usual care	Median value [interquartile range]	1,308 [64−829]	366 [56−471]	171 [37−161]	162 [31−215]	1,308 [64−829]	366 [56−471]	171 [37−161]
	Usual care alone	Median value [interquartile range]	635 [103−775]	302 [46−251]	189 [39−203]	146 [31−209]	635 [103−775]	302 [46−251]	189 [39−203]
Serum aspartate aminotransferase — IU/L	Epidural analgesia and usual care	Median value [interquartile range]	527 [28−98]	124 [25−78]	107 [30−66]	53 [28−60]	527 [28−98]	124 [25−78]	107 [30−66]
	Usual care alone	Median value [interquartile range]	113 [27−72]	274 [24−72]	268 [25−57]	340 [27−55]	11 [27−72]	274 [24−72]	268 [25−57]
Serum alanine aminotransferase — IU/L	Epidural analgesia and usual care	Median value [interquartile range]	252 [26−83]	97 [26−74]	93 [27−59]	47 [28−52]	252 [26−83]	97 [26−74]	93 [27−59]
	Usual care alone	Median value [interquartile range]	108 [23−72]	104 [22−62]	122 [19−69]	202 [21−59]	108 [23−72]	104 [22−62]	122 [19−69]
Serum alkaline phosphatase — IU/L	Epidural analgesia and usual care	Median value [interquartile range]	121 [64−124]	118 [68−134]	122 [78−143]	128 [89−156]	121 [64−124]	118 [68−134]	122 [78−143]
	Usual care alone	Median value [interquartile range]	106 [61−129]	109 [65−137]	11 [69−158]	143 [86-180]	106 [61−129]	10 [65−137]	118 [69−158]
GGT — IU/L	Epidural analgesia and usual care	Median value [interquartile range]	221 [58−260]	192 [76−238]	177 [75−214]	190 [96−252]	221 [58−260]	192 [76−238]	177 [75−214]
	Usual care alone	Median value [interquartile range]	202 [67−274]	192 [61−255]	172 [60−236]	202 [92−311]	202 [67−274]	192 [61−255]	172 [60−236]
Serum bilirubin — IU/L	Epidural analgesia and usual care	Median value [interquartile range]	24 [11−29]	19 [11−22]	17 [9−21]	16 [10−17]	24 [11−29]	19 [11−22]	17 [9−21]
	Usual care alone	Median value [interquartile range]	24 [13−29]	21 [9−30]	21 [10−27]	21 [10−31]	24 [13−29]	21 [9−30]	21 [10−27]
Serum lactate dehydrogenase — IU/L	Epidural analgesia and usual care	≤350 IU/L — no.<br>>350 IU/L — no.<br>Unknown — no.	4<br>15<br>43	7<br>11<br>41	7<br>13<br>42	7<br>9<br>40	4<br>15<br>43	7<br>11<br>41	7<br>13<br>42
	Usual care alone	≤350 IU/L — no.<br>>350 IU/L — no.<br>Unknown — no.	11<br>15<br>34	15<br>10<br>38	8<br>15<br>31	7<br>13<br>30	11<br>15<br>34	15<br>10<br>38	8<br>15<br>31
Prothrombin rate — %	Epidural analgesia and usual care	Median value [interquartile range]	70 [61−86]	73 [59−89]	77 [69−89]	79 [71−89]	7 [61−86]	73 [59−89]	77 [69−89]
	Usual care alone	Median value [interquartile range]	73 [65−92]	75 [67−90]	79 [74−91]	78 [74−90]	73 [65-92]	75 [67−90]	79 [74−91]
Activated cephalin time — seconds	Epidural analgesia and usual care	Median value [interquartile range]	42 [30−46]	42 [33−47]	39 [33−43]	39 [33−44]	42 [30−46]	42 [33−47]	39 [33−43]
	Usual care alone	Median value [interquartile range]	44 [31−47]	43 [33−47]	40 [34−46]	38 [33−45]	44 [31−47]	43 [33−47]	40 [34−46]
Serum C-reactive protein — mg/L	Epidural analgesia and usual care	Median value [interquartile range]	248 [171−309]	267 [210−347]	241 [166−309]	219 [165−277]	248 [171−309]	267 [210−347]	241 [166−309]
	Usual care alone	Median value [interquartile range]	243 [131−339]	257 [166−351]	256 [176−321]	239 [131−323]	243 [131−339]	257 [166−351]	256 [176−321]

IU: international unit.

Supplementary Table 17. Deaths and their causes during the first seven days after randomization.								
		**Day 1**	**Day 2**	**Day 3**	**Day 4**	**Day 5**	**Day 6**	**Day 7**
Epidural analgesia and usual care	No. of observations available	65	63	62	58	55	51	50
	Death — no.	1	0	1	0	0	0	0
	Cause(s) of death	Multiple organ failure	--	Septic shock	--	--	--	--
Usual care alone	No. of observations available	67	65	60	54	48	48	43
	Death — no.	3	2	0	1	1	0	1
	Cause(s) of death	Hemorrhagic shock<br>Septic shock<br>Unknown	Septic shock	--	Septic shock	--	--	Septic shock

Supplementary Table 18. Analysis of the primary outcome and post-hoc zero-inflated negative binomial regression in the intention-to-treat population.*

	**Univariate analysis** (Mann-Whitney U test)		**Univariate analysis** (Zero-inflated negative binomial regression)				**Multivariable analysis** (Zero-inflated negative binomial regression)	
	**Ventilator-free days at day 30, median [IQR]**	***p*-value**	**Patients with ventilator-free days of 30 — no. (%)**	**Odds ratio for having 30 ventilator-free days [95% CI], *p*-value**	**Number of ventilator-free days if not 30, median [IQR]**	**Incident rate ratio for the number of ventilator-free days when not equal to 30 [95% CI], *p*-value**	**Odds ratio for having 30 ventilator-free days [95% CI], *p*-value**	**Incident rate ratio for the number of ventilator-free days when not equal to 30 [95% CI], *p*-value**
Randomization group		0.59						
Control	30 [18−30] (n=70)		42 (60)	*reference*	14 [0−25] (n=28)	*reference*	*reference*	*reference*
Intervention	30 [15−30] (n=65)		37 (57)	0.88 [0.44−1.92] *p* = 0.72	7 [0−21] (n=28)	0.88 [0.60−1.29] *p* = 0.52	1.03 [0.53−1.99] *p* = 0.94	0.95 [0.50−1.83] *p* = 0.89
Marshall score strata at baseline†		<10^{-3}						
0	30 [30−30] (n=31)		24 (77)	*reference*	20 [6−27] (n=7)	*reference*	*reference*	*reference*
1 or 2	30 [26−30] (n=68)		46 (68)	0.62 [0.23−1.65] *p* = 0.34	11 [0−25] (n=22)	0.69 [0.37−1.28] p=0.24	0.58 [0.20−1.64] p=0.30	0.66 [0.20−2.16] p=0.50
3 or 4	17 [8−28] (n=36)		9 (25)	0.10 [0.03−0.15] *p* <0.001	8 [0−18] (n=27)	0.61 [0.34−1.12] *p* = 0.11	0.11 [0.03−0.40] *p* <0.001	0.56 [0.20−1.51] *p* = 0.25
Duration from first symptoms to randomization (days)		0.94						
<48h	30 [12−30] (n=60)		36 (60)	*reference*	4 [0−19] (n=24)	*reference*	*reference*	*reference*
≥48h	30 [18−30] (n=75)		43 (57)	0.89 [0.44−1.79] *p* = 0.74	17 [0−25] (n=32)	1.27 [0.87−1.86] *p* = 0.22	1.02 [0.52−1.99] *p* = 0.96	1.47 [0.62−3.48] *p* = 0.38

* The primary endpoint was the number of ventilator-free days as calculated from randomization to day 30. All the patients who had died by day 30 were considered to have had no ventilator-free days. A univariate analysis of randomization-stratification variables is also provided. The analysis was adjusted for the randomization-stratification variables including site as random effect. Number of complete cases for multivariable analysis: 135 patients.

† Three strata of increasing severity were defined according to the maximum modified Marshall score obtained for at least one of the respiratory, renal, or hemodynamic functions (0, 1–2, and 3–4).

CI: confidence interval; IQR: interquartile range.

Supplementary Table 19. Analysis of the primary outcome restricted to the per-protocol population.*

	**Univariate analysis** (Mann-Whitney U test or correlation coefficient)		**Univariate analysis** (Zero-inflated negative binomial regression)				**Multivariable analysis** (Zero-inflated negative binomial regression)	
	**Ventilator-free days at day 30, median [IQR]**	***p*-value**	**Patients with ventilator-free days of 30 — no. (%)**	**Odds ratio for having 30 ventilator-free days [95% CI], *p*-value**	**Number of ventilator-free days if not 30, median [IQR]**	**Incident rate ratio for the number of ventilator-free days when not equal to 30 [95% CI], *p*-value**	**Odds ratio for having 30 ventilator-free days [95% CI], *p*-value**	**Incident rate ratio for the number of ventilator-free days when not equal to 30 [95% CI], *p*-value**
Randomization group		0.59						
Control	30 [18−30] (n=70)		42 (60)	*reference*	14 [0−25] (n=28)	*reference*	*reference*	*reference*
Intervention	30 [15−30] (n=65)		37 (57)	0.88 [0.44−1.92] *p* = 0.72	7 [0−21] (n=28)	0.88 [0.60−1.29] *p* = 0.52	1.03 [0.53−1.99] *p* = 0.94	0.95 [0.50−1.83] *p* = 0.89
Marshall score strata at baseline†		<0.001						
0	30 [30−30] (n=30)		23 (77)	*reference*	20 [6−27] (n=7)	*reference*	*reference*	*reference*
1 or 2	30 [25−30] (n=67)		45 (67)	0.63 [0.23−1.70] p=0.36	11 [0−25] (n=22)	0.69 [0.70−1.28] p=0.24	0.61 [0.22−1.69] p=0.34	0.60 [0.31−1.15] p=0.12
3 or 4	16 [0−26] (n=33)		8 (24)	0.10 [0.03−0.31] *p* <0.001	8 [0−18] (n=25)	0.61 [0.33−1.14] *p* = 0.12	0.09 [0.03−0.31] *p* <0.001	0.55 [0.29−1.03] *p* = 0.06
Duration from first symptoms to randomization (days)		0.96						
<48h	30 [12−30] (n=58)		35 (60)	*reference*	5 [0−20] (n=23)	*reference*	*reference*	*reference*
≥48h	30 [18−30] (n=72)		41 (57)	0.86 [0.43−1.75] *p* = 0.68	16 [0−25] (n=31)	1.23 [0.84−1.84] *p* = 0.29	1.12 [0.50−2.50] *p* = 0.78	1.36 [0.91−2.03] *p* = 0.14

* The primary outcome was the number of ventilator-free days as calculated from randomization to day 30. All the patients who had died by day 30 were considered to have had no ventilator-free days. Univariate analysis of randomization-stratification variables is also provided. The analysis was adjusted for the randomization-stratification variables including site as random effect.

† Three strata of increasing severity were defined according to the maximum modified Marshall score obtained for at least one of the respiratory, renal, or hemodynamic functions (0, 1–2, and 3–4).

CI: confidence interval; IQR: interquartile range.

**Supplementary Table 20. Post-hoc unadjusted sensitivity analysis of the primary outcome and zero-inflated negative binomial regression in the intention-to-treat population, as reported for each quartile of the SOFA score at baseline.***						
	**Mann-Whitney U test**		**Zero-inflated negative binomial regression**			
	**Ventilator-free days at day 30, median [IQR]**	***p*-value**	**Patients with ventilator-free days of 30 — no. (%)**	**Odds ratio for having 30 ventilator-free days [95% CI], *p*-value**	**Number of ventilator-free days if not 30, median [IQR]**	**Incident rate ratio for the number of ventilator-free days when not equal to 30 [95% CI], *p*-value**
**Baseline SOFA ≤2 (quartile 1)**		0.44				
Randomization group						
Control	30 [30–30] (n=32)		28 (88)	*reference*	26 [15–28] (n=4)	*reference*
Intervention	30 [30–30] (n=20)		16 (80)	1.68 [0.34–8.33] *p* = 0.52	19 [12–25] (n=4)	0.58 [0.13–3.67] *p* = 0.67
**Baseline 2< SOFA ≤3 (quartile 2)**		0.09				
Randomization group						
Control	28 [25–30] (n=11)		5 (45)	*reference*	26 [24–28] (n=6)	*reference*
Intervention	30 [30–30] (n=9)		8 (89)	0.11 [0.01–1.32] *p* = 0.08	27 [27–27] (n=1)	0.55 [0.11–2.77] *p* = 0.47
**Baseline 3< SOFA ≤6 (quartile 3)**		0.22				
Randomization group						
Control	26 [12–30] (n=10)		4 (40)	*reference*	15 [11–24] (n=6)	*reference*
Intervention	30 [24–30] (n=20)		13 (65)	0.35 [0.07–1.73] *p* = 0.20	23 [0–25] (n=7)	0.98 [0.43–2.20] *p* = 0.95
**Baseline SOFA ≥6 (quartile 4)**		0.01				
Randomization group						
Control	27 [18–30] (n=17)		5 (29)	*reference*	22 [17–27] (n=12)	*reference*
Intervention	14 [1–24] (n=16)		1 (6)	6.75 [0.45–102.51] *p* = 0.17	11 [0–22] (n=15)	0.52 [0.29–0.95] *p* = 0.03

* The primary outcome was the number of ventilator-free days as calculated from randomization to day 30. All the patients who had died by day 30 were considered to have had no ventilator-free days. Univariate analysis of randomization-stratification variables is also provided. The analysis was adjusted for the randomization-stratification variables including site as random effect.
SOFA: sequential organ failure assessment; CI: confidence interval; IQR: interquartile range.
