# Supplementary material for: Thoracic epidural analgesia in intensive care unit patients with acute pancreatitis: the EPIPAN multicenter randomized controlled trial
Source: Crit Care. 2023 May 31;27:213. doi: 10.1186/s13054-023-04502-w (PMC10230742; doi:10.1186/s13054-023-04502-w)
Supplement: Supplementary file 2 — Additional file 2. Research protocols and analysis plans. [file 13054_2023_4502_MOESM2_ESM.pdf]

Additional File 2

Trial Protocols and Statistical Analysis Plans

Thoracic epidural analgesia in intensive care unit patients with acute pancreatitis: the EPIPAN multicenter randomized controlled trial

This supplement contains the following items:

1. **STUDY PROTOCOL**

    Original Study Protocol

    Final Study Protocol

    Summary of changes to the Study Protocol

2. **STATISTICAL ANALYSIS PLAN**

    Original Statistical Analysis Plan

    Final (Modified) Statistical Analysis Plan

    Summary of changes to the Statistical Analysis Plan

1. STUDY PROTOCOL

- Original Study Protocol (version 1; November 7, 2013)

- Final Study Protocol (version 8; July 3, 2018)

- Summary of changes to the Study Protocol

Original Study Protocol (version 1; November 7, 2013)

EPIDURAL ANALGESIA FOR ACUTE PANCREATITIS IN THE INTENSIVE CARE UNIT: A MULTICENTER RANDOMIZED CONTROLLED TRIAL

Short title: EPIPAN study (*Epidural Analgesia and Pancreatitis*)

Version : 1 *(English)* **Date: November 07, 2013**

Sponsor Code	EudraCT or AFSSAPS number
**RBHP 2013 JABAUDON**	**2013-004652-37**

Sponsor

C.H.U. de Clermont-Ferrand
58 Rue de Montalembert
63003 Clermont-Ferrand Cedex 1

Principal Investigator

Dr JABAUDON Matthieu
Réanimation Adultes et Unité de Soins Continus
R2D2 – EA 7281, Université d'Auvergne, Faculté de Médecine
CHU Estaing, CHU Clermont-Ferrand
1 place Lucie Aubrac 63003 Clermont-Ferrand cedex 1
Mail : mjabaudon@chu-clermontferrand.fr
Tél : 04 73 75 05 01 / Fax : 04 73 75 05 00

Methodologist

Bruno PEREIRA
Délégation Recherche Clinique & Innovation, CHU Clermont-Ferrand - Villa annexe IFSI
58, Rue Montalembert
63003 Clermont-Ferrand cedex
Mail : bpereira@chu-clermontferrand.fr

Participating centers

CHU Estaing, CHU Clermont-Ferrand,
1 place Lucie Aubrac, 63003 Clermont-Ferrand cedex 1

CHU Gabriel Montpied, CHU Clermont-Ferrand,
58 Rue de Montalembert, 63003 Clermont-Ferrand Cedex 1

Hôpitaux Universitaires de Genève,
4, rue Gabrielle-Perret-Gentil, CH-1211 Genève 14

Hôpital Saint Eloi, CHU de Montpellier,
80 avenue Augustin Fliche, 34295 Montpellier cedex 5

Groupe Hospitalo Universitaire Carémeau - CHU Nîmes,
Place du Pr Debré, 30029 Nimes cedex

Centre Hospitalier Emile Roux, Le Puy en Velay
BP 352, 43012 Le Puy en Velay

Centre Hospitalier de Cannes,
15 avenue des Broussailles – CS 50008, 06414 Cannes Cedex

SUMMARY

Context: Severe acute pancreatitis is responsible for a high mortality of about 20%. The use of mechanical ventilation for acute respiratory failure is in itself a criterion of severity, and is associated with a significant increase in morbidity and mortality and in health care-related costs. The management of severe acute pancreatitis mainly includes early enteral nutrition, fluid and electrolyte resuscitation, and adequate management of pain and of complications. Recent evidence suggests that epidural analgesia may reduce postoperative respiratory complications after major thoracoabdominal surgery and facilitate postoperative rehabilitation. Numerous preclinical studies also suggest that epidural analgesia may have an anti-inflammatory effect and a beneficial effect on splanchnic perfusion or respiratory function.
To date, epidural analgesia has not been investigated in intensive care unit patients with acute pancreatitis, with regards to clinical outcome.

Objectives: The objective of our study is to test the effect of epidural analgesia on lung dysfunction in intensive care unit patients with acute pancreatitis, as we hypothesize that it could limit lung failure requiring invasive mechanical ventilation or the duration of invasive mechanical ventilation.

Type of study: Prospective, randomized, controlled, multicenter, parallel group trial.

Number of participating centers: 7 intensive care units among 6 hospitals in France, Belgium, and Switzerland.

Study description: Prospective, randomized, controlled, multicenter, clinical trial in the intensive care unit. After enrolment, patients will be randomized into two groups: a control group in which available guidelines on analgesia will be applied, and an intervention group in which patients receive thoracic epidural analgesia for at least 3 days. Beyond the analgesic strategy, recent consensual guidelines on the management of acute pancreatitis will be applied.

Primary endpoint measure: The number of days free from invasive mechanical ventilation at day 30 after randomization (VFD30), defined as the number of days from randomization to day 30 on which a patient is able to breathe without invasive assistance. A difference in ventilator-free days can reflect a difference in mortality, ventilator days among survivors, or both.

Number of subjects: To demonstrate an increase in VFD30 from 13 to 20 days between the control group and the intervention group, respectively, a total of 74 patients will be needed in each study arm, with a power of 80% and a bilateral alpha risk of 5%.

Inclusion criteria: Adult patients admitted to the intensive care unit with severe acute pancreatitis.

Exclusion Criteria: Absolute contra-indication for thoracic epidural catheter placement (Prothrombin time <60%, Platelet count < 75G/L, curative anticoagulant therapy with heparin interrupted for less than 8 hours, local infection, active central nervous system infection, history of back surgery associated with a dural space procedure, suspected or confirmed intracranial hypertension); Refractory circulatory shock despite appropriate resuscitation; Known allergy to ropivacaine, sufentanil or clonidine; Age under 18 or under tutelage measures (*curatelle*, *tutelle*); Absence of coverage by the French health insurance system (*sécurité sociale*).

Brief Description of the intervention: Thoracic epidural analgesia will be performed using combined administration of ropivacaine (2 mg/ml) and sufentanil (0.5 µg/ml) through patient-controlled epidural analgesia (PCEA). PCEA parameters will be fixed as follows:

continuous administration of 5 to 15 ml/h and bolus of 3 to 10 ml every 10 minutes. Iterative epidural administration of clonidine (1 µg/kg) will be allowed to achieve analgesia goals.
In both groups, conventional analgesia will include enteral and/or parenteral administration analgesics, ranging from step 1 to step 3 drugs according to the WHO analgesics ladder (including acetaminophen, nefopam, tramadol, opioids). The route, dose and frequency of analgesics administrations will be based on current protocols from participating intensive care units.
Beyond the analgesic strategy, recent consensual guidelines on the management of acute pancreatitis will be applied.

Course of the study: Patients will be informed when admitted to the intensive care unit, and enrolled and randomized after consent has been obtained. Web-based randomization will be performed by minimization, considering stratifications by: center, the cause of acute pancreatitis (biliary, alcohol, other), and by the duration since first onset of abdominal pain (less or more than 48 hours). The intervention (epidural analgesia) will be administered for a minimum duration of 72 hours. Apart from the modalities of multimodal analgesia, the medical and paramedical management will be based on current guidelines and current protocols from participating centers. Patients will be followed-up until day 31 after randomization, with visits at days 1, 2, 3, 7, 15, and 30.

Study duration: 36 months; Duration of patient participation in the study: 31 days.

Table of Content

General Information **8**
Title of the research study 8
Sponsor code 8
Sponsor 8
Study coordination 8
Investigators 8
Associated partners 10
Places where the research is performed is carried out 11
Data processing 12
Ethics committee (Comité de Protection des Personnes) 12
Estimated schedule of the study 12

Study Rationale / Scientific Justification of the Research **13**
Background and rationale 13
Study objectives 14
Summary of known and foreseeable benefits and risks to research subjects 14
Expected impact 14
References to scientific literature and relevant data used as a reference for research 14

Study Objectives **16**
Main objective 16
Secondary Objectives 16

Study Description **17**
Study design 17
Research category 17

Study Population **17**
Inclusion criteria 17
Non Inclusion criteria 17
Procedure for premature termination of research 17
Exclusion period and participation in another research 18
Volunteer compensation 18
Recruitment modalities 18

Study Methodology **18**
Study outcomes 18
Description of Research Methodology 19
Description of measures taken to reduce and avoid bias 19

Practical implementation of the protocol **19**
Description of the study procedures performed (description of each visit) / products used in the course of the research 19

Description of the general logistical organisation of the trial 20
Biological sampling and analysis for the study 22
Différence par rapport à la prise en charge de routine. 23
Expected duration of participation of individuals and description of the trial chronology 23

Intervention (drug / medical device / other) under investigation 23
Description of the treatment 23
Dosage, method of administration and duration of treatment 24
Presentation of the drugs 24
Issuance and compliance 25
Drugs and treatments allowed or not allowed during the trial 26

Collected data 26

Statistical Methods 27
Sample size estimation 27
Statistical analysis 27
Data management and analysis 28

Safety assessment – Management of adverse events 28
Definitions 28
Serious adverse event reporting 29
Independent data monitoring and safety committee (DMSC) 30
End of the study / Study interruption 31
Follow-up of subjects with an adverse event 31

Right of access to source document and data 31
Access to data 31
Source data 31
Data confidentiality 31
Registration in the national file of biomedical research subjects 32

Quality control and assurance 32
Engagement of the investigators and the sponsor of the study 32
Quality assurance 32
Quality control 32
Case report form 33

Ethical considerations 33
Ethics Committee and Medicine Agency 33
Information for patients and written informed consent form 33
Protocol amendments 34
Management of the patient enrolled in the study 34

Data processing and storage of study documents 34
Data entry and processing 34

CNIL 34
Data retention and archiving 35

Funding and insurance 35
Estimated cost of the study 35
Study insurance 36

Communication - Rules for publication 36

Feasibility 36
Competence of the teams involved 36
Recruitment feasibility 36

Annexes 37

1. General Information

1.1. Title of the research study

EPIDURAL ANALGESIA FOR ACUTE PANCREATITIS IN THE INTENSIVE CARE UNIT: A MULTICENTER RANDOMIZED CONTROLLED TRIAL

Short title: *Epidural Analgesia in Acute Pancreatitis (the EPIPAN study)*
ANSM registration number: **2013-004652-37**
Version n°1 - November 07, 2013

1.2. Sponsor code

RBHP 2013 JABAUDON

1.3. Sponsor

CHU Clermont-Ferrand
58 rue Montalembert
63003 Clermont-Ferrand cedex 1
France

Direction Générale Adjointe – Délégation Régionale à la Recherche Clinique
Tél : 04.73.751.195 / Fax : 04.73.754.730

1.4. Study coordination

Dr JABAUDON Matthieu
Department of Perioperative Medicine, CHU Clermont-Ferrand
GReD, Université Clermont Auvergne, CNRS UMR 6293, INSERM U1103
Clermont-Ferrand, France
Tél : 04 73 75 05 01 / Fax : 04 73 75 05 00 / mjabaudon@chu-clermontferrand.fr

Délégation à la Recherche Clinique et à l'Innovation, CHU Clermont-Ferrand
58 rue Montalembert, 63003 Clermont-Ferrand cedex 1
Tél : 04 73 75 11 95 / Fax : 04 73 75 47 30

1.5. Investigators

1.5.1. *Coordinating investigator*

Dr JABAUDON Matthieu
Department of Perioperative Medicine, CHU Clermont-Ferrand
R2D2 – EA 7281, Université d'Auvergne, Faculté de Médecine
CHU Clermont-Ferrand, 1 place Lucie Aubrac
63003 Clermont-Ferrand cedex 1, France
Tél : 04 73 75 05 01 / Fax : 04 73 75 05 00 / Mail : mjabaudon@chu-clermontferrand.fr

1.5.2. *Co-investigators*

Pr CONSTANTIN Jean-Michel
Department of Perioperative Medicine, CHU Clermont-Ferrand
R2D2 – EA 7281, Université d'Auvergne, Faculté de Médecine
CHU Estaing, CHU Clermont-Ferrand, 1 place Lucie Aubrac
63003 Clermont-Ferrand cedex 1, France
Mail : jmconstantin@chu-clermontferrand.fr

Pr BÜHLER Leo
Service de Chirurgie viscérale et transplantation, Unité d'Investigations Chirurgicales
Hôpitaux Universitaires de Genève
4, rue Gabrielle-Perret-Gentil, CH-1211 Genève 14, Suisse
leo.Buhler@hcuge.ch

Pr JABER Samir
Département d'Anesthésie et Réanimation B (SAR B)
Hôpital Saint-Eloi, CHU de Montpellier, France
Mail : s-jaber@chu-montpellier.fr

Pr LEFRANT Jean-Yves
Service Anesthésie-Réanimation
Hôpital Carémeau, CHU Nîmes, France
Email : jean.yves.lefrant@chu-nimes.fr

Dr CLAUD Bernard
Réanimation et Unité de Soins Continus, Département d'Anesthésie Réanimation
Centre Hospitalier Emile Roux, Le Puy en Velay, France
Mail : bernard.claud@ch-lepuy.fr

Dr CHABANNE Russell
Réanimation polyvalente à orientation neurologique
Hôpital Gabriel Montpied, CHU Clermont-Ferrand, France
Mail : rchabanne@chu-clermontferrand.fr

Dr BERTRAND Pierre-Marie
Réanimation médico-chirurgicale
Centre Hospitalier de Cannes, France
Mail : pm.bertrand@gmail.com

1.6. Associated partners

Dr Bruno PEREIRA (biostatisticien)
Délégation Régionale à la Recherche Clinique
CHU de Clermont-Ferrand

Dr Laurence ROSZYK
R2D2 – EA 7281, Université d'Auvergne, Faculté de Médecine
Laboratoire de Biochimie Médicale et Biologie Moléculaire, CHU Clermont-Ferrand, France

Pr Vincent SAPIN
R2D2 – EA 7281, Université d'Auvergne, Faculté de Médecine
Laboratoire de Biochimie Médicale et Biologie Moléculaire, CHU Clermont-Ferrand, France

Dr Etienne IMHOFF
CHU de Clermont-Ferrand, France

Dr Stéphanie BULYEZ
CHU de Clermont-Ferrand, France

1.7. Places where the research is performed is carried out

CHU Estaing, CHU Clermont-Ferrand
1 place Lucie Aubrac, 63003 Clermont-Ferrand cedex 1
Pr Jean-Michel CONSTANTIN, Réanimation Adultes et Unité de Soins Continus, Service Anesthésie Réanimation
Tél : 04 73 75 05 01 / Fax : 04 73 75 05 00 / jmconstantin@chu-clermontferrand.fr

Hôpitaux Universitaires de Genève
4, rue Gabrielle-Perret-Gentil, CH-1211 Genève 14
Pr Leo BÜHLER, Service de Chirurgie Viscérale et Transplantation
Tél : +41 22 372 77 03 / Fax : +41 22 372 77 03 / leo.Buhler@hcuge.ch

Hôpital Saint Eloi, CHU de Montpellier
80 avenue Augustin Fliche, 34295 Montpellier cedex 5
Pr Samir JABER, Service Anesthésie Réanimation
Tél : 04 67 33 72 71 / Fax : 04 67 33 74 48 / s-jaber@chu-montpellier.fr

Groupe Hospitalo Universitaire Carémeau - CHU Nîmes
Place du Pr Debré, 30029 Nîmes cedex
Pr Jean-Yves LEFRANT, Service Anesthésie Réanimation
Tél : 04 66 68 30 50 / Fax : 04 66 68 38 51 / jean.yves.lefrant@chu-nimes.fr

Centre Hospitalier Emile Roux, Le Puy en Velay

BP 352, 43012 Le Puy en Velay
Dr CLAUD Bernard, Réanimation et Unité de Soins Continus, Département d'Anesthésie Réanimation
Tél : 04 71 04 32 10 / Fax : 04 71 04 33 70 / bernard.claud@ch-lepuy.fr

CHU Gabriel Montpied, CHU Clermont-Ferrand
58 Rue de Montalembert, 63003 Clermont-Ferrand Cedex 1
Dr CHABANNE Russell, Service d'Anesthésie Réanimation
Tél : 04 73 75 16 48 / Fax : 04 74 75 13 31 / rchabanne@chu-clermontferrand.fr

Centre Hospitalier de Cannes
15 avenue des Broussailles – CS 50008, 06414 Cannes Cedex
Dr BERTRAND Pierre-Marie, Service de Réanimation Médico-Chirurgicale
Tél : 04 93 69 70 25 / Fax : 04 93 69 75 77 / pm.bertrand@gmail.com

1.8. Data processing

Department of Perioperative Medicine, CHU Clermont-Ferrand
R2D2 – EA 7281, Universit d'Auvergne, Facult de M decine, Clermont-Ferrand

Délégation Régionale à la Recherche Clinique, CHU Clermont-Ferrand

1.9. Ethics committee (*Comité de Protection des Personnes*)

Comité de Protection des Personnes (CPP) Sud Est VI

1.10. Estimated schedule of the study

- Submission to the sponsor local research committee (COMAP/COMVAL, DRCI, CHU Clermont-Ferrand): august/september 2013
- Submission to the Ethics committee (CPP) : october 2013
- CPP Approval: february 2014
- ANSM Approval: february 2014
- Start of study : february 2014
- Enrolment period: february 2014 - 2017
- Estimated end of study: February 2017
- End of study report : June 2017

2. Study Rationale / Scientific Justification of the Research

2.1. Background and rationale

Acute pancreatitis (AP) is one of the most frequent gastrointestinal diseases, whose incidence in the US reaches 35 per 100,000 population annually. In 2009, AP was responsible for

275,000 hospital admissions in the USA, with a total cost of over US $2,5 billion.[1,2] AP develops when intracellular protective mechanisms to prevent trypsinogen activation or reduce trypsin activity are overwhelmed[3]. The initiating event may be any insult to the acinar cell that impairs the secretion of zymogen granules, such as alcohol abuse or gallstone migration into the common bile duct. Once the process of cellular injury is initiated, cellular membrane trafficking becomes chaotic, leading to the release of proinflammatory mediators (tumour necrosis factor (TNF)-α, interleukin (IL)-6, and IL-8). These mediators participate to an increase in pancreatic vascular permeability that subsequently favours hemorrhage, oedema and eventually pancreatic necrosis. As these mediators are excreted into the circulation, systemic complications can arise, such as bacteraemia due to gut flora translocation, acute respiratory distress syndrome (ARDS)[4], pleural effusions, gastrointestinal hemorrhage and renal failure.[3,5–8]

The revised Atlanta classification addresses the clinical course and severity of the disease.[9] AP may be divided into two forms, interstitial oedematous pancreatitis, during the first week, and necrotising pancreatitis during a later phase (after 7 days). In approximately 80% of patients, the severity of AP is rather mild and resolves without serious morbidity. However, in up to 20% of patients, AP presents in a more severe form requiring admission to the intensive care unit (ICU) due to persistent organ failure.[9,10] Mortality rate can reach 20-40% in severe AP because of multiorgan failure (MOF) and pancreatic necrosis.[1,11]

The amplifying effects of inflammatory and oxidative impairment often lead to severe AP-induced complications, which are often regarded as hallmarks of severe AP and herald poor outcome. In a recent French observational study of ICU patients with severe AP, 58% of patients developed acute respiratory failure requiring intubation and invasive mechanical ventilation (MV) (mean duration 15 days, standard deviation (SD) 17 days), and such patients had higher mortality rates than those who were not intubated (34% vs 1.4%).[11] Since respiratory failure is the main cause of death in patients with severe AP, more work is needed for us to prevent and treat AP-associated respiratory failure. Despite recent substantial improvements in the multidisciplinary management of AP (e.g., with regards to fluid therapy, intensive care management, prevention of infectious complications, nutritional support, biliary tract management or necrotising pancreatitis management), the prognosis of severe AP remains poor in patients who develop acute respiratory failure requiring intubation and invasive respiratory support.[3,9,12] Of notes, available therapeutic approaches do not have a direct action on the pancreas itself but aim to attenuate the process of MOF present in the severe form of AP, and no causal treatment has been developed yet.

Epidural analgesia (EA) is one of the most widely and versatile utilized neural deafferentation techniques. It is used for analgesia during the perioperative period, but also for obstetrics labour and trauma as well as in the treatment of acute, chronic and cancer-related pain.[13,14] Its objective is not only to block noxious afferent stimuli, but also to induce bilateral selective thoracic sympathetic blockade. In addition to analgesia itself, the modulatory effects of thoracic EA could improve organ perfusion with reduced complications in the perioperative period, thus possibly decreasing postoperative complications, shortening hospital stay and improving survival.[14–16]

EA has not yet been extensively assessed in the ICU setting in general, and in critically ill patients with severe AP in particular. Several studies suggest that thoracic EA might be a safe procedure in centers comprising anaesthesiologists with expertise in EA, and thoracic EA has already been used for years to treat pain during AP in critically ill patients in some centers.[17–19] In addition, recent animal studies suggest that thoracic EA may decrease the severity of AP, with reduced respiratory, thromboembolic and abdominal complications.[20–22] EA further decreased the severity of metabolic acidosis and tissue injury in animals, thus preventing the progression from oedematous to necrotising AP.[23] EA may also restore pancreatic hypoperfusion induced by AP through blood flow redistribution from splanchnic to non-perfused pancreatic regions,[24,25] and a recent clinical study suggests that EA could increase pancreatic arterial perfusion and improve clinical outcome in patients with AP.[19] Findings from other experimental studies also support

beneficial effects of EA in severe AP, such as increased gut barrier function and renal perfusion, decreased liver damage and inflammatory response, and reduced mortality.[22,24,26,27]

Despite such promising findings from preclinical studies, the effects of thoracic EA on major clinical outcomes have never been specifically assessed and its benefit in critically ill patients with AP remains uncertain.

2.2. Study objectives

Primary objective: To determine whether the use of thoracic EA combined to standard care is more effective at increasing ventilator-free days (VFD) at day 30 over standard care alone in critically ill patients with AP. The goal of the EPIPAN trial is therefore to test the impact of thoracic EA on respiratory failure, with the hypothesis that EA could influence survival and/or the need for invasive MV and/or its duration when invasive MV is required.

Secondary objectives: To determine whether in comparison to standard care alone, application of thoracic EA combined with standard care could improve survival, decrease major complications of AP (including sepsis, organ failure), AP-related costs, the need for medical, surgical and radiological interventions, and impact biological markers of systemic inflammation, lung injury and renal failure.

2.3. Summary of known and foreseeable benefits and risks to research subjects

Benefits: No direct benefits are to be expected by the patients included in this biomedical research.

Risks: These are the risks potentially attributable to the performance of locoregional analgesia by the epidural route. These risks appear to be very low according to the currently available data and should be balanced with the high morbidity and mortality attributable to the medical condition under study.

The constraints are null for the patients, and minimal for care teams experienced in anesthesia and intensive care, and specialized in the implementation and management of epidural analgesia.

2.4. Expected impact

The use of thoracic epidural analgesia may reduce the need for tracheal intubation and invasive mechanical ventilation, and may limit the duration of invasive ventilation when it had become necessary. Taken together, these benefits could improve the prognosis of intensive care unit patients with acute pancreatitis.

2.5. References to scientific literature and relevant data used as a reference for research

1. Peery, A. F. *et al.* Burden of gastrointestinal disease in the United States: 2012 update. *Gastroenterology* **143**, 1179–87.e1–3 (2012).
2. Swaroop, V. S., Chari, S. T. & Clain, J. E. Severe acute pancreatitis. *JAMA* **291**, 2865–2868 (2004).
3. Lankisch, P. G., Apte, M. & Banks, P. A. Acute pancreatitis. *Lancet* **386**, 85–96 (2015).
4. Acute Respiratory Distress Syndrome: The Berlin Definition. *JAMA* **307**, (2012).
5. Whitcomb, D. C. Clinical practice. Acute pancreatitis. *N. Engl. J. Med.* **354**, 2142–2150 (2006).
6. Baron, T. H. & Morgan, D. E. Acute necrotizing pancreatitis. *N. Engl. J. Med.* **340**, 1412–1417 (1999).
7. Dombernowsky, T., Kristensen, M. Ø., Rysgaard, S., Gluud, L. L. & Novovic, S. Risk factors for and impact of respiratory failure on mortality in the early phase of acute pancreatitis. *Pancreatology* **16**, 756–760 (2016).

8. Klar, E. *et al.* Impact of microcirculatory flow pattern changes on the development of acute edematous and necrotizing pancreatitis in rabbit pancreas. *Dig. Dis. Sci.* **39**, 2639–2644 (1994).
9. Banks, P. A. *et al.* Classification of acute pancreatitis--2012: revision of the Atlanta classification and definitions by international consensus. *Gut* **62**, 102–111 (2013).
10. Lund, H., Tønnesen, H., Tønnesen, M. H. & Olsen, O. Long-term recurrence and death rates after acute pancreatitis. *Scand. J. Gastroenterol.* **41**, 234–238 (2006).
11. Jung, B. *et al.* [Severe and acute pancreatitis admitted in intensive care: a prospective epidemiological multiple centre study using CClin network database]. *Ann. Fr. Anesth. Reanim.* **30**, 105–112 (2011).
12. Tenner, S., Baillie, J., DeWitt, J., Vege, S. S. & American College of Gastroenterology. American College of Gastroenterology guideline: management of acute pancreatitis. *Am. J. Gastroenterol.* **108**, 1400–15; 1416 (2013).
13. Clemente, A. & Carli, F. The physiological effects of thoracic epidural anesthesia and analgesia on the cardiovascular, respiratory and gastrointestinal systems. *Minerva Anestesiol.* **74**, 549–563 (2008).
14. Bardia, A. *et al.* Combined Epidural-General Anesthesia vs General Anesthesia Alone for Elective Abdominal Aortic Aneurysm Repair. *JAMA Surg.* **151**, 1116–1123 (2016).
15. Von Dossow, V. *et al.* Thoracic epidural anesthesia combined with general anesthesia: the preferred anesthetic technique for thoracic surgery. *Anesth. Analg.* **92**, 848–854 (2001).
16. Rodgers, A. *et al.* Reduction of postoperative mortality and morbidity with epidural or spinal anaesthesia: results from overview of randomised trials. *BMJ* **321**, 1493 (2000).
17. Jabaudon, M. *et al.* Epidural analgesia in the intensive care unit: An observational series of 121 patients. *Anaesth Crit Care Pain Med* **34**, 217–223 (2015).
18. Bernhardt, A., Kortgen, A., Niesel, H. C. & Goertz, A. [Using epidural anesthesia in patients with acute pancreatitis--prospective study of 121 patients]. *Anaesthesiol. Reanim.* **27**, 16–22 (2002).
19. Sadowski, S. M. *et al.* Epidural anesthesia improves pancreatic perfusion and decreases the severity of acute pancreatitis. *World J. Gastroenterol.* **21**, 12448–12456 (2015).
20. Windisch, O., Heidegger, C.-P., Giraud, R., Morel, P. & Bühler, L. Thoracic epidural analgesia: a new approach for the treatment of acute pancreatitis? *Crit. Care* **20**, 116 (2016).
21. Demirag, A. *et al.* Epidural anaesthesia restores pancreatic microcirculation and decreases the severity of acute pancreatitis. *World J. Gastroenterol.* **12**, 915–920 (2006).
22. Bachmann, K. A. *et al.* Effects of thoracic epidural anesthesia on survival and microcirculation in severe acute pancreatitis: a randomized experimental trial. *Crit. Care* **17**, R281 (2013).
23. Ai, K. *et al.* Epidural anesthesia retards intestinal acidosis and reduces portal vein endotoxin concentrations during progressive hypoxia in rabbits. *Anesthesiology* **94**, 263–269 (2001).
24. Freise, H. *et al.* Thoracic epidural analgesia augments ileal mucosal capillary perfusion and improves survival in severe acute pancreatitis in rats. *Anesthesiology* **105**, 354–359 (2006).
25. Freise, H. *et al.* Hepatic effects of thoracic epidural analgesia in experimental severe acute pancreatitis. *Anesthesiology* **111**, 1249–1256 (2009).
26. Enigk, F. *et al.* Thoracic epidural anesthesia decreases endotoxin-induced endothelial injury. *BMC Anesthesiol.* **14**, 23 (2014).
27. Schäper, J. *et al.* Regional sympathetic blockade attenuates activation of intestinal macrophages and reduces gut barrier failure. *Anesthesiology* **118**, 134–142 (2013).
28. Greenberg, J. A. *et al.* Clinical practice guideline: management of acute pancreatitis. *Can. J. Surg.* **59**, 128–140 (2016).
29. Zerem, E. Treatment of severe acute pancreatitis and its complications. *World J. Gastroenterol.* **20**, 13879–13892 (2014).
30. Working Group IAP/APA Acute Pancreatitis Guidelines. IAP/APA evidence-based guidelines for the management of acute pancreatitis. *Pancreatology* **13**, e1–15 (2013).
31. Payen, J. F. *et al.* Assessing pain in critically ill sedated patients by using a behavioral pain scale. *Crit. Care Med.* **29**, 2258–2263 (2001).
32. Aïssaoui, Y., Zeggwagh, A. A., Zekraoui, A., Abidi, K. & Abouqal, R. Validation of a behavioral pain scale in critically ill, sedated, and mechanically ventilated patients. *Anesth. Analg.* **101**, 1470–1476 (2005).
33. WHO's cancer pain ladder for adults. http://www.who.int/cancer/palliative/painladder/en/ (2013).
34. Wu, C. L. *et al.* Efficacy of postoperative patient-controlled and continuous infusion epidural analgesia

versus intravenous patient-controlled analgesia with opioids: a meta-analysis. *Anesthesiology* **103**, 1079–88; quiz 1109–10 (2005).
35. Figueiredo, S. & Benhamou, D. Epidural analgesia in ICU: Useful and effective probably, safe maybe. *Anaesth Crit Care Pain Med* **34**, 185–186 (2015).
36. Harris, P. A. *et al.* Research electronic data capture (REDCap)--a metadata-driven methodology and workflow process for providing translational research informatics support. *J. Biomed. Inform.* **42**, 377–381 (2009).

3. Study Objectives

3.1. Main objective

To determine whether the use of thoracic EA combined to standard care is more effective at increasing ventilator-free days (VFD) at day 30 over standard care alone in critically ill patients with AP.

3.2. Secondary Objectives

- Determine whether EA could affect ICU and hospital length of stay
- Determine whether EA could affect systemic inflammation (plasma biomarker measurements)
- Determine whether EA could affect health care-related costs of the early management of ICU patients with AP.
- Determine whether EA could affect the incidence of major complications of AP in ICU patients at day 30: death, organ failure, sepsis, septic shock, acute respiratory distress syndrome (ARDS), acute respiratory failure, the need for invasive mechanical ventilation, duration of invasive mechanical ventilation, abdominal compartment syndrome, intra- and extra-abdominal infections, peripancreatic necrosis (infected or not), hemodynamic failure defined as the need for vasopressor support, acute kidney injury, need for renal replacement therapy, “walled-off” necrosis requiring drainage (endoscopic, percutaneous, and/or surgical)
- Determine whether EA could affect tolerance to enteral nutrition and/or reduce digestive symptoms such as nausea, vomiting, and ileus
- Determine whether EA could improve pain management in ICU patients with AP
- Determine whether EA could affect the course and prognosis of AP

4. Study Description

4.1. Study design

This study is a prospective, randomized, controlled, multicenter, parallel group interventional trial.

4.2. Research category

This trial is a biomedical research study.

5. Study Population

5.1. Inclusion criteria

- Adult patients admitted to the intensive care unit with severe acute pancreatitis.
- Informed consent to participate, as defined by law (2nd section, first *Code de Santé Publique*).

5.2. Non Inclusion criteria

- Absolute contra-indication for thoracic epidural catheter placement:
  - Prothrombin time <60%, Platelet count < 75G/L, curative anticoagulant therapy with heparin interrupted for less than 8 hours
  - Local infection, active central nervous system infection, history of back surgery associated with a dural space procedure, suspected or confirmed intracranial hypertension,
  - Refractory circulatory shock despite appropriate resuscitation.
- Known allergy to clonidine, ropivacaine or sufentanil, treatment with a monoamine oxidase inhibitor in the previous 15 days
- Age under 18 or under tutelage measures (*curatelle*, *tutelle*)
- Absence of coverage by the French health insurance system (*sécurité social*e)

5.3. Procedure for premature termination of research

The research will be supervised by a steering committee made up of the various research partners: coordinating investigator, methodologist, sponsor representatives, etc.

The steering committee will be composed of sponsor representatives and the research team.

The steering committee may be asked to decide whether to temporarily or permanently halt the trial based on advice from the independent monitoring committee, such as in the event of a significant difference in favor of one of the two study groups (after interim analysis) or because of patient safety issues (serious adverse events).

If he/she wishes, a patient included in the study may, at any time, withdraw consent with no need for justification of his/her decision.

The termination of participation may also be decided for medical reasons on the initiative of the research investigators in charge of the patient.

5.4. Exclusion period and participation in another research

Participation in another randomized trial is not permitted for the duration of the study (31 days). There is no exclusion period.

5.5. Volunteer compensation

There will be no compensation for participating in this research.

5.6. Recruitment modalities

Patient eligibility is sought upon admission to the intensive care unit. After verification of the presence of inclusion criteria and the absence of non inclusion criteria, and after obtaining the consent of the patient himself (or, failing that, that of his trusted person, or a consent according to an emergency inclusion procedure - see below), the patient is included in the study.

The number of participating centers, the average number of patients admitted with AP, and the total planned duration of the study are compatible with the number of inclusions to be made.

6. Study Methodology

6.1. Study outcomes

6.1.1. Primary outcome measure

The primary outcome variable is the number of VFD at day 30, defined as the number of days from day 0 (inclusion) to day 30 after inclusion on which a patient is able to breathe without invasive assistance. A difference in VFD can reflect a difference in mortality, ventilator days, or both.

6.1.2. Secondary outcome measures

- Length of stay in the ICU and in hospital; potential ICU readmission within 30 days from randomization
- Duration of invasive and noninvasive mechanical ventilation at day 30
- Incidence of AP-related complications at day 30: death, organ failure, sepsis, septic shock, ARDS, acute respiratory failure, abdominal compartment syndrome, intra- or extra-abdominal infection, pancreatic necrosis or abscess (infected or not), hemodynamic failure requiring vasopressor therapy, acute kidney injury, the need for renal replacement therapy, infected intra-abdominal abscesses (such as “walled-off” necrosis) requiring drainage (radiological, endoscopic or surgical), intolerance to enteral feeding, digestive symptoms such as nausea, vomiting, and ileus.
- Analgesia scores (VAS, BPS)
- Biological inflammatory and renal response (biomarker analysis)
- Health care costs related to the management of ICU patients with AP within 30 days from randomization

These measures will be collected within 30 days from randomization.

6.2. Description of Research Methodology

This is a prospective, randomized, controlled, multicenter, parallel group interventional trial (biomedical research).

6.3. Description of measures taken to reduce and avoid bias

Web-based electronic randomization will be performed by minimization, considering stratifications by: center, the cause of acute pancreatitis (biliary, alcohol, other), and by the duration since first onset of abdominal pain (less or more than 48 hours).

The study data analysis will be performed by investigators blinded from the randomization group of enrolled patients. Biological assays will also be performed blindly by biologists.

- The risk of confounding bias is reduced by the use of a control group.
- The randomisation procedure is designed to prevent selection bias
- Attrition bias will be controlled by intention-to-treat analysis.

7. Practical implementation of the protocol

7.1. Description of the study procedures performed (description of each visit) / products used in the course of the research

The patient is initially admitted to a participating ICU for the management of AP. If the patient meets all inclusion criteria and has non-inclusion criteria, information on the protocol is provided by the intensive care physician; an information brochure is given to the patient or to its next of kin person.

The patient's written consent to participate in the trial is then sought. In the event that the patient himself or herself is unable to give consent to participate in the study (such as in the presence of delirium or need for sedation), consent will be obtained from the patient's next of kin.

In the latter cases, the consent of the patient himself or herself to continue participation in the study is sought as soon as his or her condition permits. Consent will in all cases be collected and maintained in the patient's medical record.

After consent is obtained, eligible patients will be randomly and electronically assigned to the interventional group (EA combined with standard care) or to the reference group (standard care alone). Because the trial was primarily designed as a pragmatic trial, all patients will be managed by attending physicians as recommended in recent consensual guidelines on the management of severe AP (standard care): early enteral nutrition when possible, resuscitation measures to correct hypovolemia, maintenance of electrolyte balance, correction of acidosis, early diagnosis and supportive treatment of complications [9,12,28–30] Analgesia goals are the same in both groups, with regular evaluation of pain, at least every 4 hours. In conscious and communicating patients, a visual analogue score (VAS) for pain below 40/100 is targeted and a behavioural pain scale (BPS) of 3-4 is targeted in non-communicating patients.[31,32] In both groups, a stepped multimodal approach to pain management will be applied based on routine protocols from each participating centre, and combining opioid, non-opioid +/- adjuvant drugs administered through the oral, enteral and/or intravenous routes, as recommended by the World Health Organization's pain relief ladder.[12,33]

The interventional group consists in applying standard care combined with thoracic EA through an epidural catheter placed in an intervertebral space between the 6th and the 9th thoracic vertebra, and administration of a mixed solution of ropivacaine (2 $mg.mL^{-1}$) and sufentanil (0.5 $\mu g.mL^{-1}$), for at least 72 hours. EA will be provided using a patient-controlled epidural analgesia (PCEA) device, with continuous infusion rate of 5 to 15 $mL.h^{-1}$ and *bolus* of 3 to 10 mL every 10 minutes maximum. If the patient is not able to self-administer EA, nurses are encouraged to

administer *boli* to achieve analgesia goals if necessary. In addition, iterative epidural administrations of clonidine (1 µg.kg^{-1}) may be used by attending physicians to achieve analgesia goals.[34] The drugs used during EA in this trial will be provided in an unblinded manner by the department of Pharmacy at CHU Clermont-Ferrand to all participating centers.

Because of insufficient evidence regarding the optimal duration of EA in ICU patients,[17,18,35] total duration of EA will be chosen by participating physicians for each patient, given that it has been administered for at least 72 hours. Weaning of EA and removal of epidural catheter will be conducted according to recommendations and routine protocols from each participating center.

As a current practice in every participating ICU, close monitoring will be carried out on vital (heart rate, blood pressure, etc.) and hemodynamic parameters.

At days 0, 1, 2, 3, 7, 15 and 30 (or at ICU discharge, whichever occurs first), a medical examination is carried out by the clinician in charge of the patient in order to evaluate the patient and to collect the clinical and biological data for the purpose of the study (such as detailed above), as well as intercurrent events related to the trial if any.

Patient participation in the study ends after day 30 since randomization. If patients are still alive and have been discharged from the hospital at day 30, a telephone follow-up visit is performed by a study physician or a clinical research associate.

7.2. Description of the general logistical organisation of the trial

The clinician in charge of the patient, after verifying the patient's eligibility for participation in the study, obtains the written consent of the patient (or that of his or her support person) after oral and written information on the proposed protocol has been provided.

All patients admitted with AP to participating ICUs will be screened by the ICU team, and inclusion and non inclusion criteria will be sought. This will allow the development of a screening log to collect reasons for non inclusion and to obtain the flowchart of the trial.

Because the trial was primarily designed as a pragmatic trial, all patients will be managed by attending physicians as recommended in recent consensual guidelines on the management of severe AP (standard care): early enteral nutrition when possible, resuscitation measures to correct hypovolemia, maintenance of electrolyte balance, correction of acidosis, early diagnosis and supportive treatment of complications [9,12,28–30] Analgesia goals are the same in both groups, with regular evaluation of pain, at least every 4 hours. In conscious and communicating patients, a visual analogue score (VAS) for pain below 40/100 is targeted and a behavioural pain scale (BPS) of 3-4 is targeted in non-communicating patients.[31,32] In both groups, a stepped multimodal approach to pain management will be applied based on routine protocols from each participating centre, and combining opioid, non-opioid +/- adjuvant drugs administered through the oral, enteral and/or intravenous routes, as recommended by the World Health Organization's pain relief ladder.[12,33]

The interventional group consists in applying standard care combined with thoracic EA through an epidural catheter placed in an intervertebral space between the 6th and the 9th thoracic vertebra, and administration of a mixed solution of ropivacaine (2 mg.mL^{-1}) and sufentanil (0.5 µg.mL^{-1}), for at least 72 hours. EA will be provided using a patient-controlled epidural analgesia (PCEA) device, with continuous infusion rate of 5 to 15 mL.h^{-1} and *bolus* of 3 to 10 mL every 10 minutes maximum. If the patient is not able to self-administer EA, nurses are encouraged to administer *boli* to achieve analgesia goals if necessary. In addition, iterative epidural administrations of clonidine (1 µg.kg^{-1}) may be used by attending physicians to achieve analgesia goals.[34] The drugs used during EA in this trial will be provided in an unblinded manner by the department of Pharmacy at CHU Clermont-Ferrand to all participating centers.

Because of insufficient evidence regarding the optimal duration of EA in ICU patients,[17,18,35] total duration of EA will be chosen by participating physicians for each patient, given that it has been administered for at least 72 hours. Weaning of EA and removal of epidural catheter will be conducted accordingly to recommendations and routine protocols from each participating centre.

Study data are prospectively collected and managed by trained research coordinators and/or investigators from each participating centre, using REDCap electronic data capture tools hosted at CHU Clermont-Ferrand. [36] REDCap (Research Electronic Data Capture) is a secure, web-based application designed to support data capture for research studies, providing: 1) an intuitive interface for validated data entry; 2) audit trails for tracking data manipulation and export procedures; 3) automated export procedures for seamless data downloads to common statistical packages; and 4) procedures for importing data from external sources.

The following data are collected and registered at ICU admission and upon inclusion: baseline demographics and characteristics (age, sex, weight, height, body temperature, delay between the onset of AP and ICU admission/study inclusion, comorbidities and coexisting conditions), baseline severity of illness (modified Marshall scoring system, Simplified Acute Physiologic Score (SAPS) II, Sequential Organ Failure Assessment (SOFA)), usual clinical and biological variables that are measured in critically ill patients, organ failure and treatments. From inclusion to day 30 will be assessed: survival status, main complications of AP (e.g., organ failure, sepsis), the need for therapeutic interventions (such as surgery or endoscopic manoeuvres, MV (either invasive or noninvasive), vasopressor support, continuous renal replacement therapy and/or antibiotic therapy), duration of MV if required, length of stay in the ICU/hospital. These data will be collected at day 0, 1, 2, 3, 7, 17, and 30.

In addition to the data described above, biomarker assays are planned as a future research prospect of the EPIPAN trial.

Biological samples will be collected in each participating center, prior to shipment of all samples, at the end of the patient recruitment period, to the Department of Medical Biochemistry and Molecular Biology at CHU Clermont-Ferrand for blinded measurements. The persons in charge of the biological collection are the coordinating investigator and Dr Roszyk (Laboratory of Medical Biochemistry and Molecular Biology of Pr Sapin, CHU Estaing, CHU Clermont-Ferrand).

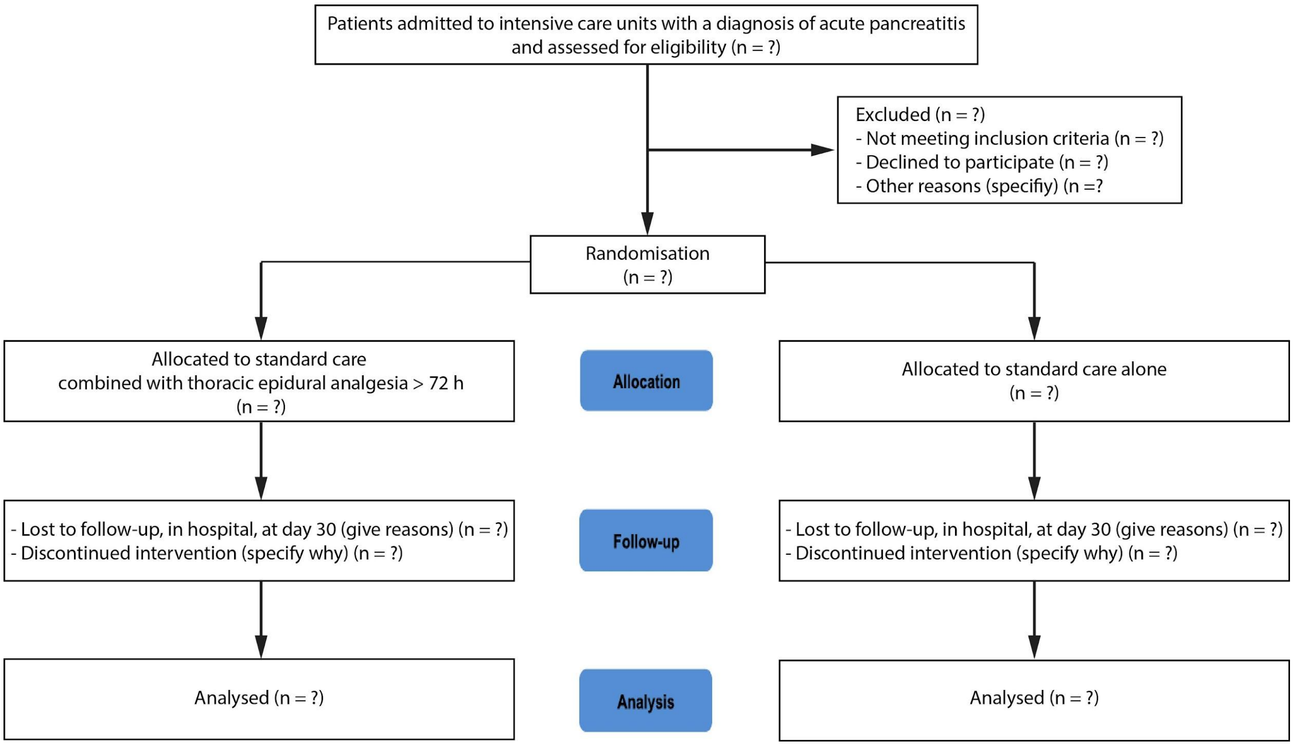

CONSORT diagram of the study illustrating the randomisation and flow of patients in the study.

7.3. Biological sampling and analysis for the study

Creation of a biological collection from blood samples taken from an indwelling arterial catheter (considered as usual care in participating ICUs) during a blood sample procedure as scheduled according to current practice in participating units.

Blood will be sampled at days 0, 2, and 7. Therefore, a total of 3 blood samples of 9 mL each (27 mL total) will be taken during the first week after inclusion.

Measurements will be performed in duplicate at each timepoint for:

- plasma interleukine (IL-)6, a proinflammatory cytokine, using ELISA (R&D Systems, Minneapolis, Minnesota, USA)
- plasma soluble receptor for advanced glycation end-products (sRAGE), a marker of lung alveolar epithelial injury, using ELISA (R&D Systems, Minneapolis, Minnesota, USA)
- plasma neutrophil gelatinase-associated lipocalin (NGAL), a biomarker of acute kidney injury, using the *Triage Meter* (Alere, San Diego, California, USA)

The constitution and conservation of the biological collection after the assays described above have been carried out will allow subsequent biological analyses related to the biological mechanisms to be carried out and to be defined, analyses related to future AP and EA research. The consent from patients for such future biological analyses (as not yet specified) will be sought at the time of inclusion in the EPIPAN study.

7.4. Différence par rapport à la prise en charge de routine.

Par rapport à la prise en charge de routine, la participation à cette étude implique pour le patient :

- De bénéficier d’une analgésie péridurale (uniquement dans un groupe)

- La réalisation de 3 prélèvements sanguins de 9 ml chacun (à J0, J2 et J7) à partir d'un cathéter artériel déjà en place.
- Un contact téléphonique à J30, si le patient n'est plus hospitalisé, afin de s'enquérir de son état de santé.

Dans les centres participant à cet essai, le traitement à l'étude (l'APD) est une technique d'analgésie régulièrement utilisée chez les patients présentant une pancréatite aiguë. Il n'est donc pas à considérer comme un surcoût.

7.5. Expected duration of participation of individuals and description of the trial chronology

Estimated duration of the study: 3 years
Estimated study start date (first patient included): february 2014
Estimated end date (end of follow-up of the last patient enrolled in the study): february 2017
Total duration of patient participation in the study: 31 days

The date of completion of the study will be transmitted to the competent regulatory authority (ANSM) and the Ethics committee (CPP) within 90 days.
In case of premature termination of the study, the information will be transmitted within 15 days to the ANSM and CPP.

8. Intervention (drug / medical device / other) under investigation

8.1. Description of the treatment

All patients will be managed by attending physicians as recommended in recent consensual guidelines on the management of severe AP (standard care): early enteral nutrition when possible, resuscitation measures to correct hypovolemia, maintenance of electrolyte balance, correction of acidosis, early diagnosis and supportive treatment of complications. [9,12,28–30] Analgesia goals are the same in both groups, with regular evaluation of pain, at least every 4 hours. In conscious and communicating patients, a visual analogue score (VAS) for pain below 40/100 is targeted and a behavioural pain scale (BPS) of 3-4 is targeted in non-communicating patients.[31,32] In both groups, a stepped multimodal approach to pain management will be applied based on routine protocols from each participating centre, and combining opioid, non-opioid +/- adjuvant drugs administered through the oral, enteral and/or intravenous routes, as recommended by the World Health Organization's pain relief ladder.[12,33]

The interventional group consists in applying standard care combined with thoracic EA through an epidural catheter placed in an intervertebral space between the 6th and the 9th thoracic vertebra, and administration of a mixed solution of ropivacaine (2 $mg.mL^{-1}$) and sufentanil (0.5 $\mu g.mL^{-1}$), for at least 72 hours. EA will be provided using a patient-controlled epidural analgesia (PCEA) device, with continuous infusion rate of 5 to 15 $mL.h^{-1}$ and *bolus* of 3 to 10 mL every 10 minutes maximum. If the patient is not able to self-administer EA, nurses are encouraged to administer *boli* to achieve analgesia goals if necessary. In addition, iterative epidural administrations of clonidine (1 $\mu g.kg^{-1}$) may be used by attending physicians to achieve analgesia goals.[34] The drugs used during EA in this trial will be provided in an unblinded manner by the department of Pharmacy at CHU Clermont-Ferrand to all participating centers.

Because of insufficient evidence regarding the optimal duration of EA in ICU patients,[17,18,35] total duration of EA will be chosen by participating physicians for each patient, given that it has been administered for at least 72 hours. Weaning of EA and removal of epidural catheter will be conducted according to recommendations and routine protocols from each participating centre.

Main potential side effects of thoracic EA are pruritus (14%), nausea (11%), transient paresis of the lower limbs (1%), and hypotension (2.4%). The need for naloxone, a morphine-antagonist, is exceptional and is usually associated with significant overdose. Only the risk of urine retention remains frequent, but it is routinely and constantly monitored in the ICU setting. In addition, a significant number of ICU patients have a bladder catheter.

Contraindications to the EA technique are rare and mainly include contraindications to the puncture of the epidural space (see non inclusion criteria). Epidural catheter insertion and removal must be performed during isocoagulation, according to the rules of good practice for peri-medullary anaesthesia.

8.2. Dosage, method of administration and duration of treatment

Analgesia goals are the same in both groups, with regular evaluation of pain, at least every 4 hours. In conscious and communicating patients, a visual analogue score (VAS) for pain below 40/100 is targeted and a behavioural pain scale (BPS) of 3-4 is targeted in non-communicating patients.[31,32] In both groups, a stepped multimodal approach to pain management will be applied based on routine protocols from each participating centre, and combining opioid, non-opioid +/- adjuvant drugs administered through the oral, enteral and/or intravenous routes, as recommended by the World Health Organization's pain relief ladder.[12,33]

The interventional group consists in applying standard care combined with thoracic EA through an epidural catheter placed in an intervertebral space between the 6th and the 9th thoracic vertebra after local anesthesia, and administration of a mixed solution of ropivacaine (2 $mg.mL^{-1}$) and sufentanil (0.5 $\mu g.mL^{-1}$), for at least 72 hours. EA will be provided using a patient-controlled epidural analgesia (PCEA) device, with continuous infusion rate of 5 to 15 $mL.h^{-1}$ and *bolus* of 3 to 10 mL every 10 minutes maximum. If the patient is not able to self-administer EA, nurses are encouraged to administer *boli* to achieve analgesia goals if necessary. In addition, iterative epidural administrations of clonidine (1 $\mu g.kg^{-1}$) may be used by attending physicians to achieve analgesia goals.[34] The drugs used during EA in this trial will be provided in an unblinded manner by the department of Pharmacy at CHU Clermont-Ferrand to all participating centers.

Because the trial was primarily designed as a pragmatic trial, all patients will be managed by attending physicians as recommended in recent consensual guidelines on the management of severe AP (standard care): early enteral nutrition when possible, resuscitation measures to correct hypovolemia, maintenance of electrolyte balance, correction of acidosis, early diagnosis and supportive treatment of complications. [9,12,28–30]

8.3. Presentation of the drugs

The Summaries of Product Characteristics (French official *Résumés des Caractéristiques des Produits RCP*) for drugs administered via the epidural route are available on the ANSM website.

SUFENTANIL (morphine derivative)

Morphine derivatives provide good quality, predictable and long-lasting analgesia by the epidural route. Epidurally administered sufentanil does not cause motor or sympathetic block and

does not result in loss of thermal or tactile sensitivity. Only the perception of painful stimuli is diminished.

ROPIVACAINE (local anesthetic)

Ropivacaine provides long-lasting analgesia with a larger sensory block than motor block. Tachyphylaxis for ropivacaine is less a risk than with other short-acting amide radical local anaesthetics (lidocaine, prilocaine and mepivacaine). Ropivacaine has a similar pharmacological profile to bupivacaine, which remains the most widely used local anesthetic, but its toxicity, particularly cardiac, is less. At the same concentration, the motor block is also less intense.

COMBINATION OF LOCAL ANAESTHETICS AND MORPHINE DERIVATIVES

The combination of local anaesthetics and morphine derivatives by the epidural route dhas a synergistic effect that improves the quality of analgesia and allows the dose of each product to be reduced. Numerous publications have shown the effectiveness of such combinations with resting pain scores below 20 mm on a visual analogue scale (VAS) for the majority of patients and better analgesia on mobilization than that obtained with other techniques. The main advantage of this combination is a reduction in side effects related to local anaesthetics (such as motor block, hypotension, tachyphylaxis).

CLONIDINE (adjunct to loco-regional analgesia)

Epidural clonidine acts on alpha-2 adrenergic receptors in the posterior horn of the marrow, and also has its own analgesic effect, probably by acting on muscarinic and nicotinic receptors. The mechanism of action is different from that of morphine derivatives and local anaesthetics, but it potentiates the analgesic action of local anaesthetics and prolongs the sensory block. Its action begins within 15 to 20 minutes, with a peak of action between 60 and 90 minutes after punctual epidural injection. The side effects of epidural clonidine are hypotension and sedation. This sedative effect is sometimes sought in situations where epidural analgesia has been inadequate for some time.

8.4. Issuance and compliance

THE PRESCRIPTION: It is carried out by an authorised professional, specialised in anesthesiology and critical care medicine, and depends on the benefit-risk ratio for the patient and according to the randomization arm.

DISPENSATION: Only the pharmacist is authorized to dispense medication. However, pharmacy interns, fifth-year university hospital students and pharmacy assistants may, in part, dispense under the responsibility of the pharmacist.

- Prescribed medicines are dispensed within a period of time appropriate to their use in a nominative manner from the prescriptions. Medicines are prepared for each patient, at variable intervals (daily, weekly, etc.), if possible on a per-use basis; the dispensing of medicines is recorded and traceability is ensured in accordance with the regulations in a computerized manner.
- Information and advice on the proper use of the medicinal product should be provided to clinical professionals and patients by pharmacy staff under the responsibility of the pharmacist. They shall be subject to traceability.
- The person in charge of transporting drugs from the pharmacy to the clinical areas is

identified. The transport conditions guarantee safe and hygienic transport.

THE ADMINISTRATION: As the treatment is only administered by paramedical or medical resuscitation teams, no control of treatment compliance is foreseen. All participating centers will have the drugs planned for this project (ropivacaine, sufentanil and clonidine) at their disposal.

STOCK MANAGEMENT IN CLINICAL AREAS: The stock management is established according to terms and conditions determined by pharmacy and clinical care units.

THERAPEUTIC MONITORING OF THE PATIENT : Therapeutic monitoring of the patient makes it possible to evaluate the benefit rendered and to identify the possible occurrence of any adverse effects, including minor ones. The latter are recorded in the patient's file, reported to the Regional Pharmacovigilance Center, serious adverse reactions, unexpected adverse reactions and harmful and unintended reactions resulting from misuse.

8.5. Drugs and treatments allowed or not allowed during the trial

No drugs or treatments are prohibited during the EPIPAN trial.

9. Collected data

The clinico-biological data collected during the study by the investigators and clinical research associates are those that are usually collected in the medical record of an ICU patient. Data will be anonymized by the study-specific code assigned to each upon randomization, and include:

- baseline demographics and characteristics (age, sex, weight, height, body temperature, delay between the onset of AP and ICU admission/study inclusion, comorbidities and coexisting conditions)
- baseline severity of illness (modified Marshall scoring system, Simplified Acute Physiologic Score (SAPS) II, Sequential Organ Failure Assessment (SOFA))
- usual clinical and biological variables that are measured in critically ill patients, cause of AP, organ failure and treatments.

From inclusion to day 30 will be assessed: survival status, main complications of AP (e.g., organ failure, sepsis), the need for therapeutic interventions (such as surgery or endoscopic manoeuvres, MV (either invasive or noninvasive), vasopressor support, continuous renal replacement therapy and/or antibiotic therapy), duration of MV if required, length of stay in the ICU/hospital. Biological samples will be collected in each participating centre, prior to shipment of all samples to the Department of Medical Biochemistry and Molecular Biology at CHU Clermont-Ferrand for blinded measurements.

10. Statistical Methods

10.1. Sample size estimation

According to previous studies from the literature,[11,19] we have estimated that a sample size of n = 74 patients per group would provide 80% statistical power to detect an absolute between-group difference of 7 days (with a SD of ± 15) in the primary outcome, i.e. in the number of VFD at day

30 after randomization (expected number of VFD at day 30: 20 ± 15 vs. 13 ± 15 in the intervention and control arms, respectively), for a two-sided type I error of 5%.

Given theoretical concerns related to possible adverse effects of EA in ICU patients, an interim safety analysis will be performed after data for 74 patients are collected, taking into account the inflation of the 1st species error risk (α=0.03, Kim-DeMets, software ©East). The independent Data and Safety Monitoring Board (DSMB) will recommend that the trial be stopped if it is found that the conduct of the trial compromises patient safety (a between-group difference in mortality or VFD at day 30).

10.2. Statistical analysis

A predefined statistical analysis plan will be followed. Statistical analyses will be conducted using Stata software (version 14, StataCorp, College Station, USA). A two-sided p-value of less than 0.05 will be considered to indicate statistical significance.

Concerning the primary outcome, the comparison between interventional and reference groups will be analysed using Student's t-test or Mann-Whitney's test if assumptions of t-test are not met. Normality will be studied by the Shapiro-Wilk test and homoscedasticity using the Fisher-Snedecor test. Results will be expressed as effect-sizes and 95% confidence intervals. Intention to treat (ITT) analysis of data from all randomised patients (except patients who withdraw their consent and those who do not meet the inclusion criteria), including those from the interventional group who do not receive EA for at least 72 hours, will be considered for the primary analysis. Then, the analysis of the primary outcome will be completed by multivariate analysis using a linear mixed model to take into account: (1) fixed effects covariates determined according to univariate results and to clinical relevance (duration of symptoms (either above or below 48 hours from first symptoms, e.g. abdominal pain, to inclusion) and the cause of acute pancreatitis: biliary, alcohol, other) and (2) centre as random-effects (to measure between and within centre variability). The normality of residuals will be studied as described previously. Results will be expressed as regression coefficients and 95% confidence intervals.
Other continuous endpoints (e.g., level of sedation using the Richmond Agitation-Sedation Scale, analgesia scores, doses of drugs, length of stay in ICU/hospital, levels and kinetics of biological markers, duration of MV, and healthcare-related costs at day 30) will be analysed in the same way.

Categorical parameters (death, organ failure, severe sepsis, septic shock, ARDS, the need for MV, acute respiratory failure, abdominal compartment syndrome, intra- or extra-abdominal sepsis, pancreas necrosis (infected or not) as assessed by computed tomography, hemodynamic failure requiring vasopressor support, acute kidney injury, the need for renal replacement therapy, intra-abdominal collection requiring radiological, surgical or endoscopic drainage) will be analysed using Chi-squared or Fisher's exact tests for univariate analysis and generalized linear mixed model (logistic for dichotomous dependent endpoint or Poisson if more appropriate) for multivariate analysis. Type I error will be adjusted using the Hochberg method if appropriate. Results will be expressed as relative risks and 95% confidence intervals. These data will also be analysed as censored data, when appropriate; survival analyses will be performed with the Kaplan-Meier estimator and differences between groups will then be assessed using the log-rank test. The assumption of log-linearity of risk and the proportional hazards will be checked beforehand. Results will be expressed as hazard ratios and 95% confidence intervals. The tolerance of enteral nutrition and/or the incidence of signs of gastrointestinal intolerance (nausea, vomiting, and ileus) will be analysed similarly.

Longitudinal analyses of repeated measures (levels on days 0, 2 and 7 after inclusion of biological markers of systemic inflammation, lung epithelial injury and acute kidney injury will be studied using random-effect models (linear or generalized linear), to take into account patients as random-effect (slope and intercept), nestled in centre random-effect.

According to clinical relevance and to CONSORT recommendations, subgroup analyses depending on the presence or the absence of epidural analgesia will be proposed after the study of subgroup x randomisation group interaction in regression models.

Per-protocol analyses will also be conducted after intention-to-treat analysis is performed. Results from per-protocol analyses will be compared to those from intention-to-treat analyses. A particular focus will be given to safety and patients who are lost to follow-up. A sensitivity analysis will be performed and the nature of missing data will be studied (missing at random or not). According to this study, the most appropriate approach to the imputation of missing data will be proposed (maximum bias (e.g., last observation carried forward vs. baseline observation carried forward) or estimation proposed by Verbeke and Molenberghs for repeated data).

10.3. Data management and analysis

Responsible person;
Bruno PEREIRA, PhD Biostatistics
Délégation Recherche Clinique & Innovation
CHU de Clermont-Ferrand - Villa annexe IFSI
58, Rue Montalembert, 63003 Clermont-Ferrand cedex, France
Mail : bpereira@chu-clermontferrand.fr / Tel : (33) 473 754 964

11. Safety assessment – Management of adverse events

The investigator is responsible for reporting all adverse events.

11.1. Definitions

Adverse event: any untoward medical occurrence in a patient or clinical investigation subject administered a pharmaceutical product and which does not necessarily have to have a causal relationship with the research or with this treatment.

Adverse effect: any untoward response related to the research.

Serious adverse effects are sub-grouped as follows:

- ***Expected*** *serious adverse event*: any event that is described in the most recent version of the Investigator's Brochure, or in the Summary of Product Characteristics for marketed medicinal products, or in the instruction notice when the research concerns a medical device which is subject to CE marking. This definition also applies to an investigational medicinal product when administered for a same population outside the labeled indications.
- ***Unexpected*** *serious adverse event*: any event, the nature, severity or outcome of which is not consistent with the information in the most recent version of the Investigator's Brochure or the Summary of Product Characteristics for a marketed medicinal product or the information notice for a medical device.

Serious adverse event or effect: any undesirable event or effect which results in death, is life-threatening, requires in-patient hospitalization or prolongation of existing hospitalization, results in persistent or significant disability/incapacity, or is a congenital anomaly/birth defect.

The term "life-threatening" refers to an event in which the patient was at risk of death at the time of the event, independently of the consequences of corrective or palliative treatment.

The terms "*disability*" or "*incapacity*" refer to any clinically significant, temporary or persistent disability.

Death, regardless of the cause, including when it corresponds to progression of the disease under treatment, is considered a serious adverse event.

Other events which do not correspond to the above definitions can be considered "*potentially serious*", in particular certain laboratory anomalies. The investigator or sponsor's medical judgement can result in such events being reported in the same manner as "serious" events. It is necessary for study protocols to specify the characteristics of "potentially serious" events that are subject to reporting.

New information: event concerning the conduct of the research or the development of the medicinal product or related product, which is the object of the research, when said new information may jeopardize the safety of the research subjects. Examples include:

- an increase in the rate of occurrence of serious events;
- results of interim analyses, when relevant to the safety of the research subjects (notably a lack of efficacy);
- serious adverse events related to the clinical trial procedures;
- lack of efficacy with a medicinal product used to treat life-threatening disease;
- a major safety finding from animal studies that provides new information on the safety of the product;
- and generally, any new information that could lead to an unfavorable reassessment of the benefit/risk ratio of the research.

Any new information concerning the research (or the product used) which may jeopardize the safety of the research subjects will be subjected to appropriate urgent measures and prompt and timely notification by the Sponsor to the competent authority and the Ethics Committee.

11.2. Serious adverse event reporting

It is the investigator's obligation to report within 24 hours any serious adverse event occurring in any patient enrolled in a study:

- During the active phase of the study,
- In the weeks following cessation of treatment,
- Within the deadlines established for safety monitoring off treatment, before (wash-out or withdrawal phase) or after the active phase,
- After termination of the study, regardless of the time of the event, when no cause other than the research can reasonably be incriminated,
- On the "Serious adverse event report form", indicating the date of onset, the severity, the causal relationship with the treatment (or product), and the follow-up/outcome.

The narrative describing the event should be completed and transmitted to the sponsor as soon as new, relevant information is received. Depending on the nature and seriousness of the event, copies of the patient's anonymized medical record can be attached, as well as laboratory results.

When a serious adverse event persists at the end of the study, the investigator will continue to follow the patient until said event is considered resolved.

In accordance with the implementing decree 2006-477 of 26/04/2006 amending chapter 1 of title II of Book I of the first part of the Public Health Code relating to biomedical research, all suspected unexpected serious adverse effects must be reported by the sponsor to ANSM and to the Ethics Committee at first knowledge and no later than:

- 7 days after occurrence in case of death or a life-threatening event
- 15 days after occurrence for all other unexpected serious adverse events (SAE).

The sponsor will decide upon the significance of the serious adverse events that it reports and the consequences thereof, in particular with respect to the conduct of the research.

The sponsor will also assess the causality of the adverse event with the research by means of a joint analysis with the Regional Pharmacovigilance Center.

The sponsor will maintain a detailed list of all adverse events reported by the investigator(s).

Once per year, or on request, the sponsor will submit an annual safety update report to ANSM and to the Ethics Committee containing all available safety information.

The sponsor will also provide the investigators with any information that may affect the safety of the research subjects.

In this study, only severe adverse events related to EA are potentially expected. Complications of AP will be collected (see full list in Annex 1).

11.3. Independent data monitoring and safety board (DMSB)

A steering committee will be set up to finalize and validate the protocol and the eCRF, and will monitor the progress of the study in order to facilitate the development of the project. It will be able to make decisions concerning the course of the study and if deemed necessary, to propose amendments to the protocol, decide on the opening or closing of centers and discuss the strategy for publishing the results at the end of the trial.

An independent DSMB, composed of three experts (Prs. Hervé Dupont, Thomas Lescot and Philippe Montravers) will monitor the safety of the trial. The DSMB will be responsible for safeguarding the interests of trial participants, assessing the safety and efficacy of the interventions during the trial, and for monitoring the overall conduct of the clinical trial. To contribute to enhancing the integrity of the trial, the DSMB may also formulate recommendations relating to the recruitment/retention of participants, their management, improving adherence to protocol-specified regimens and retention of participants, and the procedures for data management and quality control.

11.4. End of the study / Study interruption

The research will be supervised by a steering committee made up of the various research partners: coordinating investigator, methodologist, sponsor representatives, etc.

The steering committee will be composed of sponsor representatives and the research team.

The steering committee may be asked to decide whether to temporarily or permanently halt the trial based on advice from the independent monitoring committee, such as in the event of a significant difference in favor of one of the two study groups (after interim analysis) or because of patient safety issues (serious adverse events).

If he/she wishes, a patient included in the study may, at any time, withdraw consent with no need for justification of his/her decision.

The termination of participation may also be decided for medical reasons on the initiative of the research investigators in charge of the patient.

11.5. Follow-up of subjects with an adverse event

All patients with an adverse event will be monitored until the event is resolved or stabilized.

12. Right of access to source document and data

12.1. Access to data

The sponsor is responsible for obtaining the agreement of all parties involved in the research in order to guarantee direct access to all study sites, source data, source documents and reports for purposes of the sponsor's quality control and audit.

The investigators will provide access to the documents and individual data that are strictly necessary for purposes of monitoring, quality control and audit of the biomedical research, to the persons authorized to consult said documents pursuant to the legislative and regulatory provisions in force (articles L.1121-3 and R.5121-13 Public Health Code).

12.2. Source data

Source documents, defined as any original document or object which proves the existence or accuracy of data or information recorded during the clinical study, will be stored for a period of 15 years by the investigator or by the hospital in the case of a hospital medical record.

12.3. Data confidentiality

Subject to the provisions relating to the confidentiality of data to which persons in charge of quality control of biomedical research have access (article L.1121-3 Public Health Code), and subject to the provisions relating to the confidentiality of information as concerns in particular the nature of the products being studied, the trials, the persons undergoing the research and the results obtained (article R.5121-13 Public Health Code), persons having direct access shall take all necessary precautions to ensure the confidentiality of the information relating to the products being studied, the trials, the persons undergoing the research and notably their identity, and the results obtained.

These persons, as well as the investigators themselves, are bound by professional secrecy (in accordance with the conditions laid down in articles 226-13 and 226-14 of the penal code).

During the biomedical research or upon its completion, the data collected on the research subjects and transmitted to the sponsor by the investigators (or any other specialized study staff) shall be rendered anonymous.

In no case shall the names or addresses of the persons undergoing the research appear.

Anonymity of the subjects will be guaranteed by the creation of a subject identifying number.

The sponsor will ensure that each research subject has given his written consent allowing access to his personal data, which is strictly necessary for quality control of the research.

12.4. Registration in the national file of biomedical research subjects

Not applicable (no participation of healthy volunteers).

13. Quality control and assurance

13.1. Engagement of the investigators and the sponsor of the study

The investigator undertakes to conduct the study in compliance with public health law 2004-806 of 9 August 2004 relating to biomedical research, the implementing decree 2006-477 of 26/04/2006 amending chapter 1 of title II of book 1 of the first part of the Public Health Code relating to biomedical research, and with the bylaws in force.
The study will also be conducted in compliance with Good Clinical Practices for biomedical research on medicinal products for human use, as laid down in article L.1121-3 Public Health Code and the decree of 24 November 2006.
The investigator also undertakes to comply with the Declaration of Helsinki of the World Medical Assembly (Tokyo 2004, revision).

13.2. Quality assurance

Clinical Research Associates (CRAs) designated by the sponsor will ensure the proper conduct of the study, the collection of data generated in writing, and their documentation, recording and reporting, as per the Standard Operating Procedures in effect at the CHU Clermont-Ferrand and in compliance with Good Clinical Practices and legislative and regulatory provisions in force.

13.3. Quality control

The investigator guarantees the authenticity of the data collected during the study and accepts the legal provisions authorizing the study sponsor to implement quality control.

The coordinating investigator and associated investigators therefore agree to make themselves available during Quality Control visits by the Clinical Research Associate that will be scheduled at regular intervals. The following items will be examined at each visit:

- Informed consent
- Compliance with the study protocol and procedures
- Quality of data recorded in the case report forms: accuracy, missing data, coherence with source documents (medical records, appointment calendars, original copies of laboratory results, etc.)
- Management of any study products

13.4. Case report form

In order to make the collection and analysis of the data collected during the study easier and more reliable, the free and secure web-based application system REDCap (for Research electronic Data Capture), developed at Vanderbilt University (Nashville, Tennessee, USA), is used. The observation logbook will thus be in electronic format (eCRF).

All the information required by the protocol must be recorded in the eCRFs (secure electronic REDCap system) by the investigating physician at each participating centre, and an explanation must be provided for any missing data. Data should be collected as they are obtained, and transcribed into the logbooks in a clear and legible manner.

Data found to be erroneous in the case report forms should be clearly corrected and the new data should be copied, together with the corrected information, accompanied by the electronic signature of the investigator, the date, and possibly a justification by the investigator or the authorised person who made the correction.

The anonymity of the subjects shall be ensured by means of a code number and the initials of the investigator on all documents necessary for the research, including electronic documents, or by erasure by appropriate means of the nominative data on copies of the source documents, intended to to the research literature.

The computerized data on a file will be declared to the CNIL according to the procedure adapted to the case.

14. Ethical considerations

14.1. Ethics Committee and Medicine Agency

The study protocol, patient information notice and consent form will be submitted to the designated *Comité de Protection des Personnes* (CPP).

Notification of a favorable opinion from the Ethics Committee will be transmitted to the sponsor and to ANSM. The sponsor of the study will send an authorization request to ANSM prior to study start. The study sponsor is defined as per law 2004-806 (August 9, 2004).

14.2. Information for patients and written informed consent form

Patients will be fully and fairly informed, in understandable terms, of the objectives and constraints of the study, the possible risks involved, the necessary monitoring and safety measures, their rights to refuse to participate in the study or the possibility of withdrawing at any time. The investigator should also inform the subjects of the opinion given by the PPC.

Oral and written information about the study will be given at the time of admission to resuscitation. The patient's informed consent will be obtained prior to epidural analgesia. In the event that the patient is unable to give free informed consent himself (in the case of confusional syndrome or sedation for example), the consent of his trusted person will be sought. In the absence of the appointment of a trusted person, or if the trusted person is not present or cannot be contacted, or if no close relative or family member of the patient is present or can be reached, an emergency consent procedure will be carried out with the participation of a physician who is independent of the trial and who is not directly responsible for the patient, in order to confirm the patient's eligibility for the study. In cases where the patient himself/herself has not been able to give consent to participate in the study, his/her consent for continued participation in the study will be sought as soon as his/her state of health permits.

All this information is included on an information and consent form given to the patient. The free, informed and written consent of the patient will be obtained by the investigator. These documents are approved by the competent CPP and are to be used for the trial concerned, to the exclusion of all other documents.

Two original copies shall be co-signed by the investigator and the patient. One copy will be given to the patient and the second copy will be kept in the patient's medical file.

14.3. Protocol amendments

Protocol amendments must be qualified as substantial or non-substantial. According to their nature, they will be the object of a new Ethics Committee opinion and/or authorization from the competent authority.

14.4. Management of the patient enrolled in the study

See section 8 (Intervention (drug / medical device / other) under investigation).

15. Data processing and storage of study documents

15.1 Data entry and processing

At each participating center, data will be collected and entered into the electronic web-based case report form (eCRF) by trial or clinical trained personal (clinical research associate), blinded to the allocation group, under the supervision of the trial site investigators.

Data analysis (including the interim analysis after 50% inclusions are performed) will be carried out at the Biostatistical and Data Management Unit, Department of Clinical Research and Innovation (DRCI), CHU Clermont-Ferrand, by Dr Bruno Pereira, PhD in Biostatistics.

15.2 CNIL

This study enters within the scope of “Reference Methodology” in application of the provisions of the law of 6 August 2004 relating to the protection of natural persons with regard to the processing of personal data and amending the law of 6 January 1978 relating to computer processing, data files and civil liberties. This change was approved by decision of 5 January 2006. The CHU Clermont-Ferrand, which sponsored the study, has signed a commitment to comply with this “Reference Methodology” on March, 15, 2007.

15.3 Data retention and archiving

The following documents will be archived under the study name in the Department of Perioperative Medicine (Prof. Jean-Etienne Bazin, Head of the Department of Perioperative Medicine, CHU Clermont-Ferrand, Clermont-Ferrand, France) until the end of the period of practical usefulness (36 months, including inclusion of patients and data analysis).

These documents are:

Protocol and appendices, and any amendments,

Signed, original information notices and consent forms,

Individual data (authenticated copies of raw data),

Follow-up documents

Statistical analyses

Final study report

At the end of the period of practical usefulness, all documents to be archived, such as defined in procedure PG.06.005 “Management of documentation relating to protocols” of Clermont-Ferrand Hospital will be transferred to the central archives and placed under the sponsor’s responsibility for a period of 15 years after study completion, in accordance with institutional practices. These documents cannot be moved or destroyed without the sponsor’s permission. After the 15 years are up, the sponsor will be consulted for destruction. All the data as well as all documents and reports may be subject to audit or inspection.

16. Funding and insurance

16.1 Estimated cost of the study

The total estimated cost includes :

- the cost of plasma biomarker assays, estimated at 15220 euros
  - sRAGE (R&D Systems): 82 effective doses per kit (unit price including VAT: 450 euros), i.e. 11 kits required for 888 doses (148 patients, duplicate doses, 3-step); estimated cost: 4950 euros
  - IL-6 (R&D Systems): 82 effective doses per kit (unit price including VAT: 530 euros), i.e. 11 kits required for 888 doses (148 patients, duplicate doses, 3-step); estimated cost: 5830 euros.
  - NGAL (Alere): 888 effective doses (unit price including VAT per dose: 5 euros; 148 patients, duplicate doses, 3-step); estimated cost: 4440 euros.
- the cost of the constitution, management and conservation of the biological collection, estimated at 15 euros per patient, i.e. 2220 euros in total.
- the cost of repatriating the samples to Clermont-Ferrand (in one go for each centre), estimated at 600 euros.

The overall cost of this study is therefore estimated at 18,000 euros.

There are multiple requests for funding for the study (co-funding):

- from the CHU of Clermont-Ferrand (AOI 2014, Dr Jabaudon)
- from the French Society of Anesthesia and Resuscitation (SFAR 2014 research grant, Dr Jabaudon)
- with the European Society of Intensive Care Medicine (ESICM) (European Critical Research Network Awards 2014, Dr Jabaudon)

In the event that all or part of this co-funding is not obtained, the investigator undertakes to finance by other means all or part of the study budget as presented above (e.g. through the participation of the Department of Perioperative Medicine, CHU Clermont-Ferrand in clinical studies funded by industrial partners and unrelated to the current research project).

16.2 Study insurance

CIn accordance with regulatory provisions, the CHU Clermont-Ferrand, in its capacity as sponsor, has taken out civil liability insurance covering any damages resulting from the research with the Société Hospitalière d'Assurances Mutuelles (SHAM).

It should be noted that non-observance of the legal conditions of the research (absence of Ethics Committee opinion, absence of ANSM authorization, non-consent of subjects, continuation of a suspended or prohibited study) shall render this coverage void.

17. Communication - Rules for publication

The study will be registered prior to first inclusion on www.clinicaltrials.gov. The study protocol will also be published in a peer-reviewed journal.
The sponsor is the owner of the data and no use or transmission to a third party may be made without its prior consent. The first authors of the publications will be the persons who actually participated in the elaboration of the protocol and its progress as well as in the drafting of the results. A writing committee will be set up including members of the steering committee and investigators to define the order of signatories for this study.
Data will only be disclosed after prior joint agreement of the coordinating investigator, the steering committee and the sponsor. The results will be the subject of communications and publications.

18. Feasibility

18.1. Competence of the teams involved

The framework of the research and its conditions are those of daily practice for acute pancreatitis. The investigators in charge of protocol implementation in each of the centers participating in the research have clinical research experience. Measurements for the assessment criteria will be carried out by professionals trained for this purpose in each centre (anesthesiologists and/or intensive care specialists).

18.2 Recruitment feasibility

The 36 month-duration of the study will be consistent with the recruitment goals of the centers participating in the research. Participating ICUs have high volumes of patient admissions for AP. Taking into account patients who cannot be analyzed at the end of the study, an inclusion of at least 30 patients per center is anticipated for the feasibility of this protocol.

19. Annexes

Annex 1: Definitions of AP-related Complications (Secondary Endpoint Measures) (in French)

Annex 2: Participant timeline

ANNEX 1

DEFINITIONS DES COMPLICATIONS DES PANCREATITES AIGUES

(CRITERES DE JUGEMENT SECONDAIRES)

→ **Ventilation non invasive pour détresse respiratoire aiguë** définie par une polypnée > 25/min avec mise en jeu des muscles respiratoires accessoires ou un pH < 7,25 et un Syndrome de Détresse Respiratoire Aigue (Définition de Berlin) définit par une détresse respiratoire de moins d'une semaine avec un rapport PaO2/FiO2 < 300 mmHg avec une PEEP ou cPAP ≥5 cm d'H2O (43)

→ **Ventilation invasive (intubation) pour détresse respiratoire aiguë** définie par une polypnée > 25, mise en jeu des muscles respiratoires accessoires ou un pH < 7,25 et un Syndrome de Détresse Respiratoire Aigue (Définition de Berlin) définit par une détresse respiratoire de moins d'une semaine avec un rapport PaO2/FiO2 < 300 mmHg avec une PEEP ou cPAP ≥5 cm d'H2O (22)

→ **Défaillance hémodynamique nécessitant un traitement par amines : insuffisance** circulatoire aigue, avec trouble de la perfusion tissulaire qui entraîne une anoxie cellulaire avec déviation anaérobie du métabolisme

→ **Décès**

→ **Syndrome du compartiment abdominal** se définit par une augmentation rapide et incontrôlée de la pression intra-abdominale au delà de 20 mmHg associée, avec un lien de causalité, à au moins une défaillance d'organe

→ **Nécrose pancréatique** de tout ou partie de la glande pancréatique et par des **coulées** inflammatoires extra-pancréatique

→ L' **Insuffisance rénale aiguë** (44) est défini comme

1. une augmentation de la créatinine de ≥ 26.5 µmol/l sur une période de 48h
2. ou une augmentation de la créatinine de ≥ 1.5 fois la valeur de base sur une période de 7 jours;
3. ou une diminution de la diurèse de < 0.5 ml/kg/h sur 6h

→ **Sepsis** défini par la présence d'un SIRS en réponse à un processus infectieux confirmé ; une infection suspectée ou prouvée, ou un syndrome clinique pathognomonique d'une infection.

→ **Sepsis sévère** défini par la présence sepsis avec dysfonction d'organe, hypotension artérielle ou hypoperfusion. **Un état de choc septique** est défini par la présence d'un sepsis avec une hypotension ou une hypoperfusion réfractaire malgré un remplissage vasculaire adéquat et la mise en route de catécholamines en perfusion continue après la chirurgie; le signe d'une hypoperfusion systémique peut être une dysfonction d'organe ou la présence d'un taux de lactate sanguin > 4 mmol/dl. Les autres signes incluent une oligurie, une altération des fonctions cognitives.

ANNEX 2

PARTICIPANT TIMELINE

**Patient (Nom)**	**JO**	**J1**	**J2**	**J3**	**J7**	**J15**	**J30**
**Score de gravité**							
Ranson							
Balthazar							
Sofa, APACHE II							
**Hémodynamique**							
amines,							
**Ventilatoire**							
Mode ventilatoire							
Durée de VM >7j							
FiO2 - PEP							
PaO2/FiO2							
**Traitement**							
PCEA-APD							
Pallier I - II - III							
**Complications des PA**							
mortalité							
DMV							
Sepsis grave, choc septique,							
SDRA							
VM (IOT, VNI)							
Sd du cpt abdo,							
infections intra ou extra-abdominales,							
coulées de nécrose							
IRA, recours EER							
Collection infectée							
**Biologique □**							
CRP, IL6							
Ngal							
sRAGE							
EVA							
BPS							
**Tolérance digestive**							
Iléus paralytique							
Nutrition entérale							
Nutrition parentérale							

Final Study Protocol (version 8; July 3, 2018)

EPIDURAL ANALGESIA FOR ACUTE PANCREATITIS IN THE INTENSIVE CARE UNIT: A MULTICENTER RANDOMIZED CONTROLLED TRIAL

Short title: EPIPAN study (*Epidural Analgesia and Pancreatitis*)

Version : 8 *(English)* **Date: July 03, 2018**

Corresponding to substantial amendment #5.

Sponsor Code	EudraCT or AFSSAPS number
**RBHP 2013 JABAUDON**	**2013-004652-37**

Sponsor

C.H.U. de Clermont-Ferrand
58 Rue de Montalembert
63003 Clermont-Ferrand Cedex 1

Principal Investigator

Dr JABAUDON Matthieu
Réanimation Adultes et Unité de Soins Continus
R2D2 – EA 7281, Université d'Auvergne, Faculté de Médecine
CHU Estaing, CHU Clermont-Ferrand
1 place Lucie Aubrac 63003 Clermont-Ferrand cedex 1
Mail : mjabaudon@chu-clermontferrand.fr
Tél : 04 73 75 05 01 / Fax : 04 73 75 05 00

Methodologist

Bruno PEREIRA
Délégation Recherche Clinique & Innovation, CHU Clermont-Ferrand - Villa annexe IFSI
58, Rue Montalembert
63003 Clermont-Ferrand cedex
Mail : bpereira@chu-clermontferrand.fr

Participating centers

CHU Estaing, CHU Clermont-Ferrand,
1 place Lucie Aubrac, 63003 Clermont-Ferrand cedex 1

CHU Gabriel Montpied, CHU Clermont-Ferrand,
58 Rue de Montalembert, 63003 Clermont-Ferrand Cedex 1

Hôpitaux Universitaires de Genève,
4, rue Gabrielle-Perret-Gentil, CH-1211 Genève 14

Hôpital Saint Eloi, CHU de Montpellier,
80 avenue Augustin Fliche, 34295 Montpellier cedex 5

Groupe Hospitalo Universitaire Carémeau - CHU Nîmes,
Place du Pr Debré, 30029 Nimes cedex

Centre Hospitalier Emile Roux, Le Puy en Velay
BP 352, 43012 Le Puy en Velay

Centre Hospitalier de Cannes,
15 avenue des Broussailles – CS 50008, 06414 Cannes Cedex

Cliniques Universitaires Saint-Luc,
Avenue Hippocrate 10, B-1200 Bruxelles

Centre Hospitalier Lyon Sud,
165, Chemin du Grand Revoyet, 69495 Pierre-Bénite Cedex

CHU de Nancy – Brabois,
Rue du Morvan, 54511 Vandoeuvre les Nancy

Hôpital L'Archet 2, CHU de Nice,
151, route St Antoine de Ginestière CS 23079 - 06202 Nice Cedex 3

CH Annecy Genevois,
1 avenue de l'hôpital, 74370 Epagny Metz-Tessy

Hôpital Edouard Herriot, Hospices Civils de Lyon
5 place d'Arsonval, 69003 Lyon

SUMMARY

Context: Severe acute pancreatitis is responsible for a high mortality of about 20%. The use of mechanical ventilation for acute respiratory failure is in itself a criterion of severity, and is associated with a significant increase in morbidity and mortality and in health care-related costs. The management of severe acute pancreatitis mainly includes early enteral nutrition, fluid and electrolyte resuscitation, and adequate management of pain and of complications. Recent evidence suggests that epidural analgesia may reduce postoperative respiratory complications after major thoracoabdominal surgery and facilitate postoperative rehabilitation. Numerous preclinical studies also suggest that epidural analgesia may have an anti-inflammatory effect and a beneficial effect on splanchnic perfusion or respiratory function.
To date, epidural analgesia has not been investigated in intensive care unit patients with acute pancreatitis, with regards to clinical outcome.

Objectives: The objective of our study is to test the effect of epidural analgesia on lung dysfunction in intensive care unit patients with acute pancreatitis, as we hypothesize that it could limit lung failure requiring invasive mechanical ventilation or the duration of invasive mechanical ventilation.

Type of study: Prospective, randomized, controlled, multicenter, parallel group trial.

Number of participating centers: 13 intensive care units among 12 hospitals in France, Belgium, and Switzerland.

Study description: Prospective, randomized, controlled, multicenter, clinical trial in the intensive care unit. After enrolment, patients will be randomized into two groups: a control group in which available guidelines on analgesia will be applied, and an intervention group in which patients receive thoracic epidural analgesia for at least 3 days. Beyond the analgesic strategy, recent consensual guidelines on the management of acute pancreatitis will be applied.

Primary endpoint measure: The number of days free from invasive mechanical ventilation at day 30 after randomization (VFD30), defined as the number of days from randomization to day 30 on which a patient is able to breathe without invasive assistance. A difference in ventilator-free days can reflect a difference in mortality, ventilator days among survivors, or both.

Number of subjects: To demonstrate an increase in VFD30 from 13 to 20 days between the control group and the intervention group, respectively, a total of 74 patients will be needed in each study arm, with a power of 80% and a bilateral alpha risk of 5%.

Inclusion criteria: Adult patients admitted to the intensive care unit with severe acute pancreatitis (defined as acute pancreatitis that is managed in the intensive care unit).

Exclusion Criteria: Absolute contra-indication for thoracic epidural catheter placement (Prothrombin time <60%, Platelet count < 75G/L, curative anticoagulant therapy with heparin interrupted for less than 8 hours, local infection, active central nervous system infection, history of back surgery associated with a dural space procedure, suspected or confirmed intracranial hypertension); Refractory circulatory shock despite appropriate resuscitation; Known allergy to ropivacaine, sufentanil or clonidine; Age under 18 or under tutelage measures (*curatelle*, *tutelle*); Absence of coverage by the French health insurance system (*sécurité sociale*).

Brief Description of the intervention: Thoracic epidural analgesia will be performed using combined administration of ropivacaine (2 mg/ml) and sufentanil (0.5 µg/ml) through

patient-controlled epidural analgesia (PCEA). PCEA parameters will be fixed as follows: continuous administration of 5 to 15 ml/h and bolus of 3 to 10 ml every 10 minutes. Iterative epidural administration of clonidine (1 µg/kg) will be allowed to achieve analgesia goals.
In both groups, conventional analgesia will include enteral and/or parenteral administration analgesics, ranging from step 1 to step 3 drugs according to the WHO analgesics ladder (including acetaminophen, nefopam, tramadol, opioids). The route, dose and frequency of analgesics administrations will be based on current protocols from participating intensive care units.
Beyond the analgesic strategy, recent consensual guidelines on the management of acute pancreatitis will be applied.

Course of the study: Patients will be informed when admitted to the intensive care unit, and enrolled and randomized after consent has been obtained. Web-based randomization will be performed by minimization, considering stratifications by: center, the degree of severity according to the Marshall score (3 strata of increasing severity according to the maximum score obtained for at least one of the functions evaluated (respiratory, renal, hemodynamic)), and by the duration since first onset of abdominal pain (less or more than 48 hours). The intervention (epidural analgesia) will be administered for a minimum duration of 72 hours. Apart from the modalities of multimodal analgesia, the medical and paramedical management will be based on current guidelines and current protocols from participating centers. Patients will be followed-up until day 31 after randomization, with visits at days 1, 2, 3, 4, 5, 6, 7, 15, and 30.

Study duration: 60 months; Duration of patient participation in the study: 31 days.

Table of Content

General Information **8**
Title of the research study 8
Sponsor code 8
Sponsor 8
Study coordination 8
Investigators 8
Associated partners 10
Places where the research is performed is carried out 11
Data processing 12
Ethics committee (Comité de Protection des Personnes) 12
Estimated schedule of the study 12

Study Rationale / Scientific Justification of the Research **13**
Background and rationale 13
Study objectives 14
Summary of known and foreseeable benefits and risks to research subjects 14
Expected impact 14
References to scientific literature and relevant data used as a reference for research 14

Study Objectives **16**
Main objective 16
Secondary Objectives 16

Study Description **17**
Study design 17
Research category 17

Study Population **17**
Inclusion criteria 17
Non Inclusion criteria 17
Procedure for premature termination of research 17
Exclusion period and participation in another research 18
Volunteer compensation 18
Recruitment modalities 18

Study Methodology **18**
Study outcomes 18
Description of Research Methodology 19
Description of measures taken to reduce and avoid bias 19

Practical implementation of the protocol **19**
Description of the study procedures performed (description of each visit) / products used in the course of the research 19

Description of the general logistical organisation of the trial 20
Biological sampling and analysis for the study 22
Différence par rapport à la prise en charge de routine. 23
Expected duration of participation of individuals and description of the trial chronology 23

Intervention (drug / medical device / other) under investigation 23
Description of the treatment 23
Dosage, method of administration and duration of treatment 24
Presentation of the drugs 24
Issuance and compliance 25
Drugs and treatments allowed or not allowed during the trial 26

Collected data 26

Statistical Methods 27
Sample size estimation 27
Statistical analysis 27
Data management and analysis 28

Safety assessment – Management of adverse events 28
Definitions 28
Serious adverse event reporting 29
Independent data monitoring and safety committee (DMSC) 30
End of the study / Study interruption 31
Follow-up of subjects with an adverse event 31

Right of access to source document and data 31
Access to data 31
Source data 31
Data confidentiality 31
Registration in the national file of biomedical research subjects 32

Quality control and assurance 32
Engagement of the investigators and the sponsor of the study 32
Quality assurance 32
Quality control 32
Case report form 33

Ethical considerations 33
Ethics Committee and Medicine Agency 33
Information for patients and written informed consent form 33
Protocol amendments 34
Management of the patient enrolled in the study 34

Data processing and storage of study documents 34
Data entry and processing 34

CNIL 34
Data retention and archiving 35

Funding and insurance 35
Estimated cost of the study 35
Study insurance 36

Communication - Rules for publication 36

Feasibility 36
Competence of the teams involved 36
Recruitment feasibility 36

Annexes 37

1. General Information

1.1. Title of the research study

EPIDURAL ANALGESIA FOR ACUTE PANCREATITIS IN THE INTENSIVE CARE UNIT: A MULTICENTER RANDOMIZED CONTROLLED TRIAL

Short title: *Epidural Analgesia in Acute Pancreatitis (the EPIPAN study)*
ANSM registration number: **2013-004652-37**
Version n°8 - July 2, 2018

1.2. Sponsor code

RBHP 2013 JABAUDON

1.3. Sponsor

CHU Clermont-Ferrand
58 rue Montalembert
63003 Clermont-Ferrand cedex 1
France

Direction Générale Adjointe – Délégation Régionale à la Recherche Clinique
Tél : 04.73.751.195 / Fax : 04.73.754.730

1.4. Study coordination

Dr JABAUDON Matthieu
Department of Perioperative Medicine, CHU Clermont-Ferrand
GReD, Université Clermont Auvergne, CNRS UMR 6293, INSERM U1103
Clermont-Ferrand, France
Tél : 04 73 75 05 01 / Fax : 04 73 75 05 00 / mjabaudon@chu-clermontferrand.fr

Délégation à la Recherche Clinique et à l'Innovation, CHU Clermont-Ferrand
58 rue Montalembert, 63003 Clermont-Ferrand cedex 1
Tél : 04 73 75 11 95 / Fax : 04 73 75 47 30

1.5. Investigators

1.5.1. *Coordinating investigator*

Dr JABAUDON Matthieu
Department of Perioperative Medicine, CHU Clermont-Ferrand
GReD, Université Clermont Auvergne, CNRS UMR 6293, INSERM U1103
CHU Clermont-Ferrand, 1 place Lucie Aubrac
63003 Clermont-Ferrand cedex 1, France
Tél : 04 73 75 05 01 / Fax : 04 73 75 05 00 / Mail : mjabaudon@chu-clermontferrand.fr

1.5.2. *Co-investigators*

Pr CONSTANTIN Jean-Michel
Department of Perioperative Medicine, CHU Clermont-Ferrand
GReD, Université Clermont Auvergne, CNRS UMR 6293, INSERM U1103
CHU Estaing, CHU Clermont-Ferrand, 1 place Lucie Aubrac
63003 Clermont-Ferrand cedex 1, France
Mail : jmconstantin@chu-clermontferrand.fr

Pr BÜHLER Leo
Service de Chirurgie viscérale et transplantation, Unité d'Investigations Chirurgicales
Hôpitaux Universitaires de Genève
4, rue Gabrielle-Perret-Gentil, CH-1211 Genève 14, Suisse
leo.Buhler@hcuge.ch

Pr JABER Samir
Département d'Anesthésie et Réanimation B (SAR B)
Hôpital Saint-Eloi, CHU de Montpellier, France
Mail : s-jaber@chu-montpellier.fr

Pr LEFRANT Jean-Yves
Service Anesthésie-Réanimation
Hôpital Carémeau, CHU Nîmes, France
Email : jean.yves.lefrant@chu-nimes.fr

Dr CLAUD Bernard
Réanimation et Unité de Soins Continus, Département d'Anesthésie Réanimation
Centre Hospitalier Emile Roux, Le Puy en Velay, France
Mail : bernard.claud@ch-lepuy.fr

Dr CHABANNE Russell
Réanimation polyvalente à orientation neurologique
Hôpital Gabriel Montpied, CHU Clermont-Ferrand, France
Mail : rchabanne@chu-clermontferrand.fr

Dr BERTRAND Pierre-Marie
Réanimation médico-chirurgicale
Centre Hospitalier de Cannes, France
Mail : pm.bertrand@gmail.com

Pr LATERRE Pierre-François
Service de Soins Intensifs
Cliniques Universitaires Saint-Luc, Bruxelles, Belgique
Mail : pierre-francois.laterre@uclouvain.be

Dr FRIGGERI Arnaud
Réanimation Médicale et Chirurgicale
Centre Hospitalier Lyon Sud, France

Mail : arnaud.friggeri@chu-lyon.fr

Dr WALLET Florent
Réanimation Médicale et Chirurgicale
Centre Hospitalier Lyon Sud, France
Mail : florent.wallet@gmail.com

Dr GUERCI Philippe
Réanimation Chirurgicale JM Picard
CHU de Nancy – Brabois, France
Mail : phil.guerci@gmail.com

Dr DANIN Pierre-Eric
Réanimation médico-chirurgicale
Hôpital L'Archet 2 - CHU de Nice, France
Mail : danin.pe@chu-nice.fr

Dr ESCUDIER Etienne
Réanimation polyvalente et Unité de Soins Continus
Centre Hospitalier Annecy Genevois, France
Mail : eescudier@ch-annecygenevois.fr

Dr BONNASSIEUX Martin
Réanimation Chirurgicale Déchocage, Pavillon G
Hôpital Edouard Herriot, Hospices Civils de Lyon, France
Mail : martin.bonnassieux@chu-lyon.fr

1.6. Associated partners

Dr Bruno PEREIRA (biostatisticien)
Délégation Régionale à la Recherche Clinique
CHU de Clermont-Ferrand

Dr Laurence ROSZYK
GReD, Université Clermont Auvergne, CNRS UMR 6293, INSERM U1103
Laboratoire de Biochimie Médicale et Biologie Moléculaire, CHU Clermont-Ferrand, France

Pr Vincent SAPIN
GReD, Université Clermont Auvergne, CNRS UMR 6293, INSERM U1103
Laboratoire de Biochimie Médicale et Biologie Moléculaire, CHU Clermont-Ferrand, France

Dr Etienne IMHOFF
CHU de Clermont-Ferrand, France

Dr Stéphanie BULYEZ
CHU de Clermont-Ferrand, France

1.7. Places where the research is performed is carried out

CHU Estaing, CHU Clermont-Ferrand
1 place Lucie Aubrac, 63003 Clermont-Ferrand cedex 1
Pr Jean-Michel CONSTANTIN, Réanimation Adultes et Unité de Soins Continus, Service Anesthésie Réanimation
Tél : 04 73 75 05 01 / Fax : 04 73 75 05 00 / jmconstantin@chu-clermontferrand.fr

Hôpitaux Universitaires de Genève
4, rue Gabrielle-Perret-Gentil, CH-1211 Genève 14
Pr Leo BÜHLER, Service de Chirurgie Viscérale et Transplantation
Tél : +41 22 372 77 03 / Fax : +41 22 372 77 03 / leo.Buhler@hcuge.ch

Hôpital Saint Eloi, CHU de Montpellier
80 avenue Augustin Fliche, 34295 Montpellier cedex 5
Pr Samir JABER, Service Anesthésie Réanimation
Tél : 04 67 33 72 71 / Fax : 04 67 33 74 48 / s-jaber@chu-montpellier.fr

Groupe Hospitalo Universitaire Carémeau - CHU Nîmes
Place du Pr Debré, 30029 Nîmes cedex
Pr Jean-Yves LEFRANT, Service Anesthésie Réanimation
Tél : 04 66 68 30 50 / Fax : 04 66 68 38 51 / jean.yves.lefrant@chu-nimes.fr

Centre Hospitalier Emile Roux, Le Puy en Velay
BP 352, 43012 Le Puy en Velay
Dr CLAUD Bernard, Réanimation et Unité de Soins Continus, Département d'Anesthésie Réanimation
Tél : 04 71 04 32 10 / Fax : 04 71 04 33 70 / bernard.claud@ch-lepuy.fr

CHU Gabriel Montpied, CHU Clermont-Ferrand
58 Rue de Montalembert, 63003 Clermont-Ferrand Cedex 1
Dr CHABANNE Russell, Service d'Anesthésie Réanimation
Tél : 04 73 75 16 48 / Fax : 04 74 75 13 31 / rchabanne@chu-clermontferrand.fr

Centre Hospitalier de Cannes
15 avenue des Broussailles – CS 50008, 06414 Cannes Cedex
Dr BERTRAND Pierre-Marie, Service de Réanimation Médico-Chirurgicale
Tél : 04 93 69 70 25 / Fax : 04 93 69 75 77 / pm.bertrand@gmail.com

Cliniques Universitaires Saint-Luc, UCL Bruxelles,
Avenue Hippocrate 10, B-1200 BRUXELLES
Pr LATERRE Pierre-François, Service de Soins Intensifs
Tel : 32(0)2 764 11 11 / Fax : 32 (0)2 764 37 03 / pierre-francois.laterre@uclouvain.be

Centre Hospitalier Lyon Sud
165, Chemin du Grand Revoyet, 69495 Pierre-Bénite cedex
Dr FRIGGERI Arnaud, Dr WALLET Florent, Pr PIRIOU Vincent
Réanimation Médicale et Chirurgicale
Tel : 07 78 86 19 88 / Fax : 04 78 86 59 33 / arnaud.friggeri@chu-lyon.fr

CHU de Nancy – Brabois
Rue du Morvan, 54511 Vandoeuvre les Nancy
Dr GUERCI Philippe, Réanimation Chirurgicale JM Picard
Tel : 03 83 15 41 66 / phil.guerci@gmail.com

Hôpital L'Archet 2, CHU de Nice
151, route St Antoine de Ginestière CS 23079 - 06202 Nice Cedex 3
Dr DANIN Pierre-Eric, Service de Réanimation Médico-Chirurgicale
Tel: 04 92 03 64 20/ Fax : 04 92 03 64 19 / danin.pe@chu-nice.fr

CH Annecy Genevois, 1 avenue de l'hôpital, 74370 Epagny Metz-Tessy
Dr ESCUDIER Etienne, Réanimation polyvalente et Unité de Soins Continus
Tel: 04 50 63 69 91 / Fax : 04 50 63 65 60 / eescudier@ch-annecygenevois.fr

Hôpital Edouard Herriot, Hospices Civils de Lyon, 5 place d'Arsonval, 69003 Lyon
Dr **BONNASSIEUX Martin,** Réanimation Chirurgicale Déchocage
Pavillon G**,** Hôpital Edouard Herriot, Hospices Civils de Lyon
Tel : 04 72 11 77 40 / martin.bonnassieux@chu-lyon.fr

1.8. Data processing

Department of Perioperative Medicine, CHU Clermont-Ferrand
GReD, Université Clermont Auvergne, CNRS UMR 6293, INSERM U1103

Délégation Régionale à la Recherche Clinique, CHU Clermont-Ferrand

1.9. Ethics committee (*Comité de Protection des Personnes*)

Comité de Protection des Personnes (CPP) Sud Est VI

1.10. Estimated schedule of the study

- Submission to the sponsor local research committee (COMAP/COMVAL, DRCI, CHU Clermont-Ferrand): august/september 2013
- Submission to the Ethics committee (CPP) : october 2013
- CPP Approval: february 2014
- ANSM Approval: february 2014
- Start of study : june 2014
- Enrolment period: june 2014 - june 2019
- Estimated end of study: july 2019
- End of study report : december 2019

2. Study Rationale / Scientific Justification of the Research

2.1. Background and rationale

Acute pancreatitis (AP) is one of the most frequent gastrointestinal diseases, whose incidence in the US reaches 35 per 100,000 population annually. In 2009, AP was responsible for 275,000 hospital admissions in the USA, with a total cost of over US $2,5 billion.[1,2] AP develops when intracellular protective mechanisms to prevent trypsinogen activation or reduce trypsin activity are overwhelmed[3]. The initiating event may be any insult to the acinar cell that impairs the secretion of zymogen granules, such as alcohol abuse or gallstone migration into the common bile duct. Once the process of cellular injury is initiated, cellular membrane trafficking becomes chaotic, leading to the release of proinflammatory mediators (tumour necrosis factor (TNF)-α, interleukin (IL)-6, and IL-8). These mediators participate to an increase in pancreatic vascular permeability that subsequently favours hemorrhage, oedema and eventually pancreatic necrosis. As these mediators are excreted into the circulation, systemic complications can arise, such as bacteraemia due to gut flora translocation, acute respiratory distress syndrome (ARDS)[4], pleural effusions, gastrointestinal hemorrhage and renal failure.[3,5–8]

The revised Atlanta classification addresses the clinical course and severity of the disease.[9] AP may be divided into two forms, interstitial oedematous pancreatitis, during the first week, and necrotising pancreatitis during a later phase (after 7 days). In approximately 80% of patients, the severity of AP is rather mild and resolves without serious morbidity. However, in up to 20% of patients, AP presents in a more severe form requiring admission to the intensive care unit (ICU) due to persistent organ failure.[9,10] Mortality rate can reach 20-40% in severe AP because of multiorgan failure (MOF) and pancreatic necrosis.[1,11]

The amplifying effects of inflammatory and oxidative impairment often lead to severe AP-induced complications, which are often regarded as hallmarks of severe AP and herald poor outcome. In a recent French observational study of ICU patients with severe AP, 58% of patients developed acute respiratory failure requiring intubation and invasive mechanical ventilation (MV) (mean duration 15 days, standard deviation (SD) 17 days), and such patients had higher mortality rates than those who were not intubated (34% vs 1.4%).[11] Since respiratory failure is the main cause of death in patients with severe AP, more work is needed for us to prevent and treat AP-associated respiratory failure. Despite recent substantial improvements in the multidisciplinary management of AP (e.g., with regards to fluid therapy, intensive care management, prevention of infectious complications, nutritional support, biliary tract management or necrotising pancreatitis management), the prognosis of severe AP remains poor in patients who develop acute respiratory failure requiring intubation and invasive respiratory support.[3,9,12] Of notes, available therapeutic approaches do not have a direct action on the pancreas itself but aim to attenuate the process of MOF present in the severe form of AP, and no causal treatment has been developed yet.

Epidural analgesia (EA) is one of the most widely and versatile utilized neural deafferentation techniques. It is used for analgesia during the perioperative period, but also for obstetrics labour and trauma as well as in the treatment of acute, chronic and cancer-related pain.[13,14] Its objective is not only to block noxious afferent stimuli, but also to induce bilateral selective thoracic sympathetic blockade. In addition to analgesia itself, the modulatory effects of thoracic EA could improve organ perfusion with reduced complications in the perioperative period, thus possibly decreasing postoperative complications, shortening hospital stay and improving survival.[14–16]

EA has not yet been extensively assessed in the ICU setting in general, and in critically ill patients with severe AP in particular. Several studies suggest that thoracic EA might be a safe procedure in centers comprising anaesthesiologists with expertise in EA, and thoracic EA has already been used for years to treat pain during AP in critically ill patients in some centers.[17–19] In

addition, recent animal studies suggest that thoracic EA may decrease the severity of AP, with reduced respiratory, thromboembolic and abdominal complications.[20–22] EA further decreased the severity of metabolic acidosis and tissue injury in animals, thus preventing the progression from oedematous to necrotising AP.[23] EA may also restore pancreatic hypoperfusion induced by AP through blood flow redistribution from splanchnic to non-perfused pancreatic regions,[24,25] and a recent clinical study suggests that EA could increase pancreatic arterial perfusion and improve clinical outcome in patients with AP.[19] Findings from other experimental studies also support beneficial effects of EA in severe AP, such as increased gut barrier function and renal perfusion, decreased liver damage and inflammatory response, and reduced mortality.[22,24,26,27]

Despite such promising findings from preclinical studies, the effects of thoracic EA on major clinical outcomes have never been specifically assessed and its benefit in critically ill patients with AP remains uncertain.

2.2. Study objectives

Primary objective: To determine whether the use of thoracic EA combined to standard care is more effective at increasing ventilator-free days (VFD) at day 30 over standard care alone in critically ill patients with AP. The goal of the EPIPAN trial is therefore to test the impact of thoracic EA on respiratory failure, with the hypothesis that EA could influence survival and/or the need for invasive MV and/or its duration when invasive MV is required.
Secondary objectives: To determine whether in comparison to standard care alone, application of thoracic EA combined with standard care could improve survival, decrease major complications of AP (including sepsis, organ failure), AP-related costs, the need for medical, surgical and radiological interventions, and impact biological markers of systemic inflammation, lung injury and renal failure.

2.3. Summary of known and foreseeable benefits and risks to research subjects

Benefits: No direct benefits are to be expected by the patients included in this biomedical research.
Risks: These are the risks potentially attributable to the performance of locoregional analgesia by the epidural route. These risks appear to be very low according to the currently available data and should be balanced with the high morbidity and mortality attributable to the medical condition under study.
The constraints are null for the patients, and minimal for care teams experienced in anesthesia and intensive care, and specialized in the implementation and management of epidural analgesia.

2.4. Expected impact

The use of thoracic epidural analgesia may reduce the need for tracheal intubation and invasive mechanical ventilation, and may limit the duration of invasive ventilation when it had become necessary. Taken together, these benefits could improve the prognosis of intensive care unit patients with acute pancreatitis.

2.5. References to scientific literature and relevant data used as a reference for research

1. Peery, A. F. *et al.* Burden of gastrointestinal disease in the United States: 2012 update. *Gastroenterology* **143**, 1179–87.e1–3 (2012).
2. Swaroop, V. S., Chari, S. T. & Clain, J. E. Severe acute pancreatitis. *JAMA* **291**, 2865–2868 (2004).

3. Lankisch, P. G., Apte, M. & Banks, P. A. Acute pancreatitis. *Lancet* **386**, 85–96 (2015).
4. Acute Respiratory Distress Syndrome: The Berlin Definition. *JAMA* **307**, (2012).
5. Whitcomb, D. C. Clinical practice. Acute pancreatitis. *N. Engl. J. Med.* **354**, 2142–2150 (2006).
6. Baron, T. H. & Morgan, D. E. Acute necrotizing pancreatitis. *N. Engl. J. Med.* **340**, 1412–1417 (1999).
7. Dombernowsky, T., Kristensen, M. Ø., Rysgaard, S., Gluud, L. L. & Novovic, S. Risk factors for and impact of respiratory failure on mortality in the early phase of acute pancreatitis. *Pancreatology* **16**, 756–760 (2016).
8. Klar, E. *et al.* Impact of microcirculatory flow pattern changes on the development of acute edematous and necrotizing pancreatitis in rabbit pancreas. *Dig. Dis. Sci.* **39**, 2639–2644 (1994).
9. Banks, P. A. *et al.* Classification of acute pancreatitis--2012: revision of the Atlanta classification and definitions by international consensus. *Gut* **62**, 102–111 (2013).
10. Lund, H., Tønnesen, H., Tønnesen, M. H. & Olsen, O. Long-term recurrence and death rates after acute pancreatitis. *Scand. J. Gastroenterol.* **41**, 234–238 (2006).
11. Jung, B. *et al.* [Severe and acute pancreatitis admitted in intensive care: a prospective epidemiological multiple centre study using CClin network database]. *Ann. Fr. Anesth. Reanim.* **30**, 105–112 (2011).
12. Tenner, S., Baillie, J., DeWitt, J., Vege, S. S. & American College of Gastroenterology. American College of Gastroenterology guideline: management of acute pancreatitis. *Am. J. Gastroenterol.* **108**, 1400–15; 1416 (2013).
13. Clemente, A. & Carli, F. The physiological effects of thoracic epidural anesthesia and analgesia on the cardiovascular, respiratory and gastrointestinal systems. *Minerva Anestesiol.* **74**, 549–563 (2008).
14. Bardia, A. *et al.* Combined Epidural-General Anesthesia vs General Anesthesia Alone for Elective Abdominal Aortic Aneurysm Repair. *JAMA Surg.* **151**, 1116–1123 (2016).
15. Von Dossow, V. *et al.* Thoracic epidural anesthesia combined with general anesthesia: the preferred anesthetic technique for thoracic surgery. *Anesth. Analg.* **92**, 848–854 (2001).
16. Rodgers, A. *et al.* Reduction of postoperative mortality and morbidity with epidural or spinal anaesthesia: results from overview of randomised trials. *BMJ* **321**, 1493 (2000).
17. Jabaudon, M. *et al.* Epidural analgesia in the intensive care unit: An observational series of 121 patients. *Anaesth Crit Care Pain Med* **34**, 217–223 (2015).
18. Bernhardt, A., Kortgen, A., Niesel, H. C. & Goertz, A. [Using epidural anesthesia in patients with acute pancreatitis--prospective study of 121 patients]. *Anaesthesiol. Reanim.* **27**, 16–22 (2002).
19. Sadowski, S. M. *et al.* Epidural anesthesia improves pancreatic perfusion and decreases the severity of acute pancreatitis. *World J. Gastroenterol.* **21**, 12448–12456 (2015).
20. Windisch, O., Heidegger, C.-P., Giraud, R., Morel, P. & Bühler, L. Thoracic epidural analgesia: a new approach for the treatment of acute pancreatitis? *Crit. Care* **20**, 116 (2016).
21. Demirag, A. *et al.* Epidural anaesthesia restores pancreatic microcirculation and decreases the severity of acute pancreatitis. *World J. Gastroenterol.* **12**, 915–920 (2006).
22. Bachmann, K. A. *et al.* Effects of thoracic epidural anesthesia on survival and microcirculation in severe acute pancreatitis: a randomized experimental trial. *Crit. Care* **17**, R281 (2013).
23. Ai, K. *et al.* Epidural anesthesia retards intestinal acidosis and reduces portal vein endotoxin concentrations during progressive hypoxia in rabbits. *Anesthesiology* **94**, 263–269 (2001).
24. Freise, H. *et al.* Thoracic epidural analgesia augments ileal mucosal capillary perfusion and improves survival in severe acute pancreatitis in rats. *Anesthesiology* **105**, 354–359 (2006).
25. Freise, H. *et al.* Hepatic effects of thoracic epidural analgesia in experimental severe acute pancreatitis. *Anesthesiology* **111**, 1249–1256 (2009).
26. Enigk, F. *et al.* Thoracic epidural anesthesia decreases endotoxin-induced endothelial injury. *BMC Anesthesiol.* **14**, 23 (2014).
27. Schäper, J. *et al.* Regional sympathetic blockade attenuates activation of intestinal macrophages and reduces gut barrier failure. *Anesthesiology* **118**, 134–142 (2013).
28. Greenberg, J. A. *et al.* Clinical practice guideline: management of acute pancreatitis. *Can. J. Surg.* **59**, 128–140 (2016).
29. Zerem, E. Treatment of severe acute pancreatitis and its complications. *World J. Gastroenterol.* **20**, 13879–13892 (2014).
30. Working Group IAP/APA Acute Pancreatitis Guidelines. IAP/APA evidence-based guidelines for the management of acute pancreatitis. *Pancreatology* **13**, e1–15 (2013).

31. Payen, J. F. *et al.* Assessing pain in critically ill sedated patients by using a behavioral pain scale. *Crit. Care Med.* **29**, 2258–2263 (2001).
32. Aïssaoui, Y., Zeggwagh, A. A., Zekraoui, A., Abidi, K. & Abouqal, R. Validation of a behavioral pain scale in critically ill, sedated, and mechanically ventilated patients. *Anesth. Analg.* **101**, 1470–1476 (2005).
33. WHO's cancer pain ladder for adults. http://www.who.int/cancer/palliative/painladder/en/ (2013).
34. Wu, C. L. *et al.* Efficacy of postoperative patient-controlled and continuous infusion epidural analgesia versus intravenous patient-controlled analgesia with opioids: a meta-analysis. *Anesthesiology* **103**, 1079–88; quiz 1109–10 (2005).
35. Figueiredo, S. & Benhamou, D. Epidural analgesia in ICU: Useful and effective probably, safe maybe. *Anaesth Crit Care Pain Med* **34**, 185–186 (2015).
36. Harris, P. A. *et al.* Research electronic data capture (REDCap)--a metadata-driven methodology and workflow process for providing translational research informatics support. *J. Biomed. Inform.* **42**, 377–381 (2009).

3. Study Objectives

3.1. Main objective

To determine whether the use of thoracic EA combined to standard care is more effective at increasing ventilator-free days (VFD) at day 30 over standard care alone in critically ill patients with AP. The goal of the EPIPAN trial is therefore to test the impact of thoracic EA on respiratory failure, with the hypothesis that EA could influence survival and/or the need for invasive MV and/or its duration when invasive MV is required.

3.2. Secondary Objectives

- Determine whether EA could affect ICU and hospital length of stay
- Determine whether EA could affect systemic inflammation (plasma biomarker measurements)
- Determine whether EA could affect health care-related costs of the early management of ICU patients with AP.
- Determine whether EA could affect the incidence of major complications of AP in ICU patients at day 30: death, organ failure, sepsis, septic shock, acute respiratory distress syndrome (ARDS), acute respiratory failure, the need for invasive mechanical ventilation, duration of invasive mechanical ventilation, abdominal compartment syndrome, intra- and extra-abdominal infections, peripancreatic necrosis (infected or not), hemodynamic failure defined as the need for vasopressor support, acute kidney injury, need for renal replacement therapy, "walled-off" necrosis requiring drainage (endoscopic, percutaneous, and/or surgical)
- Determine whether EA could affect tolerance to enteral nutrition and/or reduce digestive symptoms such as nausea, vomiting, and ileus
- Determine whether EA could improve pain management in ICU patients with AP and reduce the need for analgesics, including opioids
- Determine whether EA could affect the course and prognosis of AP

4. Study Description

4.1. Study design

This study is a prospective, randomized, controlled, multicenter, parallel group interventional trial.

4.2. Research category

This trial is a biomedical research study.

5. Study Population

5.1. Inclusion criteria

- Adult patients admitted to the intensive care unit with severe acute pancreatitis (defined as acute pancreatitis that is managed in the intensive care unit).
- Informed consent to participate, as defined by law (2nd section, first *Code de Santé Publique*).

5.2. Non Inclusion criteria

- Absolute contra-indication for thoracic epidural catheter placement:
  - Prothrombin time <60%, Platelet count < 75G/L, curative anticoagulant therapy with heparin interrupted for less than 8 hours
  - Local infection, active central nervous system infection, history of back surgery associated with a dural space procedure, suspected or confirmed intracranial hypertension,
  - Refractory circulatory shock despite appropriate resuscitation.
- Known allergy to clonidine, ropivacaine or sufentanil, treatment with a monoamine oxidase inhibitor in the previous 15 days
- Age under 18 or under tutelage measures (*curatelle*, *tutelle*)
- Absence of coverage by the French health insurance system (*sécurité sociale*)

5.3. Procedure for premature termination of research

The research will be supervised by a steering committee made up of the various research partners: coordinating investigator, methodologist, sponsor representatives, etc.

The steering committee will be composed of sponsor representatives and the research team.

The steering committee may be asked to decide whether to temporarily or permanently halt the trial based on advice from the independent monitoring committee, such as in the event of a significant difference in favor of one of the two study groups (after interim analysis) or because of patient safety issues (serious adverse events).

If he/she wishes, a patient included in the study may, at any time, withdraw consent with no need for justification of his/her decision.

The termination of participation may also be decided for medical reasons on the initiative of the research investigators in charge of the patient.

5.4. Exclusion period and participation in another research

Participation in another randomized trial is not permitted for the duration of the study (31 days). There is no exclusion period.

5.5. Volunteer compensation

There will be no compensation for participating in this research.

5.6. Recruitment modalities

Patient eligibility is sought upon admission to the intensive care unit. After verification of the presence of inclusion criteria and the absence of non inclusion criteria, and after obtaining the consent of the patient himself (or, failing that, that of his trusted person, or a consent according to an emergency inclusion procedure - see below), the patient is included in the study.

The number of participating centers, the average number of patients admitted with AP, and the total planned duration of the study are compatible with the number of inclusions to be made.

6. Study Methodology

6.1. Study outcomes

6.1.1. Primary outcome measure

The primary outcome variable is the number of VFD at day 30, defined as the number of days from day 0 (inclusion) to day 30 after inclusion on which a patient is able to breathe without invasive assistance. A difference in VFD can reflect a difference in mortality, ventilator days, or both.

6.1.2. Secondary outcome measures

- Length of stay in the ICU and in hospital; potential ICU readmission within 30 days from randomization
- Duration of invasive and noninvasive mechanical ventilation at day 30
- Incidence of AP-related complications at day 30: death, organ failure, sepsis, septic shock, ARDS, acute respiratory failure, abdominal compartment syndrome, intra- or extra-abdominal infection, pancreatic necrosis or abscess (infected or not), hemodynamic failure requiring vasopressor therapy, acute kidney injury, the need for renal replacement therapy, infected intra-abdominal abscesses (such as "walled-off" necrosis) requiring drainage (radiological, endoscopic or surgical), intolerance to enteral feeding, digestive symptoms such as nausea, vomiting, and ileus.
- Analgesia scores (VAS, BPS), need for sedation (drugs, doses, level of sedation using the Richmond Agitation-Sedation Scale) and opioids for analgesia (drugs, doses within 7 days from randomization)
- Biological inflammatory and renal response (biomarker analysis)
- Health care costs related to the management of ICU patients with AP within 30 days from randomization

These measures will be collected within 30 days from randomization.

6.2. Description of Research Methodology

This is a prospective, randomized, controlled, multicenter, parallel group interventional trial (biomedical research).

6.3. Description of measures taken to reduce and avoid bias

Web-based electronic randomization will be performed by minimization, considering stratifications by: center, the degree of severity according to the Marshall score (3 strata of increasing severity according to the maximum score obtained for at least one of the functions evaluated (respiratory, renal, hemodynamic)), and by the duration since first onset of abdominal pain (less or more than 48 hours).

The study data analysis will be performed by investigators blinded from the randomization group of enrolled patients. Biological assays will also be performed blindly by biologists.

- The risk of confounding bias is reduced by the use of a control group.
- The randomisation procedure is designed to prevent selection bias
- Attrition bias will be controlled by intention-to-treat analysis.

7. Practical implementation of the protocol

7.1. Description of the study procedures performed (description of each visit) / products used in the course of the research

The patient is initially admitted to a participating ICU for the management of AP. If the patient meets all inclusion criteria and has non-inclusion criteria, information on the protocol is provided by the intensive care physician; an information brochure is given to the patient or to its next of kin person.

The patient's written consent to participate in the trial is then sought. In the event that the patient himself or herself is unable to give consent to participate in the study (such as in the presence of delirium or need for sedation), consent will be obtained from the patient's next of kin.

In the latter cases, the consent of the patient himself or herself to continue participation in the study is sought as soon as his or her condition permits. Consent will in all cases be collected and maintained in the patient's medical record.

After consent is obtained, eligible patients will be randomly assigned (using the Tenalea web application v4.0.5090.3953, Alex Data management) to the interventional group (EA combined with standard care) or to the reference group (standard care alone). Because the trial was primarily designed as a pragmatic trial, all patients will be managed by attending physicians as recommended in recent consensual guidelines on the management of severe AP (standard care): early enteral nutrition when possible, resuscitation measures to correct hypovolemia, maintenance of electrolyte balance, correction of acidosis, early diagnosis and supportive treatment of complications [9,12,28–30]

Analgesia goals are the same in both groups, with regular evaluation of pain, at least every 4 hours. In conscious and communicating patients, a visual analogue score (VAS) for pain below 40/100 is targeted and a behavioural pain scale (BPS) of 3-4 is targeted in non-communicating patients.[31,32] In both groups, a stepped multimodal approach to pain management will be applied based on routine protocols from each participating centre, and combining opioid, non-opioid +/- adjuvant drugs administered through the oral, enteral and/or intravenous routes, as recommended by the World Health Organization's pain relief ladder.[12,33]

The interventional group consists in applying standard care combined with thoracic EA through an epidural catheter placed in an intervertebral space between the 6th and the 9th thoracic vertebra, and administration of a mixed solution of ropivacaine (2 $mg.mL^{-1}$) and sufentanil (0.5 $\mu g.mL^{-1}$), for at least 72 hours. EA will be provided using a patient-controlled epidural analgesia (PCEA) device, with continuous infusion rate of 5 to 15 $mL.h^{-1}$ and *bolus* of 3 to 10 mL every 10 minutes maximum. If the patient is not able to self-administer EA, nurses are encouraged to administer *boli* to achieve analgesia goals if necessary. In addition, iterative epidural administrations of clonidine (1 $\mu g.kg^{-1}$) may be used by attending physicians to achieve analgesia goals.[34] The drugs used during EA in this trial will be provided in an unblinded manner by the department of Pharmacy at CHU Clermont-Ferrand to all participating centers.

Because of insufficient evidence regarding the optimal duration of EA in ICU patients,[17,18,35] total duration of EA will be chosen by participating physicians for each patient, given that it has been administered for at least 72 hours. Weaning of EA and removal of epidural catheter will be conducted according to recommendations and routine protocols from each participating center.

As a current practice in every participating ICU, close monitoring will be carried out on vital (heart rate, blood pressure, etc.) and hemodynamic parameters.

At days 0, 1, 2, 3, 4, 5, 6, 7, 15 and 30 (or at ICU discharge, whichever occurs first), a medical examination is carried out by the clinician in charge of the patient in order to evaluate the patient and to collect the clinical and biological data for the purpose of the study (such as detailed above), as well as intercurrent events related to the trial if any.

Patient participation in the study ends after day 30 since randomization. If patients are still alive and have been discharged from the hospital at day 30, a telephone follow-up visit is performed by a study physician or a clinical research associate.

7.2. Description of the general logistical organisation of the trial

The clinician in charge of the patient, after verifying the patient's eligibility for participation in the study, obtains the written consent of the patient (or that of his or her support person) after oral and written information on the proposed protocol has been provided.

All patients admitted with AP to participating ICUs will be screened by the ICU team, and inclusion and non inclusion criteria will be sought. This will allow the development of a screening log to collect reasons for non inclusion and to obtain the flowchart of the trial.

Because the trial was primarily designed as a pragmatic trial, all patients will be managed by attending physicians as recommended in recent consensual guidelines on the management of severe AP (standard care): early enteral nutrition when possible, resuscitation measures to correct hypovolemia, maintenance of electrolyte balance, correction of acidosis, early diagnosis and supportive treatment of complications [9,12,28–30] Analgesia goals are the same in both groups, with regular evaluation of pain, at least every 4 hours. In conscious and communicating patients, a visual analogue score (VAS) for pain below 40/100 is targeted and a behavioural pain scale (BPS) of 3-4 is targeted in non-communicating patients.[31,32] In both groups, a stepped multimodal approach to pain

management will be applied based on routine protocols from each participating centre, and combining opioid, non-opioid +/- adjuvant drugs administered through the oral, enteral and/or intravenous routes, as recommended by the World Health Organization's pain relief ladder.[12,33]

The interventional group consists in applying standard care combined with thoracic EA through an epidural catheter placed in an intervertebral space between the 6th and the 9th thoracic vertebra, and administration of a mixed solution of ropivacaine (2 $mg.mL^{-1}$) and sufentanil (0.5 $\mu g.mL^{-1}$), for at least 72 hours. EA will be provided using a patient-controlled epidural analgesia (PCEA) device, with continuous infusion rate of 5 to 15 $mL.h^{-1}$ and *bolus* of 3 to 10 mL every 10 minutes maximum. If the patient is not able to self-administer EA, nurses are encouraged to administer *boli* to achieve analgesia goals if necessary. In addition, iterative epidural administrations of clonidine (1 $\mu g.kg^{-1}$) may be used by attending physicians to achieve analgesia goals.[34] The drugs used during EA in this trial will be provided in an unblinded manner by the department of Pharmacy at CHU Clermont-Ferrand to all participating centers.

Because of insufficient evidence regarding the optimal duration of EA in ICU patients,[17,18,35] total duration of EA will be chosen by participating physicians for each patient, given that it has been administered for at least 72 hours. Weaning of EA and removal of epidural catheter will be conducted accordingly to recommendations and routine protocols from each participating centre.

Study data are prospectively collected and managed by trained research coordinators and/or investigators from each participating centre, using REDCap electronic data capture tools hosted at CHU Clermont-Ferrand. [36] REDCap (Research Electronic Data Capture) is a secure, web-based application designed to support data capture for research studies, providing: 1) an intuitive interface for validated data entry; 2) audit trails for tracking data manipulation and export procedures; 3) automated export procedures for seamless data downloads to common statistical packages; and 4) procedures for importing data from external sources.

The following data are collected and registered at ICU admission and upon inclusion: baseline demographics and characteristics (age, sex, weight, height, body temperature, delay between the onset of AP and ICU admission/study inclusion, comorbidities and coexisting conditions), baseline severity of illness (modified Marshall scoring system, Simplified Acute Physiologic Score (SAPS) II, Sequential Organ Failure Assessment (SOFA)), usual clinical and biological variables that are measured in critically ill patients, organ failure and treatments. From inclusion to day 30 will be assessed: survival status, main complications of AP (e.g., organ failure, sepsis), the need for therapeutic interventions (such as surgery or endoscopic manoeuvres, MV (either invasive or noninvasive), vasopressor support, continuous renal replacement therapy and/or antibiotic therapy), duration of MV if required, length of stay in the ICU/hospital. These data will be collected at day 0, 1, 2, 3, 4, 5, 6, 7, 17, and 30.

In addition to the data described above, biomarker assays are planned as a future research prospect of the EPIPAN trial.

Biological samples will be collected in each participating center, prior to shipment of all samples, at the end of the patient recruitment period, to the Department of Medical Biochemistry and Molecular Biology at CHU Clermont-Ferrand for blinded measurements. The persons in charge of the biological collection are the coordinating investigator and Dr Roszyk (Laboratory of Medical Biochemistry and Molecular Biology of Pr Sapin, CHU Estaing, CHU Clermont-Ferrand).

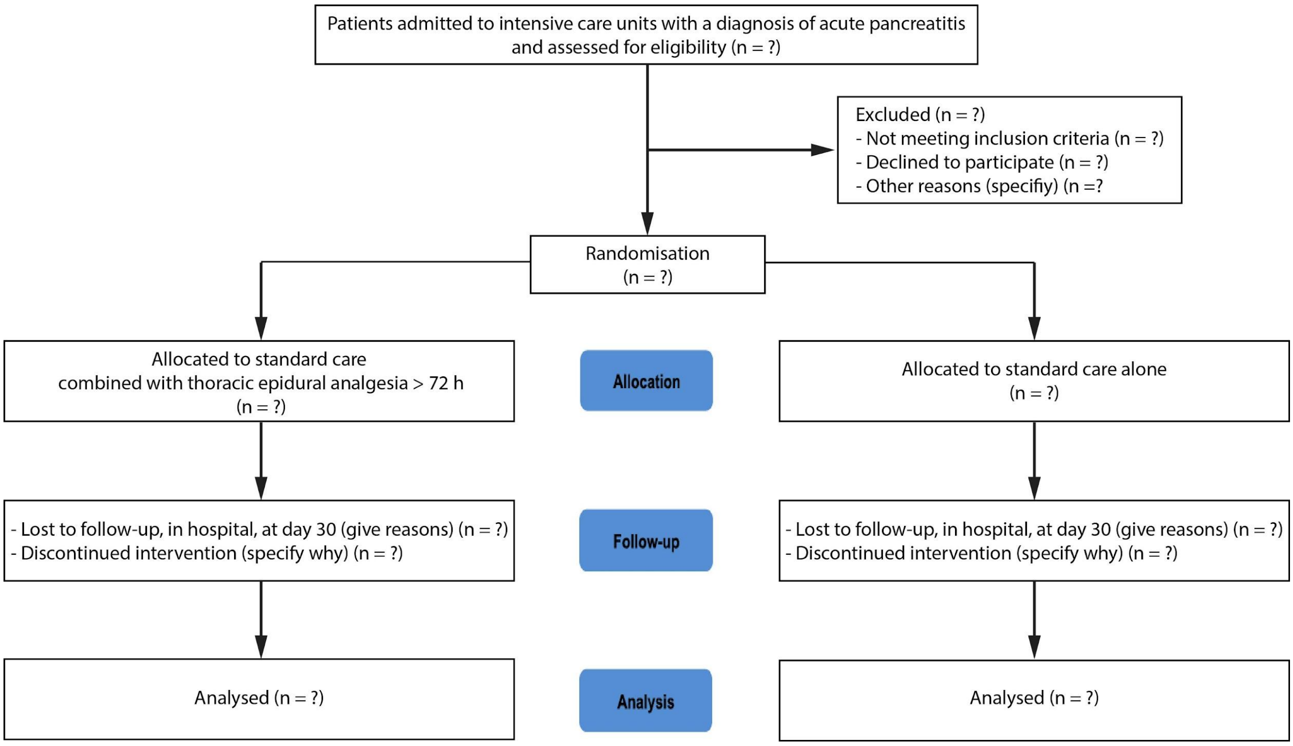

CONSORT diagram of the study illustrating the randomisation and flow of patients in the study.

7.3. Biological sampling and analysis for the study

Creation of a biological collection from blood and urine samples taken from an indwelling arterial catheter (considered as usual care in participating ICUs) during a blood sample procedure as scheduled according to current practice in participating units.

Blood and urine will be sampled at days 0, 2, and 7. Therefore, a total of 3 blood samples of 9 mL each (27 mL total) and of 3 urine samples of 1 mL each (3 mL; in Monovette tubes) will be taken during the first week after inclusion.

Measurements will be performed in duplicate at each timepoint for:

- plasma interleukine (IL-)6, a proinflammatory cytokine, using ELISA (R&D Systems, Minneapolis, Minnesota, USA)
- plasma soluble receptor for advanced glycation end-products (sRAGE), a marker of lung alveolar epithelial injury, using ELISA (R&D Systems, Minneapolis, Minnesota, USA)
- plasma neutrophil gelatinase-associated lipocalin (NGAL), a biomarker of acute kidney injury, using the *Triage Meter* (Alere, San Diego, California, USA)
- urine TIMP-2 and IGFBP-7, biomarkers of acute kidney injury, using the NEPHROCHECK Test System (Biomérieux, Marcy-l'Étoile, France)

The constitution and conservation of the biological collection after the assays described above have been carried out will allow subsequent biological analyses related to the biological mechanisms to be carried out and to be defined, analyses related to future AP and EA research. The consent from patients for such future biological analyses (as not yet specified) will be sought at the time of inclusion in the EPIPAN study.

7.4. Différence par rapport à la prise en charge de routine.

Par rapport à la prise en charge de routine, la participation à cette étude implique pour le patient :

- De bénéficier d'une analgésie péridurale (uniquement dans un groupe)
- La réalisation de 3 prélèvements sanguins de 9 ml chacun (à J0, J2 et J7) à partir d'un cathéter artériel déjà en place.
- La réalisation de 3 prélèvements urinaires de 3 mL chacun, sur tube monovette (à J0, J2 et J7)
- Un contact téléphonique à J30, si le patient n'est plus hospitalisé, afin de s'enquérir de son état de santé.

Dans les centres participant à cet essai, le traitement à l'étude (l'APD) est une technique d'analgésie régulièrement utilisée chez les patients présentant une pancréatite aiguë. Il n'est donc pas à considérer comme un surcoût.

7.5. Expected duration of participation of individuals and description of the trial chronology

Estimated duration of the study: 5 years
Study start date (first patient included): June 2014
Estimated end date (end of follow-up of the last patient enrolled in the study): July 2019
Total duration of patient participation in the study: 31 days

The date of completion of the study will be transmitted to the competent regulatory authority (ANSM) and the Ethics committee (CPP) within 90 days.
In case of premature termination of the study, the information will be transmitted within 15 days to the ANSM and CPP.

8. Intervention (drug / medical device / other) under investigation

8.1. Description of the treatment

All patients will be managed by attending physicians as recommended in recent consensual guidelines on the management of severe AP (standard care): early enteral nutrition when possible, resuscitation measures to correct hypovolemia, maintenance of electrolyte balance, correction of acidosis, early diagnosis and supportive treatment of complications. [9,12,28–30] Analgesia goals are the same in both groups, with regular evaluation of pain, at least every 4 hours. In conscious and communicating patients, a visual analogue score (VAS) for pain below 40/100 is targeted and a behavioural pain scale (BPS) of 3-4 is targeted in non-communicating patients.[31,32] In both groups, a stepped multimodal approach to pain management will be applied based on routine protocols from each participating centre, and combining opioid, non-opioid +/- adjuvant drugs administered through the oral, enteral and/or intravenous routes, as recommended by the World Health Organization's pain relief ladder.[12,33]

The interventional group consists in applying standard care combined with thoracic EA through an epidural catheter placed in an intervertebral space between the 6th and the 9th thoracic vertebra, and administration of a mixed solution of ropivacaine (2 mg.mL^{-1}) and sufentanil (0.5 µg.mL^{-1}), for at least 72 hours. EA will be provided using a patient-controlled epidural analgesia

(PCEA) device, with continuous infusion rate of 5 to 15 $mL.h^{-1}$ and *bolus* of 3 to 10 mL every 10 minutes maximum. If the patient is not able to self-administer EA, nurses are encouraged to administer *boli* to achieve analgesia goals if necessary. In addition, iterative epidural administrations of clonidine (1 $\mu g.kg^{-1}$) may be used by attending physicians to achieve analgesia goals.[34] The drugs used during EA in this trial will be provided in an unblinded manner by the department of Pharmacy at CHU Clermont-Ferrand to all participating centers.

Because of insufficient evidence regarding the optimal duration of EA in ICU patients,[17,18,35] total duration of EA will be chosen by participating physicians for each patient, given that it has been administered for at least 72 hours. Weaning of EA and removal of epidural catheter will be conducted according to recommendations and routine protocols from each participating centre.

Main potential side effects of thoracic EA are pruritus (14%), nausea (11%), transient paresis of the lower limbs (1%), and hypotension (2.4%). The need for naloxone, a morphine-antagonist, is exceptional and is usually associated with significant overdose. Only the risk of urine retention remains frequent, but it is routinely and constantly monitored in the ICU setting. In addition, a significant number of ICU patients have a bladder catheter.

Contraindications to the EA technique are rare and mainly include contraindications to the puncture of the epidural space (see non inclusion criteria). Epidural catheter insertion and removal must be performed during isocoagulation, according to the rules of good practice for peri-medullary anaesthesia.

8.2. Dosage, method of administration and duration of treatment

Analgesia goals are the same in both groups, with regular evaluation of pain, at least every 4 hours. In conscious and communicating patients, a visual analogue score (VAS) for pain below 40/100 is targeted and a behavioural pain scale (BPS) of 3-4 is targeted in non-communicating patients.[31,32] In both groups, a stepped multimodal approach to pain management will be applied based on routine protocols from each participating centre, and combining opioid, non-opioid +/- adjuvant drugs administered through the oral, enteral and/or intravenous routes, as recommended by the World Health Organization's pain relief ladder.[12,33]

The interventional group consists in applying standard care combined with thoracic EA through an epidural catheter placed in an intervertebral space between the 6th and the 9th thoracic vertebra after local anesthesia, and administration of a mixed solution of ropivacaine (2 $mg.mL^{-1}$) and sufentanil (0.5 $\mu g.mL^{-1}$), for at least 72 hours. EA will be provided using a patient-controlled epidural analgesia (PCEA) device, with continuous infusion rate of 5 to 15 $mL.h^{-1}$ and *bolus* of 3 to 10 mL every 10 minutes maximum. If the patient is not able to self-administer EA, nurses are encouraged to administer *boli* to achieve analgesia goals if necessary. In addition, iterative epidural administrations of clonidine (1 $\mu g.kg^{-1}$) may be used by attending physicians to achieve analgesia goals.[34] The drugs used during EA in this trial will be provided in an unblinded manner by the department of Pharmacy at CHU Clermont-Ferrand to all participating centers.

Because the trial was primarily designed as a pragmatic trial, all patients will be managed by attending physicians as recommended in recent consensual guidelines on the management of severe AP (standard care): early enteral nutrition when possible, resuscitation measures to correct hypovolemia, maintenance of electrolyte balance, correction of acidosis, early diagnosis and supportive treatment of complications. [9,12,28–30]

8.3. Presentation of the drugs

The Summaries of Product Characteristics (French official *Résumés des Caractéristiques des Produits RCP*) for drugs administered via the epidural route are available on the ANSM website.

SUFENTANIL (morphine derivative)

Morphine derivatives provide good quality, predictable and long-lasting analgesia by the epidural route. Epidurally administered sufentanil does not cause motor or sympathetic block and does not result in loss of thermal or tactile sensitivity. Only the perception of painful stimuli is diminished.

ROPIVACAINE (local anesthetic)

Ropivacaine provides long-lasting analgesia with a larger sensory block than motor block. Tachyphylaxis for ropivacaine is less a risk than with other short-acting amide radical local anaesthetics (lidocaine, prilocaine and mepivacaine). Ropivacaine has a similar pharmacological profile to bupivacaine, which remains the most widely used local anesthetic, but its toxicity, particularly cardiac, is less. At the same concentration, the motor block is also less intense.

COMBINATION OF LOCAL ANAESTHETICS AND MORPHINE DERIVATIVES

The combination of local anaesthetics and morphine derivatives by the epidural route dhas a synergistic effect that improves the quality of analgesia and allows the dose of each product to be reduced. Numerous publications have shown the effectiveness of such combinations with resting pain scores below 20 mm on a visual analogue scale (VAS) for the majority of patients and better analgesia on mobilization than that obtained with other techniques. The main advantage of this combination is a reduction in side effects related to local anaesthetics (such as motor block, hypotension, tachyphylaxis).

CLONIDINE (adjunct to loco-regional analgesia)

Epidural clonidine acts on alpha-2 adrenergic receptors in the posterior horn of the marrow, and also has its own analgesic effect, probably by acting on muscarinic and nicotinic receptors. The mechanism of action is different from that of morphine derivatives and local anaesthetics, but it potentiates the analgesic action of local anaesthetics and prolongs the sensory block. Its action begins within 15 to 20 minutes, with a peak of action between 60 and 90 minutes after punctual epidural injection. The side effects of epidural clonidine are hypotension and sedation. This sedative effect is sometimes sought in situations where epidural analgesia has been inadequate for some time.

8.4. Issuance and compliance

THE PRESCRIPTION: It is carried out by an authorised professional, specialised in anesthesiology and critical care medicine, and depends on the benefit-risk ratio for the patient and according to the randomization arm.

DISPENSATION: Only the pharmacist is authorized to dispense medication. However, pharmacy interns, fifth-year university hospital students and pharmacy assistants may, in part, dispense under the responsibility of the pharmacist.

- Prescribed medicines are dispensed within a period of time appropriate to their use in a nominative manner from the prescriptions. Medicines are prepared for each patient, at variable intervals (daily, weekly, etc.), if possible on a per-use basis; the dispensing of

medicines is recorded and traceability is ensured in accordance with the regulations in a computerized manner.
- Information and advice on the proper use of the medicinal product should be provided to clinical professionals and patients by pharmacy staff under the responsibility of the pharmacist. They shall be subject to traceability.
- The person in charge of transporting drugs from the pharmacy to the clinical areas is identified. The transport conditions guarantee safe and hygienic transport.

THE ADMINISTRATION: As the treatment is only administered by paramedical or medical resuscitation teams, no control of treatment compliance is foreseen. All participating centers will have the drugs planned for this project (ropivacaine, sufentanil and clonidine) at their disposal.

STOCK MANAGEMENT IN CLINICAL AREAS: The stock management is established according to terms and conditions determined by pharmacy and clinical care units.

THERAPEUTIC MONITORING OF THE PATIENT : Therapeutic monitoring of the patient makes it possible to evaluate the benefit rendered and to identify the possible occurrence of any adverse effects, including minor ones. The latter are recorded in the patient's file, reported to the Regional Pharmacovigilance Center, serious adverse reactions, unexpected adverse reactions and harmful and unintended reactions resulting from misuse.

8.5. Drugs and treatments allowed or not allowed during the trial

No drugs or treatments are prohibited during the EPIPAN trial.

9. Collected data

The clinico-biological data collected during the study by the investigators and clinical research associates are those that are usually collected in the medical record of an ICU patient. Data will be anonymized by the study-specific code assigned to each upon randomization, and include:
- baseline demographics and characteristics (age, sex, weight, height, body temperature, delay between the onset of AP and ICU admission/study inclusion, comorbidities and coexisting conditions)
- baseline severity of illness (modified Marshall scoring system, Simplified Acute Physiologic Score (SAPS) II, Sequential Organ Failure Assessment (SOFA))
- usual clinical and biological variables that are measured in critically ill patients, cause of AP, organ failure and treatments.

From inclusion to day 30 will be assessed: survival status, main complications of AP (e.g., organ failure, sepsis), the need for therapeutic interventions (such as surgery or endoscopic manoeuvres, MV (either invasive or noninvasive), vasopressor support, continuous renal replacement therapy and/or antibiotic therapy), duration of MV if required, length of stay in the ICU/hospital. Biological samples will be collected in each participating centre, prior to shipment of all samples to the Department of Medical Biochemistry and Molecular Biology at CHU Clermont-Ferrand for blinded measurements.

10. Statistical Methods

10.1. Sample size estimation

According to previous studies from the literature,[11,19] we have estimated that a sample size of n = 74 patients per group would provide 80% statistical power to detect an absolute between-group difference of 7 days (with a SD of ± 15) in the primary outcome, i.e. in the number of VFD at day 30 after randomization (expected number of VFD at day 30: 20 ± 15 vs. 13 ± 15 in the intervention and control arms, respectively), for a two-sided type I error of 5%.

Given theoretical concerns related to possible adverse effects of EA in ICU patients, an interim safety analysis will be performed after data for 74 patients are collected, taking into account the inflation of the 1st species error risk (α=0.03, Kim-DeMets, software ©East). The independent Data and Safety Monitoring Board (DSMB) will recommend that the trial be stopped if it is found that the conduct of the trial compromises patient safety (a between-group difference in mortality or VFD at day 30).

10.2. Statistical analysis

A predefined statistical analysis plan will be followed. Statistical analyses will be conducted using Stata software (version 14, StataCorp, College Station, USA). A two-sided p-value of less than 0.05 will be considered to indicate statistical significance.

Concerning the primary outcome, the comparison between interventional and reference groups will be analysed using Student’s t-test or Mann-Whitney's test if assumptions of t-test are not met. Normality will be studied by the Shapiro-Wilk test and homoscedasticity using the Fisher-Snedecor test. Results will be expressed as effect-sizes and 95% confidence intervals. Intention to treat (ITT) analysis of data from all randomised patients (except patients who withdraw their consent and those who do not meet the inclusion criteria), including those from the interventional group who do not receive EA for at least 72 hours, will be considered for the primary analysis. Then, the analysis of the primary outcome will be completed by multivariate analysis using a linear mixed model to take into account: (1) fixed effects covariates determined according to univariate results and to clinical relevance (duration of symptoms (either above or below 48 hours from first symptoms, e.g. abdominal pain, to inclusion) and severity of AP as assessed by the modified Marshall scoring system for organ dysfunction) and (2) centre as random-effects (to measure between and within centre variability). The normality of residuals will be studied as described previously. Results will be expressed as regression coefficients and 95% confidence intervals.

Other continuous endpoints (e.g., level of sedation using the Richmond Agitation-Sedation Scale, analgesia scores, doses of drugs, length of stay in ICU/hospital, levels and kinetics of biological markers, duration of MV, and healthcare-related costs at day 30) will be analysed in the same way.

Categorical parameters (death, organ failure, severe sepsis, septic shock, ARDS, the need for MV, acute respiratory failure, abdominal compartment syndrome, intra- or extra-abdominal sepsis, pancreas necrosis (infected or not) as assessed by computed tomography, hemodynamic failure requiring vasopressor support, acute kidney injury, the need for renal replacement therapy, intra-abdominal collection requiring radiological, surgical or endoscopic drainage) will be analysed using Chi-squared or Fisher's exact tests for univariate analysis and generalized linear mixed model (logistic for dichotomous dependent endpoint or Poisson if more appropriate) for multivariate analysis. Type I error will be adjusted using the Hochberg method if appropriate. Results will be expressed as relative risks and 95% confidence intervals. These data will also be analysed as censored data, when appropriate; survival analyses will be performed with the Kaplan-Meier

estimator and differences between groups will then be assessed using the log-rank test. The assumption of log-linearity of risk and the proportional hazards will be checked beforehand. Results will be expressed as hazard ratios and 95% confidence intervals. The tolerance of enteral nutrition and/or the incidence of signs of gastrointestinal intolerance (nausea, vomiting, and ileus) will be analysed similarly.

Longitudinal analyses of repeated measures (levels on days 0, 2 and 7 after inclusion of biological markers of systemic inflammation, lung epithelial injury and acute kidney injury will be studied using random-effect models (linear or generalized linear), to take into account patients as random-effect (slope and intercept), nestled in centre random-effect.

According to clinical relevance and to CONSORT recommendations, subgroup analyses depending on the presence or the absence of epidural analgesia will be proposed after the study of subgroup x randomisation group interaction in regression models.

Per-protocol analyses will also be conducted after intention-to-treat analysis is performed. Results from per-protocol analyses will be compared to those from intention-to-treat analyses. A particular focus will be given to safety and patients who are lost to follow-up. A sensitivity analysis will be performed and the nature of missing data will be studied (missing at random or not). According to this study, the most appropriate approach to the imputation of missing data will be proposed (maximum bias (e.g., last observation carried forward vs. baseline observation carried forward) or estimation proposed by Verbeke and Molenberghs for repeated data).

10.3. Data management and analysis

Responsible person;
Bruno PEREIRA, PhD Biostatistics
Délégation Recherche Clinique & Innovation
CHU de Clermont-Ferrand - Villa annexe IFSI
58, Rue Montalembert, 63003 Clermont-Ferrand cedex, France
Mail : bpereira@chu-clermontferrand.fr / Tel : (33) 473 754 964

11. Safety assessment – Management of adverse events

The investigator is responsible for reporting all adverse events.

11.1. Definitions

Adverse event: any untoward medical occurrence in a patient or clinical investigation subject administered a pharmaceutical product and which does not necessarily have to have a causal relationship with the research or with this treatment.

Adverse effect: any untoward response related to the research.

Serious adverse effects are sub-grouped as follows:

- ***Expected*** *serious adverse event*: any event that is described in the most recent version of the Investigator's Brochure, or in the Summary of Product Characteristics for marketed medicinal products, or in the instruction notice when the research concerns a medical device which is subject to CE marking. This definition also applies to an investigational medicinal product when administered for a same population outside the labeled indications.

- ***Unexpected*** *serious adverse event*: any event, the nature, severity or outcome of which is

not consistent with the information in the most recent version of the Investigator's Brochure or the Summary of Product Characteristics for a marketed medicinal product or the information notice for a medical device.

Serious adverse event or effect: any undesirable event or effect which results in death, is life-threatening, requires in-patient hospitalization or prolongation of existing hospitalization, results in persistent or significant disability/incapacity, or is a congenital anomaly/birth defect.

The term "life-threatening" refers to an event in which the patient was at risk of death at the time of the event, independently of the consequences of corrective or palliative treatment.

The terms "*disability*" or "*incapacity*" refer to any clinically significant, temporary or persistent disability.

Death, regardless of the cause, including when it corresponds to progression of the disease under treatment, is considered a serious adverse event.

Other events which do not correspond to the above definitions can be considered "*potentially serious*", in particular certain laboratory anomalies. The investigator or sponsor's medical judgement can result in such events being reported in the same manner as "serious" events. It is necessary for study protocols to specify the characteristics of "potentially serious" events that are subject to reporting.

New information: event concerning the conduct of the research or the development of the medicinal product or related product, which is the object of the research, when said new information may jeopardize the safety of the research subjects. Examples include:

- an increase in the rate of occurrence of serious events;
- results of interim analyses, when relevant to the safety of the research subjects (notably a lack of efficacy);
- serious adverse events related to the clinical trial procedures;
- lack of efficacy with a medicinal product used to treat life-threatening disease;
- a major safety finding from animal studies that provides new information on the safety of the product;
- and generally, any new information that could lead to an unfavorable reassessment of the benefit/risk ratio of the research.

Any new information concerning the research (or the product used) which may jeopardize the safety of the research subjects will be subjected to appropriate urgent measures and prompt and timely notification by the Sponsor to the competent authority and the Ethics Committee.

11.2. Serious adverse event reporting

It is the investigator's obligation to report within 24 hours any serious adverse event occurring in any patient enrolled in a study:

- During the active phase of the study,
- In the weeks following cessation of treatment,
- Within the deadlines established for safety monitoring off treatment, before (wash-out or withdrawal phase) or after the active phase,
- After termination of the study, regardless of the time of the event, when no cause other than the research can reasonably be incriminated,
- On the "Serious adverse event report form", indicating the date of onset, the severity, the causal relationship with the treatment (or product), and the follow-up/outcome.

The narrative describing the event should be completed and transmitted to the sponsor as soon as new, relevant information is received. Depending on the nature and seriousness of the event, copies of the patient's anonymized medical record can be attached, as well as laboratory results.

When a serious adverse event persists at the end of the study, the investigator will continue to follow the patient until said event is considered resolved.

In accordance with the implementing decree 2006-477 of 26/04/2006 amending chapter 1 of title II of Book I of the first part of the Public Health Code relating to biomedical research, all suspected unexpected serious adverse effects must be reported by the sponsor to ANSM and to the Ethics Committee at first knowledge and no later than:

- 7 days after occurrence in case of death or a life-threatening event
- 15 days after occurrence for all other unexpected serious adverse events (SAE).

The sponsor will decide upon the significance of the serious adverse events that it reports and the consequences thereof, in particular with respect to the conduct of the research.

The sponsor will also assess the causality of the adverse event with the research by means of a joint analysis with the Regional Pharmacovigilance Center.

The sponsor will maintain a detailed list of all adverse events reported by the investigator(s).

Once per year, or on request, the sponsor will submit an annual safety update report to ANSM and to the Ethics Committee containing all available safety information.

The sponsor will also provide the investigators with any information that may affect the safety of the research subjects.

In this study, only severe adverse events related to EA are potentially expected. Complications of AP will be collected (see full list in Annex 1).

11.3. Independent data monitoring and safety board (DMSB)

A steering committee will be set up to finalize and validate the protocol and the eCRF, and will monitor the progress of the study in order to facilitate the development of the project. It will be able to make decisions concerning the course of the study and if deemed necessary, to propose amendments to the protocol, decide on the opening or closing of centers and discuss the strategy for publishing the results at the end of the trial.

An independent DSMB, composed of three experts (Prs. Hervé Dupont, Thomas Lescot and Philippe Montravers) will monitor the safety of the trial. The DSMB will be responsible for safeguarding the interests of trial participants, assessing the safety and efficacy of the interventions during the trial, and for monitoring the overall conduct of the clinical trial. To contribute to enhancing the integrity of the trial, the DSMB may also formulate recommendations relating to the recruitment/retention of participants, their management, improving adherence to protocol-specified regimens and retention of participants, and the procedures for data management and quality control.

11.4. End of the study / Study interruption

The research will be supervised by a steering committee made up of the various research partners: coordinating investigator, methodologist, sponsor representatives, etc.

The steering committee will be composed of sponsor representatives and the research team.

The steering committee may be asked to decide whether to temporarily or permanently halt the trial based on advice from the independent monitoring committee, such as in the event of a

significant difference in favor of one of the two study groups (after interim analysis) or because of patient safety issues (serious adverse events).

If he/she wishes, a patient included in the study may, at any time, withdraw consent with no need for justification of his/her decision.

The termination of participation may also be decided for medical reasons on the initiative of the research investigators in charge of the patient.

11.5. Follow-up of subjects with an adverse event

All patients with an adverse event will be monitored until the event is resolved or stabilized.

12. Right of access to source document and data

12.1. Access to data

The sponsor is responsible for obtaining the agreement of all parties involved in the research in order to guarantee direct access to all study sites, source data, source documents and reports for purposes of the sponsor's quality control and audit.

The investigators will provide access to the documents and individual data that are strictly necessary for purposes of monitoring, quality control and audit of the biomedical research, to the persons authorized to consult said documents pursuant to the legislative and regulatory provisions in force (articles L.1121-3 and R.5121-13 Public Health Code).

12.2. Source data

Source documents, defined as any original document or object which proves the existence or accuracy of data or information recorded during the clinical study, will be stored for a period of 15 years by the investigator or by the hospital in the case of a hospital medical record.

12.3. Data confidentiality

Subject to the provisions relating to the confidentiality of data to which persons in charge of quality control of biomedical research have access (article L.1121-3 Public Health Code), and subject to the provisions relating to the confidentiality of information as concerns in particular the nature of the products being studied, the trials, the persons undergoing the research and the results obtained (article R.5121-13 Public Health Code), persons having direct access shall take all necessary precautions to ensure the confidentiality of the information relating to the products being studied, the trials, the persons undergoing the research and notably their identity, and the results obtained.

These persons, as well as the investigators themselves, are bound by professional secrecy (in accordance with the conditions laid down in articles 226-13 and 226-14 of the penal code).

During the biomedical research or upon its completion, the data collected on the research subjects and transmitted to the sponsor by the investigators (or any other specialized study staff) shall be rendered anonymous.

In no case shall the names or addresses of the persons undergoing the research appear.

Anonymity of the subjects will be guaranteed by the creation of a subject identifying number.

The sponsor will ensure that each research subject has given his written consent allowing access to his personal data, which is strictly necessary for quality control of the research.

12.4. Registration in the national file of biomedical research subjects

Not applicable (no participation of healthy volunteers).

13. Quality control and assurance

13.1. Engagement of the investigators and the sponsor of the study

The investigator undertakes to conduct the study in compliance with public health law 2004-806 of 9 August 2004 relating to biomedical research, the implementing decree 2006-477 of 26/04/2006 amending chapter 1 of title II of book 1 of the first part of the Public Health Code relating to biomedical research, and with the bylaws in force.
The study will also be conducted in compliance with Good Clinical Practices for biomedical research on medicinal products for human use, as laid down in article L.1121-3 Public Health Code and the decree of 24 November 2006.
The investigator also undertakes to comply with the Declaration of Helsinki of the World Medical Assembly (Tokyo 2004, revision).

13.2. Quality assurance

Clinical Research Associates (CRAs) designated by the sponsor will ensure the proper conduct of the study, the collection of data generated in writing, and their documentation, recording and reporting, as per the Standard Operating Procedures in effect at the CHU Clermont-Ferrand and in compliance with Good Clinical Practices and legislative and regulatory provisions in force.

13.3. Quality control

The investigator guarantees the authenticity of the data collected during the study and accepts the legal provisions authorizing the study sponsor to implement quality control.

The coordinating investigator and associated investigators therefore agree to make themselves available during Quality Control visits by the Clinical Research Associate that will be scheduled at regular intervals. The following items will be examined at each visit:

- Informed consent
- Compliance with the study protocol and procedures
- Quality of data recorded in the case report forms: accuracy, missing data, coherence with source documents (medical records, appointment calendars, original copies of laboratory results, etc.)
- Management of any study products

13.4. Case report form

In order to make the collection and analysis of the data collected during the study easier and more reliable, the free and secure web-based application system REDCap (for Research electronic Data Capture), developed at Vanderbilt University (Nashville, Tennessee, USA), is used. The observation logbook will thus be in electronic format (eCRF).

All the information required by the protocol must be recorded in the eCRFs (secure electronic REDCap system) by the investigating physician at each participating centre, and an explanation

must be provided for any missing data. Data should be collected as they are obtained, and transcribed into the logbooks in a clear and legible manner.

Data found to be erroneous in the case report forms should be clearly corrected and the new data should be copied, together with the corrected information, accompanied by the electronic signature of the investigator, the date, and possibly a justification by the investigator or the authorised person who made the correction.

The anonymity of the subjects shall be ensured by means of a code number and the initials of the investigator on all documents necessary for the research, including electronic documents, or by erasure by appropriate means of the nominative data on copies of the source documents, intended to

to the research literature.

The computerized data on a file will be declared to the CNIL according to the procedure adapted to the case.

14. Ethical considerations

14.1. Ethics Committee and Medicine Agency

The study protocol, patient information notice and consent form will be submitted to the designated *Comité de Protection des Personnes* (CPP).

Notification of a favorable opinion from the Ethics Committee will be transmitted to the sponsor and to ANSM. The sponsor of the study will send an authorization request to ANSM prior to study start. The study sponsor is defined as per law 2004-806 (August 9, 2004).

14.2. Information for patients and written informed consent form

Patients will be fully and fairly informed, in understandable terms, of the objectives and constraints of the study, the possible risks involved, the necessary monitoring and safety measures, their rights to refuse to participate in the study or the possibility of withdrawing at any time. The investigator should also inform the subjects of the opinion given by the PPC.

Oral and written information about the study will be given at the time of admission to resuscitation. The patient's informed consent will be obtained prior to epidural analgesia. In the event that the patient is unable to give free informed consent himself (in the case of confusional syndrome or sedation for example), the consent of his trusted person will be sought. In the absence of the appointment of a trusted person, or if the trusted person is not present or cannot be contacted, or if no close relative or family member of the patient is present or can be reached, an emergency consent procedure will be carried out with the participation of a physician who is independent of the trial and who is not directly responsible for the patient, in order to confirm the patient's eligibility for the study. In cases where the patient himself/herself has not been able to give consent to participate in the study, his/her consent for continued participation in the study will be sought as soon as his/her state of health permits.

All this information is included on an information and consent form given to the patient. The free, informed and written consent of the patient will be obtained by the investigator. These documents are approved by the competent CPP and are to be used for the trial concerned, to the exclusion of all other documents.

Two original copies shall be co-signed by the investigator and the patient. One copy will be given to the patient and the second copy will be kept in the patient's medical file.

14.3. Protocol amendments

Protocol amendments must be qualified as substantial or non-substantial. According to their nature, they will be the object of a new Ethics Committee opinion and/or authorization from the competent authority.

14.4. Management of the patient enrolled in the study

See section 8 (Intervention (drug / medical device / other) under investigation).

15. Data processing and storage of study documents

15.1 Data entry and processing

At each participating center, data will be collected and entered into the electronic web-based case report form (eCRF) by trial or clinical trained personal (clinical research associate), blinded to the allocation group, under the supervision of the trial site investigators.

Data analysis (including the interim analysis after 50% inclusions are performed) will be carried out at the Biostatistical and Data Management Unit, Department of Clinical Research and Innovation (DRCI), CHU Clermont-Ferrand, by Dr Bruno Pereira, PhD in Biostatistics.

15.2 CNIL

This study enters within the scope of “Reference Methodology” in application of the provisions of the law of 6 August 2004 relating to the protection of natural persons with regard to the processing of personal data and amending the law of 6 January 1978 relating to computer processing, data files and civil liberties. This change was approved by decision of 5 January 2006. The CHU Clermont-Ferrand, which sponsored the study, has signed a commitment to comply with this “Reference Methodology” on March, 15, 2007.

15.3 Data retention and archiving

The following documents will be archived under the study name in the Department of Perioperative Medicine (Prof. Jean-Etienne Bazin, Head of the Department of Perioperative Medicine, CHU Clermont-Ferrand, Clermont-Ferrand, France) until the end of the period of practical usefulness (60 months, including inclusion of patients and data analysis).

These documents are:

Protocol and appendices, and any amendments,

Signed, original information notices and consent forms,

Individual data (authenticated copies of raw data),

Follow-up documents

Statistical analyses

Final study report

At the end of the period of practical usefulness, all documents to be archived, such as defined in procedure PG.06.005 “Management of documentation relating to protocols” of Clermont-Ferrand Hospital will be transferred to the central archives and placed under the sponsor’s responsibility for a period of 15 years after study completion, in accordance with institutional practices. These documents cannot be moved or destroyed without the sponsor’s permission. After the 15 years are up, the sponsor will be consulted for destruction. All the data as well as all documents and reports may be subject to audit or inspection.

16. Funding and insurance

16.1 Estimated cost of the study

The total estimated cost includes :

- the cost of plasma biomarker assays, estimated at 15220 euros
    - sRAGE (R&D Systems): 82 effective doses per kit (unit price including VAT: 450 euros), i.e. 11 kits required for 888 doses (148 patients, duplicate doses, 3-step); estimated cost: 4950 euros
    - IL-6 (R&D Systems): 82 effective doses per kit (unit price including VAT: 530 euros), i.e. 11 kits required for 888 doses (148 patients, duplicate doses, 3-step); estimated cost: 5830 euros.
    - NGAL (Alere): 888 effective doses (unit price including VAT per dose: 5 euros; 148 patients, duplicate doses, 3-step); estimated cost: 4440 euros.
- the cost of the constitution, management and conservation of the biological collection, estimated at 15 euros per patient, i.e. 2220 euros in total.
- the cost of repatriating the samples to Clermont-Ferrand (in one go for each centre), estimated at 600 euros.

The overall cost of this study is therefore estimated at 18,000 euros.

There are multiple requests for funding for the study (co-funding):

- from the CHU of Clermont-Ferrand (AOI 2014, Dr Jabaudon)
- from the French Society of Anesthesia and Resuscitation (SFAR 2014 research grant, Dr Jabaudon)
- with the European Society of Intensive Care Medicine (ESICM) (European Critical Research Network Awards 2014, Dr Jabaudon)

In the event that all or part of this co-funding is not obtained, the investigator undertakes to finance by other means all or part of the study budget as presented above (e.g. through the participation of the Department of Perioperative Medicine, CHU Clermont-Ferrand in clinical studies funded by industrial partners and unrelated to the current research project).

16.2 Study insurance

CIn accordance with regulatory provisions, the CHU Clermont-Ferrand, in its capacity as sponsor, has taken out civil liability insurance covering any damages resulting from the research with the Société Hospitalière d’Assurances Mutuelles (SHAM).

It should be noted that non-observance of the legal conditions of the research (absence of Ethics Committee opinion, absence of ANSM authorization, non-consent of subjects, continuation of a suspended or prohibited study) shall render this coverage void.

17. Communication - Rules for publication

The study will be registered prior to first inclusion on www.clinicaltrials.gov. The study protocol will also be published in a peer-reviewed journal.
The sponsor is the owner of the data and no use or transmission to a third party may be made without its prior consent. The first authors of the publications will be the persons who actually participated in the elaboration of the protocol and its progress as well as in the drafting of the results. A writing committee will be set up including members of the steering committee and investigators to define the order of signatories for this study.
Data will only be disclosed after prior joint agreement of the coordinating investigator, the steering committee and the sponsor. The results will be the subject of communications and publications.

18. Feasibility

18.1. Competence of the teams involved

The framework of the research and its conditions are those of daily practice for acute pancreatitis. The investigators in charge of protocol implementation in each of the centers participating in the research have clinical research experience. Measurements for the assessment criteria will be carried out by professionals trained for this purpose in each centre (anesthesiologists and/or intensive care specialists).

18.2 Recruitment feasibility

The 60 month-duration of the study will be consistent with the recruitment goals of the centers participating in the research. Participating ICUs have high volumes of patient admissions for AP. Taking into account patients who cannot be analyzed at the end of the study, an inclusion of at least 30 patients per center is anticipated for the feasibility of this protocol.

19. Annexes

Annex 1: Definitions of AP-related Complications (Secondary Endpoint Measures) (in French)

Annex 2: Participant timeline

ANNEX 1

DEFINITIONS DES COMPLICATIONS DES PANCREATITES AIGUES

(CRITERES DE JUGEMENT SECONDAIRES)

→ **Ventilation non invasive pour détresse respiratoire aiguë** définie par une polypnée > 25/min avec mise en jeu des muscles respiratoires accessoires ou un pH < 7,25 et un Syndrome de Détresse Respiratoire Aigue (Définition de Berlin) définit par une détresse respiratoire de moins d'une semaine avec un rapport PaO2/FiO2 < 300 mmHg avec une PEEP ou cPAP ≥5 cm d'H2O (43)

→ **Ventilation invasive (intubation) pour détresse respiratoire aiguë** définie par une polypnée > 25, mise en jeu des muscles respiratoires accessoires ou un pH < 7,25 et un Syndrome de Détresse Respiratoire Aigue (Définition de Berlin) définit par une détresse respiratoire de moins d'une semaine avec un rapport PaO2/FiO2 < 300 mmHg avec une PEEP ou cPAP ≥5 cm d'H2O (22)

→ **Défaillance hémodynamique nécessitant un traitement par amines : insuffisance** circulatoire aigue, avec trouble de la perfusion tissulaire qui entraîne une anoxie cellulaire avec déviation anaérobie du métabolisme

→ **Décès**

→ **Syndrome du compartiment abdominal** se définit par une augmentation rapide et incontrôlée de la pression intra-abdominale au delà de 20 mmHg associée, avec un lien de causalité, à au moins une défaillance d'organe

→ **Nécrose pancréatique** de tout ou partie de la glande pancréatique et par des **coulées** inflammatoires extra-pancréatique

→ L' **Insuffisance rénale aiguë** (44) est défini comme

1. une augmentation de la créatinine de ≥ 26.5 µmol/l sur une période de 48h
2. ou une augmentation de la créatinine de ≥ 1.5 fois la valeur de base sur une période de 7 jours;
3. ou une diminution de la diurèse de < 0.5 ml/kg/h sur 6h

→ **Sepsis** défini par la présence d'un SIRS en réponse à un processus infectieux confirmé ; une infection suspectée ou prouvée, ou un syndrome clinique pathognomonique d'une infection.

→ **Sepsis sévère** défini par la présence sepsis avec dysfonction d'organe, hypotension artérielle ou hypoperfusion. **Un état de choc septique** est défini par la présence d'un sepsis avec une hypotension ou une hypoperfusion réfractaire malgré un remplissage vasculaire adéquat et la mise en route de catécholamines en perfusion continue après la chirurgie; le signe d'une hypoperfusion systémique peut être une dysfonction d'organe ou la présence d'un taux de lactate sanguin > 4 mmol/dl. Les autres signes incluent une oligurie, une altération des fonctions cognitives.

ANNEX 2

PARTICIPANT TIMELINE

	Inclusion (day 0)	Day 1	Day 2	Day 3	Day 4	Day 5	Day 6	Day 7	Day 15	Day 30
Informed consent	X									
Eligibility: check inclusion and exclusion criteria	X									
Randomisation	X									
Filling of case report forms (including data on EA in the interventional group)	X	X	X	X	X	X	X	X	X	X
Sampling of blood and urine specimens	X		X					X		
Complications of acute pancreatitis and survival status									X	X
End of study										X

Summary of changes to the Study Protocol

Version 1 (November 7, 2013)

Version 2 (January 3, 2024)

- Version of the protocol including the information and consent forms, prior to the submission to the Ethics committee for approval.

Version 3 (February 14, 2024)

- "Consolidated" version of the protocol after being approved by the Ethics committee.

Version 4 (May 16, 2014)

- 2 participating centres (Brussels and Lyon) and 3 investigators (Pr. Laterre, Dr. Friggeri, Dr. Wallet) added.
- Details added on the study population: « severe acute pancreatitis » defined defined as acute pancreatitis that is managed in the intensive care unit
- Secondary outcome measure added: opioid drugs and doses administered for analgesia within 7 days from randomisation
- Change in randomisation strata prior to recruitment start because the cause of acute pancreatitis (biliary, alcohol, other) is not associated with clinical outcomes in the literature. This stratum has been replaced by "the degree of severity according to the Marshall score (3 strata of increasing severity according to the maximum score obtained for at least one of the functions evaluated (respiratory, renal, hemodynamic): 0, 1-2, 3-4)"
- Details added on the electronical tool used for randomization: "Tenalea web application v4.0.5090.3953, Alex Data management"
- Urine samples (3 x 3 mL) added to the biological collection on days 0, 2, and 7
- Details added for the study's clinical follow-up: on days "0, 1, 2, 3, 4, 5, 6, 7, 15, and 30".

Version 5 (August 28, 2014)

- 1 participating centre (Nancy) and 1 investigator (Dr. Guerci) added.

Version 6 (July 8, 2016)

- 2 participating centres (Annecy, Nice) and 2 investigators (Dr. Danin Dr. Escudier) added.

Version 7 (January 20, 2017)

- Extension of the duration of the study (to a total of 60 months)

Version 8 (July 3, 2018)

- Change in a participating centre (Lyon) and investigator (Dr. Bonnassieux)

2. STATISTICAL ANALYSIS PLAN

- **Statistical Analysis Plan** (*version July 3, 2018*).

- **Final (Modified) Statistical Analysis Plan** *(version February 3, 2021)*.

- **Summary of Changes to the Statistical Analysis Plan**

- **References**

Original Statistical Analysis Plan *(version July 3, 2018)*

<u>Sample Size Estimation</u>

Assuming a mean (±standard deviation) number of 13±15 ventilator-free days in the standard care group [1, 2], we determined that a sample size of 148 patients would provide the trial with a power of 80% to detect an absolute between-group difference of 7±15 ventilator-free days at day 30 after randomisation with a two-sided type-I error rate (alpha) of 0.05 [1].

An interim analysis of the primary endpoint was performed after 50% of the patients had completed 30 days of follow-up. The interim analysis was performed by an independent statistician who was unaware of the treatment assignments by applying the Lan and DeMets approach with stopping boundaries for efficacy at a P value of less than 0.03.

<u>Generalities</u>

All analyses will be performed with the use of Stata software (version 15, StataCorp) and of R, version 4.0.5 (R Foundation for Statistical Computing) before the breaking of the randomisation code, according to International Conference on Harmonisation-Good Clinical Practice guidelines.

The primary analysis will be conducted in the modified intention-to-treat (ITT) population. A per-protocol analysis will also be conducted on the primary outcome. The criteria for including patients in the modified ITT and in the per-protocol populations, respectively, are provided below.

Baseline variables will be reported as numbers and percentages for categorical variables and medians with interquartile ranges [IQRs] for continuous variables. According to the CONSORT 2010 statement, group differences in baseline variables will not be compared using significance testing unless specifically requested by peer reviewers.

Subgroup analyses will be performed to explore the potential influence of prespecified factors on the primary outcome.

A particular focus will be given to safety and patients who are lost to follow-up. A sensitivity analysis will be performed, and the nature of missing data will be studied (missing at random or not). According to this study, the most appropriate approach to the imputation of missing data will be proposed (maximum bias (eg, last observation carried forward vs. baseline observation carried forward) or estimation proposed by Verbeke and Molenberghs for repeated data).

A two-sided P value of less than 0.05 will be considered for statistical significance of all analyses (except the interim analysis).

Study populations

Intention-to treat (ITT) population: All randomised patients (except those who had withdrawn consent for the use of their data or did not meet the inclusion criteria retrospectively), including those from the interventional group who did not receive epidural analgesia for at least 72 hours.

Per-protocol population: All randomised patients except patients having one or more major protocol violations, defined as patients who would not be eligible for randomisation according to inclusion/exclusion criteria or patients who would have withdrawn consent.

Primary analysis

The comparison between randomisation groups will be analysed using Student's t-test or the Mann-Whitney test if assumptions of the t-test are not met. Normality will be studied by the Shapiro-Wilk test. Homoscedasticity will be analysed using the Fisher-Snedecor test

Effect-sizes will be estimated with 95% confidence intervals (CI).

Secondary analyses

The analysis of the primary outcome will be complemented by multivariable analysis using a linear mixed model to consider:

(1) Fixed effects covariates determined according to univariate results (P values to enter variables into the model will be P<0.10) and to clinical relevance (duration of symptoms <48 *vs.* ≥48 hours from first symptoms to enrollment, severity of acute pancreatitis as assessed by the modified Marshall scoring system for organ dysfunction [3];

(2) Site as random effect (to measure between and within centre variability).

Results will be expressed as regression coefficients and 95% CI.

Other continuous endpoints (such as the level of sedation using the Richmond Agitation-Sedation Scale, analgesia scores, doses of drugs, length of stay in intensive care unit/hospital, levels and kinetics of biological markers, duration of mechanical ventilation, and healthcare-related costs at day 30) will be compared between randomisation groups using Student's t-test or the Mann-Whitney test if assumptions of the t-test are not met for univariate analyses and using linear mixed model for adjusted analyses.

Categorical parameters (such as death, organ failure, severe sepsis, septic shock, ARDS, the need for mechanical ventilation, acute respiratory failure, abdominal compartment syndrome, intra- or extra-abdominal sepsis, pancreas necrosis (infected or not) as assessed by computed tomography scan, hemodynamic failure requiring vasopressor support, acute kidney injury, the need for renal replacement therapy, intra-abdominal collection requiring radiological, surgical or endoscopic drainage) will be analysed using Chi-squared or Fisher's exact tests for univariate analysis, and generalised linear mixed model (logistic for dichotomous dependent endpoint or Poisson if more appropriate) for multivariable analysis. Results will be expressed as relative risks and 95% CI.

Survival analyses will be performed with the Kaplan-Meier estimator and differences between groups will then be assessed using the log-rank test. The assumption of

log-linearity of risk and the proportional hazards will be checked beforehand. Results will be expressed as hazard ratios and 95% CI.

Longitudinal analyses of repeated measures (such as the levels of biological markers of systemic inflammation, lung epithelial injury and acute kidney injury on days 0, 2, and 7 after inclusion) will be performed using random effect models (linear or generalised linear) to consider patients as random effect (slope and intercept), nested in site’s random effect.

According to clinical relevance and to CONSORT recommendations, subgroup analyses depending on the presence or absence of epidural analgesia will be proposed after the study of subgroup x randomisation group interaction in regression models.

Modified Statistical Analysis Plan *(version February 3, 2021)*

Sample Size Estimation

Assuming a mean (±standard deviation) number of 13±15 ventilator-free days in the standard care group [1, 2], we determined that a sample size of 148 patients would provide the trial with a power of 80% to detect an absolute between-group difference of 7±15 ventilator-free days at day 30 after randomisation with a two-sided type-I error rate (alpha) of 0.05 [1].

An interim analysis of the primary endpoint was performed after 50% of the patients had completed 30 days of follow-up. The interim analysis was performed by an independent statistician who was unaware of the treatment assignments by applying the Lan and DeMets approach with stopping boundaries for efficacy at a P value of less than 0.03.

Generalities

All analyses will be performed with the use of Stata software (version 15, StataCorp) and of R, version 4.0.5 (R Foundation for Statistical Computing) before the breaking of the randomisation code, according to International Conference on Harmonisation-Good Clinical Practice guidelines.

The primary analysis will be conducted in the modified intention-to-treat (ITT) population. A per-protocol analysis will also be conducted on the primary outcome. The criteria for including patients in the modified ITT and in the per-protocol populations, respectively, are provided below.

Baseline variables will be reported as numbers and percentages for categorical variables and medians with interquartile ranges [IQRs] for continuous variables. According to the CONSORT 2010 statement, group differences in baseline variables will not be compared using significance testing unless specifically requested by peer reviewers.

Subgroup analyses will be performed to explore the potential influence of prespecified factors on the primary outcome.

A particular focus will be given to safety and patients who die or are lost to follow-up by day 30 after randomisation. No imputation approach will be used if less than 5% of data are missing.

A two-sided P value of less than 0.05 will be considered for statistical significance of all analyses (except the interim analysis). Because of the potential for type-I error due to multiple comparisons, findings from analyses of secondary endpoints will be interpreted as exploratory.

Study populations

Intention-to treat (ITT) population: All randomised patients (except those who had withdrawn consent for the use of their data or did not meet the inclusion criteria retrospectively), including those from the interventional group who did not receive epidural analgesia for at least 72 hours.

Per-protocol population: All randomised patients except patients having one or more major protocol violations, defined as patients who would not be eligible for randomisation according to inclusion/exclusion criteria or patients who would have withdrawn consent.

Primary analysis

The comparison between randomisation groups will be performed with the Mann-Whitney U test. Effect-sizes or absolute median differences will be estimated with 95% confidence intervals (CI).

Secondary analyses

Given the distribution of the primary outcome measure due to a large proportion of patients with a total of 30 ventilator-free days, the odds ratio for having 30 ventilator-free days and

the incident rate ratio for the number of ventilator-free days (when not equal to 30) will be computed using zero-inflated negative binomial regression.

The analysis of the primary outcome will be complemented by multivariable analysis using multiple zero-inflated negative binomial mixed regression after adjustment for the randomisation-stratification variables, including site as random effect.

A second multivariable model included the site as random effect, the randomisation-stratification variables, and covariates determined according to univariate results and clinical relevance (age, male sex, the need for vasopressor support or intubation, the presence of sepsis or peripancreatic necrosis, and opioid use at baseline) will be carried out.

Other continuous endpoints (such as death, organ failure, severe sepsis, septic shock, ARDS, the need for mechanical ventilation, acute respiratory failure, abdominal compartment syndrome, intra- or extra-abdominal sepsis, pancreas necrosis (infected or not) as assessed by computed tomography scan, hemodynamic failure requiring vasopressor support, acute kidney injury, the need for renal replacement therapy, intra-abdominal collection requiring radiological, surgical or endoscopic drainage) will be compared between randomisation groups using Student's t-test or the Mann-Whitney test if assumptions of the t-test are not met. Results will be expressed as effect-sizes and 95% CI.

Categorical parameters (such as death, organ failure, severe sepsis, septic shock, ARDS, the need for mechanical ventilation, acute respiratory failure, abdominal compartment syndrome, intra- or extra-abdominal sepsis, pancreas necrosis (infected or not) as assessed by computed tomography scan, hemodynamic failure requiring vasopressor support, acute kidney injury, the need for renal replacement therapy, intra-abdominal collection requiring radiological, surgical or endoscopic drainage) will be analysed using Chi-squared or Fisher's exact tests for univariate analysis. The results will be expressed as absolute differences and

relative risks with 95% CI estimated using a generalised linear model (more precisely, Poisson with robust variance).

Survival analyses will be performed with the Kaplan-Meier estimator and differences between groups will then be assessed using the log-rank test. The assumption of log-linearity of risk and the proportional hazards will be checked beforehand. Results will be expressed as hazard ratios and 95% CI.

Longitudinal analyses of repeated measures (such as the levels of biological markers of systemic inflammation, lung epithelial injury and acute kidney injury on days 0, 2, and 7 after inclusion) will be expressed as descriptive results.

According to clinical relevance and to CONSORT recommendations, subgroup analyses depending on the presence or absence of epidural analgesia will be proposed after the study of subgroup x randomisation group interaction in regression models. For unadjusted subgroup analysis of the primary endpoint, zero-inflated negative binomial regression will be performed.

Summary of Changes to the Statistical Analysis Plan

- Update in the version of Stata software (v15) and R, version 4.0.5 (R Foundation for Statistical Computing) added for statistical analyses.
- Details added for missing data/patients lost to follow-up: « No imputation approach will be used if less than 5% of data are missing. »
- Details added for multiple comparisons: « Because of the potential for type-I error due to multiple comparisons, findings from analyses of secondary endpoints will be interpreted as exploratory. »
- Details added on secondary analyses of the primary endpoint, given data monitoring showing a large proportion of patients with a total of 30 ventilator-free days, i.e., patients alive on day 30 after randomisation and who never required intubation and mechanical ventilation through day 30 after randomisation. Therefore, zero-inflated negative binomial regression was added as a secondary model to evaluate the primary outcome measure.
- Details added on multivariable adjustments for analysis of the primary outcome, using two multiple zero-inflated negative binomial mixed regression models:
    - 1/ adjustment for the randomisation-stratification variables, including site as random effect,
    - 2/ adjustment for the site as random effect, the randomisation-stratification variables, and covariates determined according to univariate results and clinical relevance.
- Details added on secondary continuous endpoints (expressed as effect-sizes and 95%CI): the level of sedation using the Richmond Agitation-Sedation Scale, analgesia scores, doses of drugs, length of stay in intensive care unit/hospital, levels and kinetics of biological markers, duration of mechanical ventilation, and healthcare-related costs (in secondary analysis) at day 30

- Details added on secondary categorical endpoints (expressed as relative risks and 95% CI): death, organ failure, severe sepsis, septic shock, ARDS, the need for mechanical ventilation, acute respiratory failure, abdominal compartment syndrome, intra- or extra-abdominal sepsis, pancreas necrosis (infected or not) as assessed by computed tomography scan, hemodynamic failure requiring vasopressor support, acute kidney injury, the need for renal replacement therapy, intra-abdominal collection requiring radiological, surgical or endoscopic drainage.
- Details added on longitudinal analyses of repeated measures (the levels of biological markers of systemic inflammation, lung epithelial injury and acute kidney injury on days 0, 2, and 7 after inclusion), and reported as descriptive results.

References

1. Jung B, Carr J, Chanques G, et al (2011) [Severe and acute pancreatitis admitted in intensive care: a prospective epidemiological multiple centre study using CClin network database]. Ann Fr Anesth Reanim 30:105–112

2. Sadowski SM, Andres A, Morel P, et al (2015) Epidural anesthesia improves pancreatic perfusion and decreases the severity of acute pancreatitis. World J Gastroenterol 21:12448–12456

3. Banks PA, Bollen TL, Dervenis C, et al (2013) Classification of acute pancreatitis--2012: revision of the Atlanta classification and definitions by international consensus. Gut 62:102–111
